# Energy management system for multi interconnected microgrids during grid connected and autonomous operation modes considering load management

**DOI:** 10.1038/s41598-024-72952-5

**Published:** 2024-10-17

**Authors:** H. K. Shaker, H. E. Keshta, Magdi A. Mosa, A. A. Ali

**Affiliations:** 1 Faculty of Engineering, Helwan University, Helwan, Egypt; 2 Faculty of Engineering at Shoubra, Benha University, Banha, Egypt

**Keywords:** Microgrids, One to one based optimizer, Distributed energy resources, Economic dispatch, Energy management, Muti-microgrid, Autonomous mode, Grid connected, Engineering, Electrical and electronic engineering

## Abstract

This study focuses on improving power system grid performance and efficiency through the integration of distributed energy resources (DERs). The study proposes an artificial intelligence (AI) based effective approach for economic dispatch and load management for three linked microgrids (MGs) that operate in both grid-connected and autonomous modes. A day-ahead scheduling method is suggested to calculate the optimal set points for various energy sources in MGs considering various system constraints for safe operation. In addition, a load management approach that shifts the controllable loads from one interval to another is applied to reduce the operating cost of MG. To handle the optimization challenges of energy scheduling and load shifting such complexity and non-linearity, an advanced meta-heuristic method known as the one-to-one based optimizer (OOBO) is used. Overall, the paper proposes a viable and efficient methodology for economical distribution in linked microgrids, which takes advantage of renewable energy resources and incorporates scheduling optimization via the OOBO algorithm. The proposed energy management strategy enhances the system performance, increases energy efficiency, and reduces the daily operational cost by 1.6% for grid connected mode and by 0.47% for islanded operation mode.

##  Introduction

A microgrid is a small-scale power system unit comprising of distributed generations (DGs) (like photovoltaic (PV), wind turbine (WT), fuel cell (FC), micro gas turbine (MGT), and diesel generator), energy storage (like batteries), and loads piled in close proximity to each other. Microgrids improve the reliability and the resilience of power system grid in case of severe conditions and emergency situations^1,2^. They provide many benefits either environmental or economical like enhancement of energy efficiency, reduce transmission losses as distributed energy resources (DERs) are located close to the load, reduction of greenhouse gases (GHG) (CO2 emission) and provide energy to remote communities. A microgrid can run in two modes of operation, in tandem with the grid (grid connected) or autonomously from the grid (islanded mode), and it can be AC MG, DC MG, or hybrid combination (both AC and DC)^3–5^. Interconnecting many MGs creates a cluster of MGs, and each individual system can profit from this collaboration in grid-connected and autonomous modes. Each MG in the network can buy or sell extra energy to meet its own power needs.

Many research papers with different control techniques for multi microgrids (MMGs) that allow deliberate energy interchange between them, although they ignore the economic dispatch and unit commitment issues. Like in^6^ this paper uses the fuzzy proportional integral based model reference adaptive controller (FPI- MRAC) to satisfy the load demand by controlling the power interchange between the different MGs ignoring the economic dispatch problem. The active power control at DC- side of PV generation for frequency enhancement in islanded MGs as in^7^. In^8^ a fuzzy fractional order proportional integral derivative controller (FFOPID) is used for frequency enhancement in isolated MG system. A fuzzy logic control (FLC) type-3 is used in^9^ for current sharing in DC microgrids and voltage balancing. A multi-microgrid system’s dynamics are improved, with microgrids exchanging power between them for AC/DC MGs using voltage and frequency control as indicated in^10^. An Adaptive technique for frequency regulation in multi-area microgrids with renewable energy resources and electric vehicles assisted by virtual inertia as indicated in^11^.

Meta-heuristic algorithms have lately gained popularity as an effective technique for solving economic dispatch problems. In^12^ a particle swarm optimization (PSO) technique is used for demand side response energy management for microgrids model depending on multi agent system (MAS). An energy management model based on an artificial neural network (ANN) technique is provided in^13^ and the model is optimized by PSO technique. A model predictive control (MPC) is used for the strategy of power management in microgrids using PSO as an optimization technique^14^. A FLC for energy management system (EMS) for microgrid with the optimization technique genetic algorithm (GA) for modeling the EMS^15^. In^16^ the genetic algorithm is used to tackle the research’s multi-objective optimization challenges for demand side energy management of microgrids. An improved adaptive GA used for solving the optimal EMS for grid-connected two microgrids as indicated in^15^. In^17^ a modified manta ray foraging (MRF) optimization technique is used for an efficient energy management of microgrid completed with renewable energy. utilizing the flower pollination algorithm (FPA) is utilized for optimal size selection and energy management of islanded hybrid system forming of photovoltaic and wind turbine system^18^. In^19^ an aquila optimizer (AO) technique is utilized for demand response of a residential MG system. In^20^ a mixed integer linear programming (MILP) model is provided in IEEE 33 bus system for reshaping the demand load profile in order to decrease electricity waste in an electricity distribution network (EDN). In order to reduce the total cost associated with consumption of energy the MILP model is used for optimally managing storage batteries and a collection of household appliances, including charging electric vehicles (EVs), in a neighborhood energy storage community (NESC) powered entirely by the power grid as indicated in^21^.

In this work a novel metaheuristic technique is used to obtain the optimal set point of the different sources used in the proposed system of three interconnected multi–MGs. the metaheuristic technique is one to one based optimizer (OOBO), that can tackle with different optimization difficulties and can give more adequate, acceptable, and satisfied results compared with other optimization techniques^22,23^. The OOBO is used to obtain the optimal set point of the two batteries in both MG1 and MG2. While the Lagrange multiplier approach. is used to obtain the optimal set point of the Diesel generators of MG1, MG2, and micro gas turbine of MG3 as indicated in the proposed model in the following section during both modes of operation, Grid connected mode and autonomous mode.

The main contributions of this work can be listed as:

(i) proposing an effective day-ahead scheduling for three-interconnected multi-MGs based on a cutting-edge meta-heuristic technique, OOBO, with the goal of minimizing daily operating costs by calculating the optimal set points of the different energy sources. The optimal set-points for diesel generators and MGT are obtained using the Lagrange multiplier approach.
(ii) Applying a load-shifting technique-based load management approach to reduce the operational costs of a multi-interconnected microgrid during both grid-connected and islanded operation modes.

The remainder of the paper is organized as follows: Section "Multi-microgrid system" illustrates the components of the proposed three- interconnected MGs system. Section "The energy management system (EMS)" elaborates the optimization technique used to obtain the optimal set point of the proposed system. Section "One to one based optimizer (OOBO)" elaborates the EMS for the proposed system for the most efficient technical operation including optimal set points, EMS cost function, and restrictions of the different components of the proposed system. The obtained simulation results are reviewed and assessed in section "Simulation results". The conclusion from this work is discussed in the last section "Conclusion".

## Multi-microgrid system

**Fig. 1:**
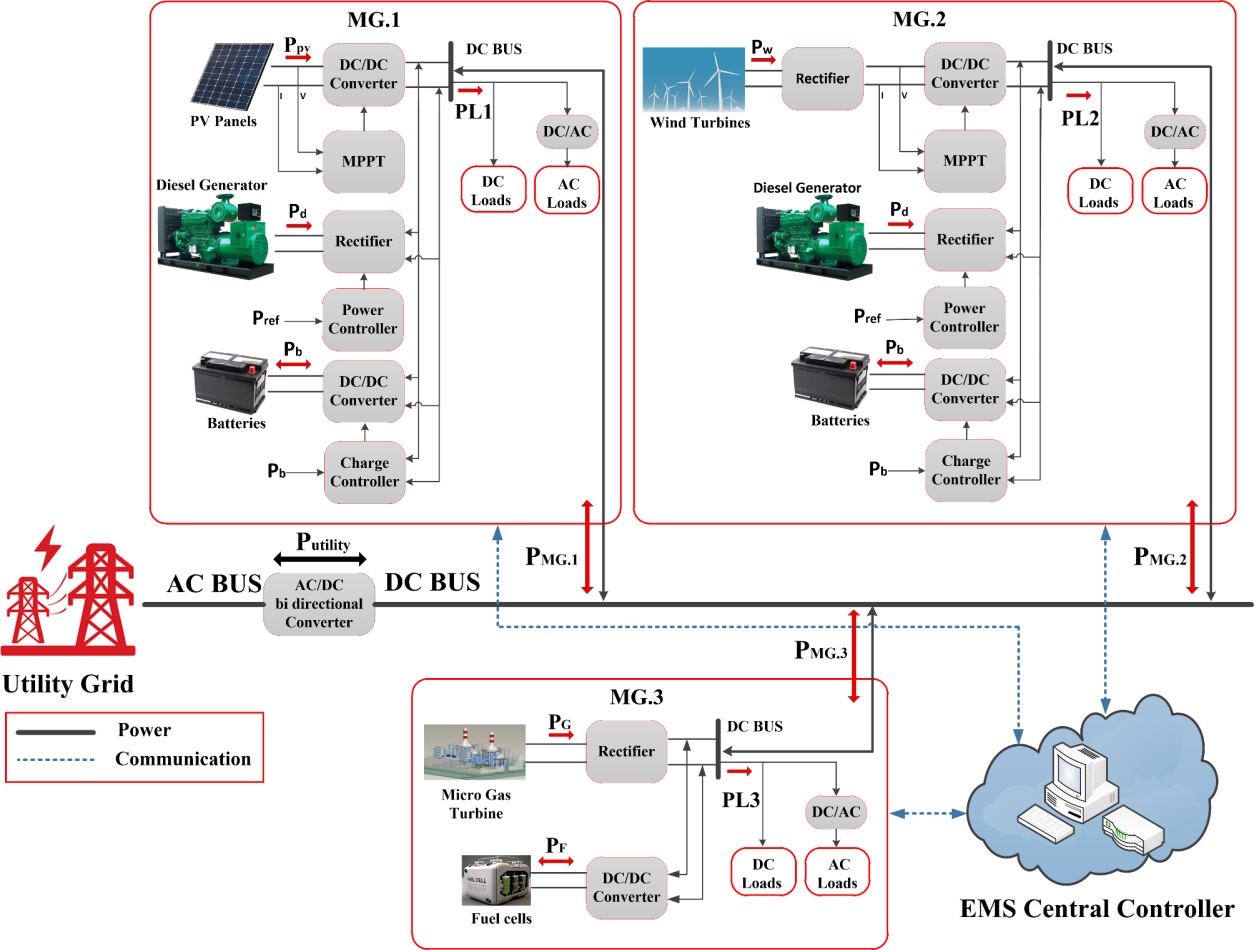
Proposed model of three interconnected MGs.

The proposed system that consists of three MGs as indicated in Fig. 1. The first MG consists of a PV system of 500 kW that is connected to the DC bus via a DC-to-DC converter (boost converter), Diesel generator of 1000 kW that is connected to the DC bus via AC-to-DC converter (rectifier), and battery of 1800 kWh that is connected to the DC bus via a DC-to-DC converter (bidirectional buck boost converter). The second MG consists of a wind turbine of 1000 kW that is connected to the DC bus via a AC-to-DC converter (rectifier) then DC-to-DC converter (boost converter), Diesel generator of 1000 kW that is connected to the DC bus via AC-to-DC converter (rectifier), and battery of 1800 kWh that is connected to the DC bus via a DC-to-DC converter (bidirectional buck boost converter). The third MG consists of a gas turbine generator of 1500 kW that is connected to the DC bus via AC-to-DC converter (rectifier), and fuel cell of 1000 kW that is connected to the DC bus via a DC-to-DC converter (boost converter). Then after connecting the three MGs to the DC bus, the connection between them and utility grid is done via DC-to-AC bidirectional converter.

Each individual source in the three MGs operates in its own fashion. For example, a battery has two operating modes, one is charging mode, and the other is discharging mode, but a WT system or PV system can run in either maximum power point tracking (MPPT) mode or limitation power mode. The two diesel generators and micro gas turbine can function within the power constraints set by the controller. Hence, each source has its own local agent. The EMS central controller calculates the optimal set-points of sources and controllable loads in the day ahead and allows the three MGs to communicate with one another to ensure optimal performance depending on the data forecasted from the irradiance of solar, the speed of wind, the price of electricity, and the profile of the load. The output data is then directed to the local agents at sources and loads within the MG for the efficient operation.

MG’s operating considerations vary depending on the mode of operation. The most effective system operation strategies prioritize cost-effectiveness, dependability, and low emissions. The optimal dispatch challenge of DG units in MGs is to minimize generation costs and reduce environmental pollution. An MG’s generation expenses include fuel, emissions, operation, maintenance, and purchasing/selling costs. To assess the cost of MG generation, the proposed methodology took into account the power production of renewable energy DGs and the fuel cost of non-renewable energy DGs^24,25^.

### PV system

The PV array consists of numerous PV modules connected in parallel and series. This turns the incident solar irradiance into photovoltaic current. The extracted power from PV module is illustrated in the following equation^25^:

1 $$\:{\text{P}}_{\text{p}\text{v}}={\text{P}}_{\text{s}}\:\:\frac{\text{G}}{{\text{G}}_{\text{s}\text{d}}}\:\:[\:1+\text{K}\left({\text{T}}_{\text{c}}-\:{\text{T}}_{\text{r}}\right)]$$

Where: G is the real irradiance, $\:{\text{G}}_{\text{s}}$ is irradiance at STC, Ps is highest output power during STC (standard temperature condition), K is Power temperature factor, $\:{\text{T}}_{\text{c}}$ is cell temperature, and $\:{\text{T}}_{\text{r}}$ is reference temperature. The parameters are ( $\:{\text{G}}_{\text{s}}$= 1000 W/m^2^, K = 0.005, Ps = 500 kW, and $\:{\text{T}}_{\text{r}}$ = 25 OC)

### Wind turbine system

Wind turbines convert wind’s kinetic energy (KE) into mechanical energy. The power extracted from wind turbine depend on wind speed as indicated in the following equation^25^:

2 $$\:\left\{\begin{array}{c}{\text{P}}_{\text{w}}=0\:\:\:\:\:\:\:\:\:\:\:\:\:\:\:\:\:\:\:\:\:\:\:\:\:\:\:\:\:\:\:\:\:\:\:\:\:\:\:\:\:\:\:\:\:\:\:\:\:\:\:\:\:\:\:\:\:\:\:\:\:\:\:\:\:\:\:\:\:\:\:\:\:\:\:\:\:\:\:\:\:\:\:{\text{V}}_{\text{w}\:\:}<\:{\text{V}}_{\text{w}\text{c}\text{i}\:\:}\:\\\:{\text{P}}_{\text{w}}={\text{C}}_{1}.{\text{V}}_{\text{w}}^{3}+\:{\text{C}}_{2}{.\text{V}}_{\text{w}}^{2}+{\text{C}}_{3}{.\text{V}}_{\text{w}}+{\text{C}}_{4}\:\:\:\:\:\:\:\:\:\:\:\:\:\:\:\:{\text{V}}_{\text{w}\text{c}\text{i}\:\:}\le\:\:\:{\text{V}}_{\text{w}\:\:}<\:{\text{V}}_{\text{w}\text{r}\:\:}\\\:{\text{P}}_{\text{w}}={\text{P}}_{\text{w}\text{r}}\:\:\:\:\:\:\:\:\:\:\:\:\:\:\:\:\:\:\:\:\:\:\:\:\:\:\:\:\:\:\:\:\:\:\:\:\:\:\:\:\:\:\:\:\:\:\:\:\:\:\:\:\:\:\:\:\:\:\:\:\:\:\:\:\:{\text{V}}_{\text{w}\text{r}\:\:}\le\:\:{\text{V}}_{\text{w}\:\:}<\:{\text{V}}_{\text{w}\text{c}\text{o}\:\:}\\\:{\text{P}}_{\text{w}}=0\:\:\:\:\:\:\:\:\:\:\:\:\:\:\:\:\:\:\:\:\:\:\:\:\:\:\:\:\:\:\:\:\:\:\:\:\:\:\:\:\:\:\:\:\:\:\:\:\:\:\:\:\:\:\:\:\:\:\:\:\:\:\:\:\:\:\:\:\:\:\:\:\:\:\:\:\:\:\:\:\:\:\:\:\:{\text{V}}_{\text{w}\:\:}\ge\:\:{\text{V}}_{\text{w}\text{c}\text{o}\:\:}\end{array}\right.$$

Where: $\:{\text{P}}_{\text{w}\text{r}}$ is the rated power, $\:{\text{V}}_{\text{w}}$ is actual wind speed, $\:{\text{V}}_{\text{w}\text{c}\text{i}\:\:}$is cut in speed, $\:{\text{V}}_{\text{w}\text{c}\text{o}\:\:}$is cut out speed. The parameters are ( $\:{\text{C}}_{1}$= = -2.608, $\:{\text{C}}_{2}$ = 63.201, $\:{\text{C}}_{3}$ = 293.3, $\:{\text{C}}_{4}$ = 374.23, $\:{\text{P}}_{\text{w}\text{r}}$ = 1000 kW, $\:{\text{V}}_{\text{w}\text{c}\text{i}\:\:}$= 3.5 m/s, $\:{\text{V}}_{\text{w}\text{r}\:\:}$= 12.5 m/s, and $\:{\text{V}}_{\text{w}\text{c}\text{o}\:\:}$=20 m/s).

### Deisel generator

The operating cost of diesel generator can be stated as a quadratic polynomial function of its actual output power.as indicated in the following equation^25^:

3 $$\:{\text{C}}_{\text{D}}={\text{a}}_{1}+\:{\text{b}}_{1}.{\text{P}}_{\text{D}}+{\text{c}}_{1}{.\text{P}}_{\text{D}}^{2}$$

Where: a, b, and C are constants that can be obtained from the catalog of diesel generator manufacturer, and $\:{P}_{D}$ is the output power of diesel generator. The parameters are ($\:{\text{P}}_{\text{D}}$= 1000 kW, $\:{\text{a}}_{1}$ = 0.3312, $\:{\text{b}}_{1}$ = 0.0156, and $\:{\text{c}}_{1}$ = 0.0002484)

### Gas turbine generator

The operating cost of a gas turbine generator can be written as a quadratic polynomial function of its actual output power, similar to that of a diesel generator as indicated by Eq. (4) ^25^.

4 $$\:{\text{C}}_{\text{G}}={\text{a}}_{2}+\:{\text{b}}_{2}.{\text{P}}_{\text{G}}+{\text{c}}_{2}{.\text{P}}_{\text{G}}^{2}$$

Where: a, b, and C are constants that can be obtained from the catalog of a gas turbine generator manufacturer, and $\:{P}_{G}$ is the output power of gas turbine generator. The parameters are ($\:{\text{P}}_{\text{G}}$= 1500 kW, $\:{\text{a}}_{2}$ = 0.4969, $\:{\text{b}}_{2}$ = 0.0116, and $\:{\text{c}}_{2}$ = 0.0001987).

### Fuel cell system

A fuel cell is an electrochemical device that uses chemical energy to produce electrical energy. Fuel cells are very efficient and emit no hazardous gases, making them a possible alternative source to conventional combustion engines. The fuel used in this system is hydrogen with cost can be expressed by the following equation^25^:

5 $$\:{\text{C}}_{\text{F}}=\:{\text{b}}_{3}.{\text{P}}_{\text{F}}$$

Where: $\:\text{b}$ is the cost of hydrogen, $\:{\text{P}}_{\text{F}}$ is the power generated from fuel cell. The parameters are ($\:{\text{P}}_{\text{F}}$= 1000 kW, and $\:{\text{b}}_{3}$ = 0.0848).

### Battery system

The state of charge (SoC) of battery in case of charging and discharging mode can be calculated by the Eqs. (6) and (7) as indicated by the following^25–27^:

6 $$\:\text{S}\text{o}\text{C}\left(\text{t}+1\right)=\text{S}\text{o}\text{C}\left(\text{t}\right)+{{\upeta\:}}_{\text{c}\text{h}}\left(\frac{{\text{P}}_{\text{b}\:}}{{\text{W}}_{\text{b}}}\right){\Delta\:}\text{t}$$

7 $$\:\text{S}\text{o}\text{C}\left(\text{t}+1\right)=\text{S}\text{o}\text{C}\left(\text{t}\right)+\frac{{\text{P}}_{\text{b}\:}}{{{\upeta\:}}_{\text{d}\text{c}\text{h}}\times\:{\text{W}}_{\text{b}}}{\Delta\:}\text{t}$$

The cost of battery appeared as quick discharging and charging, is deleterious to battery life, the cost is determined by Eq. (8) as following:

8 $$\:{\text{C}}_{\text{B}\:}={\text{a}}_{3}+{\text{C}}_{3}{.\text{P}}_{\text{b}}^{2}\:\:\:$$

Where: $\:{{\text{C}}_{\text{B}\:},\text{P}}_{\text{b}\:,\:}{{\text{W}}_{\text{b}},{\upeta\:}}_{\text{c}\text{h}},\text{a}\text{n}\text{d}\:{{\upeta\:}}_{\text{d}\text{c}\text{h}}$ are cost, power, capacity, charging, and discharging efficiency of battery respectively. The parameters are ($\:{\text{a}}_{3}$= 0, $\:{\text{C}}_{3}$ = 0.01625, $\:\:{\text{W}}_{\text{b}}$= 1800 kWh, $\:{{\upeta\:}}_{\text{c}\text{h}}$ = 0.9, and $\:{{\upeta\:}}_{\text{d}\text{c}\text{h}}$= 0.9).

## The energy management system (EMS)

The planned EMS operates on a day-ahead basis, with the goal of meeting the required load demand at the lowest possible operational cost based on 24-hour prediction data. Day-ahead scheduling can be achieved through two phases, the first phase is day-ahead unit commitment, and second phase is day-ahead load management.

### Day-ahead unit commitment (phase one)

The EMS’s input data include predicted load demand, extracted power from PV, WT, micro gas turbines, diesel generators, grid tariff, and system restrictions. The EMS’s output data is the scheduled power for the following day, calculated using the lowest energy consumption cost. The suggested economic operating approach involves managing both the electricity generated by DERs in MGs and the power traded among three MGs and the main utility grid^28^.

### Day- ahead load management (phase two)

The second phase is load shifting technique-based demand side management, in which controllable loads are moved from high-cost to low-cost periods to minimize the MG’s energy consumption costs. The desired load curve is oppositely proportional to the MG’s operating cost, which changes depending on the time of usage. The desired result or desired load can be determined as follows:

9 $$\:\text{D}\text{e}\text{s}\text{i}\text{r}\text{e}\text{d}\:\text{l}\text{o}\text{a}\text{d}\:\left(\text{t}\right)=({\text{P}}_{\text{L}\text{t}\text{o}\text{t}}/\text{T}\text{i}\text{m}\text{e}\:)\:\text{x}\:\left(\:{\text{C}\text{o}\text{s}\text{t}}_{\text{a}\text{v}\text{g}}\right)/{\text{C}\text{o}\text{s}\text{t}}_{\text{O}\text{P}}\:\left(\text{t}\right)$$

Where: P_Ltot_ is sum of overall load of MGs (kW), Time is day hours (24 h), $\:{\text{C}\text{o}\text{s}\text{t}}_{\text{a}\text{v}\text{g}}$ is average cost of microgrid tariff all over the day, and $\:{\text{C}\text{o}\text{s}\text{t}}_{\text{O}\text{P}}$ is the running cost of MGs. If the running cost of the MGs is high, the desired load will be low and vice versa.

The suggested single objective function for the optimization problem of load shifting approach can be expressed as follows:

10 $$\:\text{M}\text{i}\text{n}{\sum\:}_{t=1}^{24}{\left({\text{P}}_{\text{L}\text{o}\text{a}\text{d}}\:\right(\text{t})\:-\:\text{D}\text{e}\text{s}\text{i}\text{r}\text{e}\text{d}\:\text{L}\text{o}\text{a}\text{d}\:(\text{t}\left)\right)}^{2}$$

11 $${{\text{P}}_{{\text{Load}}}}\left( {\text{t}} \right)={{\text{P}}_{\text{L}}}\left( {\text{t}} \right)+{\text{conn }}\left( {\text{t}} \right)-{\text{disconn }}\left( {\text{t}} \right)$$

12 $$\left( {0.{\text{9 * }}{{\text{P}}_{\text{L}}}\left( {\text{t}} \right)} \right)~{{\text{P}}_{{\text{Load}}}}\left( {\text{t}} \right)$$

where P_Load_ (t) is actual load after load management at time interval t, P_L_ (t) is required MG load at time interval t, conn (t) is connected load at time interval t, and disconn (t) is disconnected load at time interval t at load management period. In this work, the controlled load is considered to be 10% of overall load^29^.

### Evaluating the optimal operating points for different sources

Calculating the set-points of the different sources for the next day, day-ahead scheduling, using previous data. A novel meta-heuristic algorithm OOBO is suggested to find the optimal operating points of batteries and the transferred electricity with the main grid. It determines the ideal time for charging or discharging the batteries and the ideal time for purchasing or selling power from or to the utility grid in order to reduce day-ahead energy consumption costs. Battery banks can reduce daily running costs by discharging mode during high power consumption and charging during low power consumption periods, when the electricity rates are lowest. While the optimal operating set point for both diesel generators and MGT are obtained using the Lagrange multiplier approach.

### Cost function of the EMS

The single objective function of the optimization problem of day-ahead unit commitment is minimizing the overall operational cost per day as in Eq. (13) that indicated the overall cost during grid connected mode as following:

13 $$\:{\text{C}\text{o}\text{s}\text{t}}_{\text{t}}=\:\sum\:_{\text{t}=1}^{24}{[\:\text{C}\text{o}\text{s}\text{t}}_{\text{g}\text{r}\text{i}\text{d}\:}\left(\text{t}\right)+\:{2\text{*}\text{C}\text{o}\text{s}\text{t}}_{\text{B}\:}\left(\text{t}\right)+2\text{*}{\text{C}\text{o}\text{s}\text{t}}_{\text{D}\:}\left(\text{t}\right)+{\text{C}\text{o}\text{s}\text{t}}_{\text{G}\:}\left(\text{t}\right)+{\text{C}\text{o}\text{s}\text{t}}_{\text{F}\:}\left(\text{t}\right)\:]\:\:\forall\:\:\:\text{t}\:\epsilon\:[\:1,\:24\:]$$

Where:

$$\:{\text{C}\text{o}\text{s}\text{t}}_{\text{t}}$$: is the per day overall operational cost of the system during grid connected mode ($), $$\:{\:\text{C}\text{o}\text{s}\text{t}}_{\text{g}\text{r}\text{i}\text{d}\:}\left(\text{t}\right)$$: is the per day grid power cost (either purchasing or selling cost) ($), $$\:{\text{C}\text{o}\text{s}\text{t}}_{\text{B}\:}\left(\text{t}\right)$$: is the per day battery cost appeared as quick discharging and charging that deleterious to battery life ($), $$\:{\text{C}\text{o}\text{s}\text{t}}_{\text{D}\:}\left(\text{t}\right)$$: is the per day operational cost of the diesel generator ($), $$\:{\text{C}\text{o}\text{s}\text{t}}_{\text{G}\:}\left(\text{t}\right)$$: is the per day operational cost of the micro gas turbine ($), and $$\:{\text{C}\text{o}\text{s}\text{t}}_{\text{F}\:}\left(\text{t}\right)$$: is the per day operational cost of the fuel cell ($).

While the overall cost during islanded mode can be illustrated by Eq. (14) as following:

14 $$\:{\text{C}\text{o}\text{s}\text{t}}_{\text{t}}=\:\sum\:_{\text{t}=1}^{24}\:{[2\text{*}\text{C}\text{o}\text{s}\text{t}}_{\text{B}\:}\left(\text{t}\right)+2\text{*}{\text{C}\text{o}\text{s}\text{t}}_{\text{D}\:}\left(\text{t}\right)+{\text{C}\text{o}\text{s}\text{t}}_{\text{G}\:}\left(\text{t}\right)+{\text{C}\text{o}\text{s}\text{t}}_{\text{F}\:}\left(\text{t}\right)\:]\:\:\forall\:\:\:\text{t}\:\epsilon\:[\:1,\:24\:]$$

### The restrictions of the EMS

The EMS should meet the following technological restrictions for different sources of the proposed system:

- **Micro gas turbine restrictions**.

To extend the MGT’s life, the power produced by MGT should not increase over the power limits specified in Eq. (15) that indicated as following:

15 $$\:{{\text{P}}_{\text{G}\:\text{m}\text{i}\text{n}}\le\:\text{P}}_{\text{G}}\left(\text{t}\right)\le\:{\text{P}}_{\text{G}\:\text{m}\text{a}\text{x}\:}\forall\:\:\:\text{t}\:\epsilon\:[\:1,\:24\:]$$

Where: $\:{\text{P}}_{\text{G}\:\text{m}\text{i}\text{n}}$ refers to minimum power of MGT of about 10% of its rated power (150 kW), and $\:{\text{P}}_{\text{G}\:\text{m}\text{a}\text{x}\:}$refers to maximum power of MGT that equals to its rated power (1500 kW).

(b) **Diesel generator restrictions**.

To extend the diesel generator’s life, the power produced by diesel generator should not increase over the power limits specified in Eq. (16) that indicated as following:

16 $$\:{{\text{P}}_{\text{D}\:\text{m}\text{i}\text{n}}\le\:\text{P}}_{\text{D}}\left(\text{t}\right)\le\:{\text{P}}_{\text{D}\:\text{m}\text{a}\text{x}\:}\forall\:\:\:\text{t}\:\epsilon\:[\:1,\:24\:]$$

Where: $\:{\text{P}}_{\text{D}\:\text{m}\text{i}\text{n}}$ refers to minimum power of diesel generator of about 10% of its rated power (100 kW), and $\:{\text{P}}_{\text{D}\:\text{m}\text{a}\text{x}\:}$ refers to maximum power of diesel generator that equals to its rated power (1000 kW).

(c) **Battery restrictions**.

The battery restrictions are represented in the SoC to prevent over charging or discharging as indicated in Eq. (17) and the battery power in case of charging and discharging for one hour as indicated in Eq. (18) as following:

17 $$\:{\text{S}\text{o}\text{C}}_{\text{m}\text{i}\text{n}}\:\le\:\text{S}\text{o}\text{C}\:\left(\text{t}\right)\le\:{\text{S}\text{o}\text{C}}_{\text{m}\text{a}\text{x}}\:\:\:\:\:\:\:\forall\:\:\:\text{t}\:\epsilon\:[\:1,\:24\:]$$

18 $$\:{{\text{P}}_{\text{B}\:\text{c}\text{h}\text{a}\text{r}}\le\:\text{P}}_{\text{B}}\left(\text{t}\right)\le\:{\text{P}}_{\text{B}\:\text{d}\text{i}\text{s}\text{c}\text{h}\text{a}\text{r}\:}\:\:\:\:\:\:\:\:\:\:\forall\:\:\:\text{t}\:\epsilon\:[\:1,\:24\:]$$

Where: $\:{\text{S}\text{o}\text{C}}_{\text{m}\text{i}\text{n}}$ refers to the minimum SoC of battery of about (20%) of its capacity, $\:{\text{S}\text{o}\text{C}}_{\text{m}\text{a}\text{x}}$ refers to the maximum state of charge of about (100%) of its capacity, $\:{\text{P}}_{\text{B}\:\text{c}\text{h}\text{a}\text{r}\text{g}}$ refers to maximum power of battery during charging for one hour that equals to (-360 kW) depend on battery capacity of 1500 kWh (battery take 5 h in case of charging or discharging), and $\:{\text{P}}_{\text{B}\:\:\text{d}\text{i}\text{s}\text{c}\text{h}\text{a}\text{r}}$ refers to maximum power of battery during discharging for one hour that equals to (360 kW).

When $\:\text{S}\text{o}\text{C}$ at (t + 1) exceed $\:{\text{S}\text{o}\text{C}}_{\text{m}\text{a}\text{x}}$, the set point of battery shall be readjusted according to the next Eq. (19) to keep the battery from overcharging:

19 $$\:{\text{P}}_{\text{b}\:}=\left(\frac{{\text{S}\text{o}\text{C}}_{\text{m}\text{a}\text{x}}-\text{S}\text{o}\text{C}\left(\text{t}\right)}{{{\upeta\:}}_{\text{c}\text{h}}\:{\Delta\:}\text{t}}\right)\text{*}{\text{W}}_{\text{b}}$$

When $\:\text{S}\text{o}\text{C}$ at (t + 1) less than $\:{\text{S}\text{o}\text{C}}_{\text{m}\text{i}\text{n}}$, the set point of battery shall be readjusted according to the following Eq. (20) to keep the battery SOC within the acceptable range:

20 $$\:{\text{P}}_{\text{b}\:}=\left(\frac{{\text{S}\text{o}\text{C}}_{\text{m}\text{i}\text{n}}-\text{S}\text{o}\text{C}\left(\text{t}\right)}{{{\upeta\:}}_{\text{d}\text{c}\text{h}}\:{\Delta\:}\text{t}}\right)\text{*}{\text{W}}_{\text{b}}$$

(d) **Utility grid restrictions**.

The electricity transferred between the MGs and the utility through the connected line (P_utility_) must not exceed the authorized limitations as illustrated in Eq. (21):

21 $$\:{{\text{P}}_{\text{u}\text{t}\text{i}\text{l}\text{i}\text{t}\text{y}\:\text{m}\text{i}\text{n}}\le\:\text{P}}_{\text{u}\text{t}\text{i}\text{l}\text{i}\text{t}\text{y}}\left(\text{t}\right)\le\:{\text{P}}_{\text{u}\text{t}\text{i}\text{l}\text{i}\text{t}\text{y}\:\text{m}\text{a}\text{x}\:}\:\:\forall\:\:\:\text{t}\:\epsilon\:[\:1,\:24\:]$$

Where: $\:{\text{P}}_{\text{u}\text{t}\text{i}\text{l}\text{i}\text{t}\text{y}\:\text{m}\text{i}\text{n}}$ refers to minimum power transfers between MGs and the main utility, and $\:{\text{P}}_{\text{u}\text{t}\text{i}\text{l}\text{i}\text{t}\text{y}\:\text{m}\text{a}\text{x}\:}$refers to the maximum power transferred between MGs and the main utility grid that depend in thermal capacity of transmission line connected between MGs and main utility grid.

(e) **Power balance restrictions**.

In the case of total operational conditions, there must be a balance occurred among power generation and power demand. This can be achieved by Eq. (22) that illustrates the power balance equation during grid connected mode.

22 $$\:{\text{P}}_{\text{G}}\left(\text{t}\right)+{2\text{*}\text{P}}_{\text{B}}\left(\text{t}\right)+{2\text{*}\text{P}}_{\text{D}}\left(\text{t}\right)+{\text{P}}_{\text{F}}\left(\text{t}\right)+{\text{P}}_{\text{p}\text{v}}\left(\text{t}\right)+{\text{P}}_{\text{w}}\left(\text{t}\right)+\:{\text{P}}_{\text{u}\text{t}\text{i}\text{l}\text{i}\text{t}\text{y}}\left(\text{t}\right)={\text{P}}_{\text{L}}\left(\text{t}\right)\:\:\forall\:\:\text{t}\:\epsilon\:[\:1,\:24\:]$$

Where: $\:{\text{P}}_{\text{G}}\left(\text{t}\right)$ refers to the MGT power during the day, $\:{\text{P}}_{\text{B}}\left(\text{t}\right)$ refers to the battery power during the day, $\:{\text{P}}_{\text{F}}\left(\text{t}\right)$ refers to the fuel cell power during the day, $\:{\text{P}}_{\text{p}\text{v}}\left(\text{t}\right)$ refers to the photovoltaic power during the day, $\:{\text{P}}_{\text{w}}\left(\text{t}\right)$ is the wind power during the day, $\:{\text{P}}_{\text{u}\text{t}\text{i}\text{l}\text{i}\text{t}\text{y}}\left(\text{t}\right)$ refers to the power transferred between grid and the MGs during the day, and $\:{\text{P}}_{\text{L}}\left(\text{t}\right)$ refers to the demand power during the day.

While the power balance equation during autonomous mode can be illustrated by Eq. (23) as following:

23 $$\:{\text{P}}_{\text{G}}\left(\text{t}\right)+{2\text{*}\text{P}}_{\text{B}}\left(\text{t}\right)+{2\text{*}\text{P}}_{\text{D}}\left(\text{t}\right)+{\text{P}}_{\text{F}}\left(\text{t}\right)+{\text{P}}_{\text{p}\text{v}}\left(\text{t}\right)+{\text{P}}_{\text{w}}\left(\text{t}\right)={\text{P}}_{\text{L}}\left(\text{t}\right)\:\:\forall\:\:\text{t}\:\epsilon\:[\:1,\:24\:]$$

## One to one based optimizer (OOBO

**Fig. 2:**
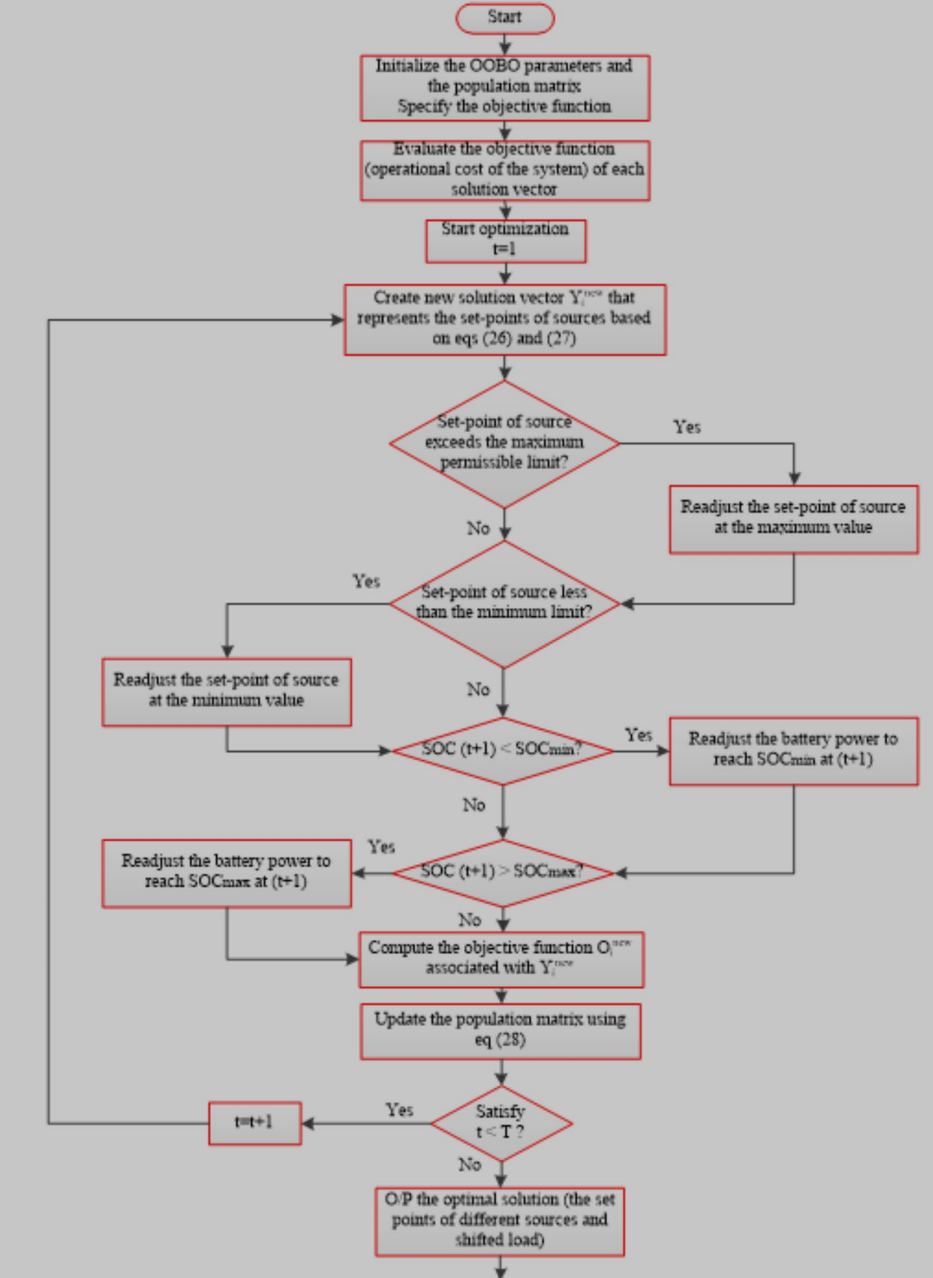
Flow chart of OOBO.

It is a novel metaheuristic technique used to solve the optimization issues in a variety of scientific fields. This technique according to^22^ outperforms in tackling optimization difficulties and can produce more acceptable and satisfied results compared with other optimization techniques. This technique is implemented in ten objective functions in two dimensions to check its ability in facing the optimization problem difficulties as indicated in^22^. In^30^ there is more information about a set of 57 single-objective and constrained benchmark problems that can be used for testing the optimization technique. The main goal in constructing the OOBO technique is to effectively employ the expertise of total members in case of updating the population of the algorithm instead of depending on certain members for the updating process. The main idea of the algorism is that a one-to-one connection among the two groups of members and the members chosen as guides to promote the participation of all members in the process of updating the population. Each population member is picked once as a guide and is only used to update another member in this one-on-one contact. The steps of the OOBO are illustrated as follows^23^. The flow chart that illustrates the steps of OOBO is indicated in Fig. 2.

Initializing the algorithm, The OOBO algorithm assigns values to decision variables based on the location of each population member in the search area, representing suggested solutions to a particular problem. OOBO represents each member as a vector given by:

24 $$\:\overrightarrow{{\text{Y}}_{\text{i}}}=\left[{\text{Y}}_{\text{i},1}+\dots\:+{\text{Y}}_{\text{i},\text{d}}+\dots\:+{\text{Y}}_{\text{i},\text{m}}\right],\:\text{i}=1,\dots\:,\text{N}.$$

To create the initial population, the population members are randomly placed in the search area using the following:

25 $$\:{\text{Y}}_{\text{i},\text{d}}={\text{l}\text{b}}_{\text{d}}+\text{r}\text{a}\text{n}\text{d}\left(\:\right).\left({\text{u}\text{b}}_{\text{d}}-{\text{l}\text{b}}_{\text{d}}\right),\:\text{d}=1,\dots\:,\text{m}.$$

Where: $\:\text{r}\text{a}\text{n}\text{d}\left(\:\right)$ is a function that produce random number between (0,1), and N is the population size.

The algorithm population is indicated by the following matrix:

26 $$\:\overrightarrow{\text{Y}}=\left[\begin{array}{c}\overrightarrow{{\text{Y}}_{1}}\\\:.\\\:\overrightarrow{\begin{array}{c}{\text{Y}}_{\text{i}}\\\:.\\\:\overrightarrow{{\text{Y}}_{\text{N}}}\end{array}}\end{array}\right]={\left[\begin{array}{c}{\text{Y}}_{\text{1,1}}\dots\:\:{\text{Y}}_{1,\text{d}}\dots\:{\text{Y}}_{1,\text{m}}\:\\\:\dots\:\:\:\:\:\:\:\:\:\:\:\:\dots\:\:\:\:\:\:\:\:\dots\:\:\:\:\\\:{\text{Y}}_{\text{i},1}\dots\:\:{\text{Y}}_{1,\text{d}}\dots\:{\text{Y}}_{1,\text{m}}\\\:\dots\:\:\:\:\:\:\:\:\:\:\:\:\dots\:\:\:\:\:\:\:\:\dots\:\:\\\:{\text{Y}}_{\text{N},1}\dots\:\:{\text{Y}}_{\text{N},\text{d}}\dots\:{\text{Y}}_{\text{N},\text{m}}\end{array}\right]}_{\text{N}\times\:\text{m}}$$

Then the objective function of the optimized problem can be expressed by following equation:

27 $$\:\overrightarrow{\text{O}}={\left[\begin{array}{c}{\text{O}}_{1}\\\:.\\\:{\text{O}}_{\text{i}}\\\:.\\\:{\text{O}}_{\text{N}}\end{array}\right]}_{\text{N}\times\:1}={\left[\begin{array}{c}O\left(\overrightarrow{{\text{Y}}_{1}}\right)\\\:.\\\:O\left(\overrightarrow{{\text{Y}}_{\text{i}}}\right)\\\:.\\\:O\left(\overrightarrow{{\text{Y}}_{\text{N}}}\right)\end{array}\right]}_{\text{N}\times\:1}$$

Where: $\:\overrightarrow{\text{O}}$, and $\:{\text{O}}_{\text{i}}$ are the vector of the objective function, and the proposed solution objective function respectively.

The process of estimating the new state of populations in the search area is depicted using the following equation:

28 $$\:{\text{Y}}_{\text{i},\text{d}}^{\text{n}\text{e}\text{w}}=\left\{\begin{array}{c}{\text{Y}}_{\text{i},\text{d}}+ran\:\left(\:\right).\left({\text{Y}}_{\text{k}\text{i},\text{d}}-\text{A}\:{\text{Y}}_{\text{i},\text{d}}\right)\:{\:\:\:\:\:\:\:\:\:\text{O}}_{\text{k}\text{i}}<\:{\text{O}}_{\text{i}}\\\:{\text{Y}}_{\text{i},\text{d}}+ran\:\left(\:\right).\left({\text{Y}}_{\text{i},\text{d}}-\:{\text{Y}}_{\text{k}\text{i},\text{d}}\right)\:{\:\:\:\:\:\:\:\:\:\text{O}}_{\text{k}\text{i}}>\:{\text{O}}_{\text{i}}\:\:\end{array}\right.\:$$

29 $$\:\text{K}=\:\text{r}\text{o}\text{u}\text{n}\text{d}\:(\:1+\text{r}\text{a}\text{n}\left(\:\right))$$

Where: $\:{\text{Y}}_{\text{i},\text{d}}$ is the old position of the member, $\:{\text{Y}}_{\text{k}\text{i},\text{d}}$ is randomly selected member in dimension (d), A is a random number between (1, 2), ran () is a random number between (0, 1), $\:{\text{Y}}_{\text{i},\text{d}}^{\text{n}\text{e}\text{w}}$ is the new proposed state, and $\:{\:\text{O}}_{\text{k}\text{i}}$ is the value of the objective function of the chosen member.

The proposed algorithm’s population update procedure ensures that the proposed new state for a member is acceptable if it improves the value of the objective function. Otherwise, the proposed new state is rejected, and as a result, the members remain in their former position.

30 $$\:{\text{Y}}_{\text{i}}=\left\{\begin{array}{c}{\text{Y}}_{\text{i}}^{\text{n}\text{e}\text{w}}\:\:\:\:\:\:\:\:\:{\text{O}}_{\text{i}}^{\text{n}\text{e}\text{w}}<\:{\text{O}}_{\text{i}}\\\:{\text{Y}}_{\text{i}}\:\:\:\:\:\:\:\:\:\:\:\:\:{\text{O}}_{\text{i}}^{\text{n}\text{e}\text{w}}>\:{\text{O}}_{\text{i}}\:\:\end{array}\right.\:$$

Where: $\:{\text{Y}}_{\text{i}}^{\text{n}\text{e}\text{w}}$ is the new proposed state in the search area, and $\:{\text{O}}_{\text{i}}^{\text{n}\text{e}\text{w}}$ is its objective function value. The optimization process is repeated until the stopping condition is satisfied (stalling, reach the maximum number of iterations T) (maximum number of iterations (T) = 10000, population size (search agent) = 50, lower boundary of battery = -360 kW, and upper boundary of battery = 360 kW).

##  Simulation results

The results obtained can be classified into four sections two sections for grid connected mode results with and without load management and the others for autonomous mode results with and without load management as indicated below:

### Grid connected mode results before load management

**Fig. 3:**
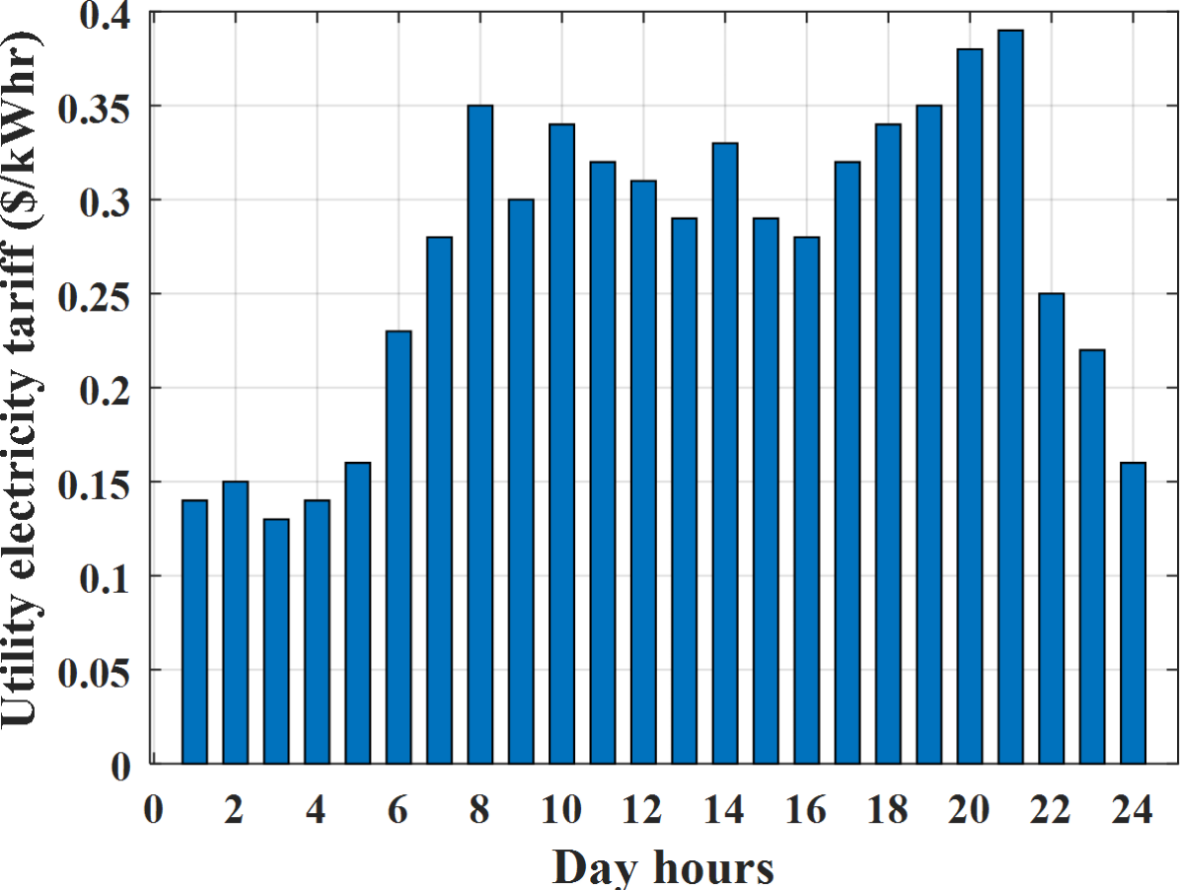
Utility electricity tariff per day.

**Fig. 4:**
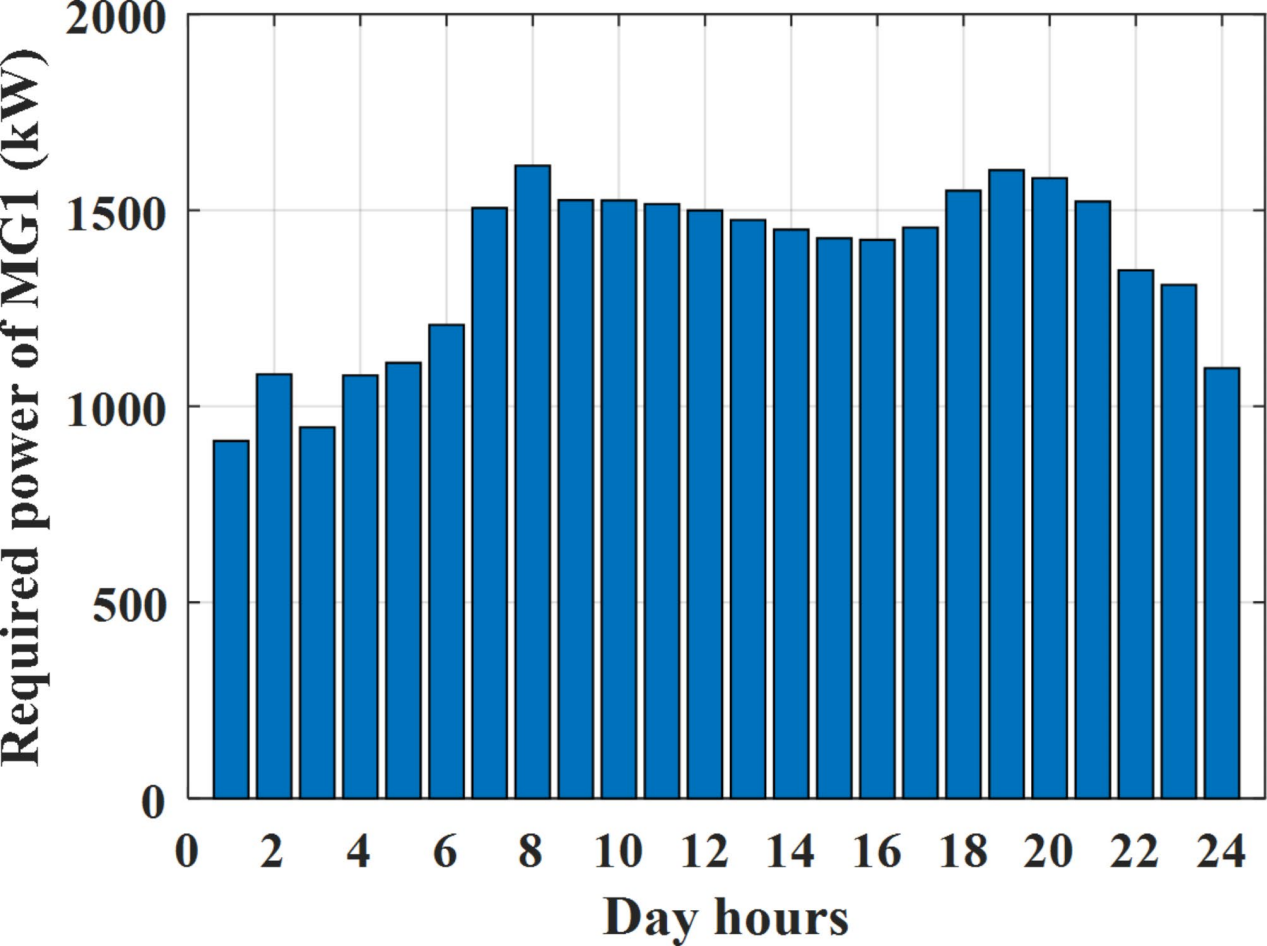
Required power for loads of MG1 before load management.

**Fig. 5:**
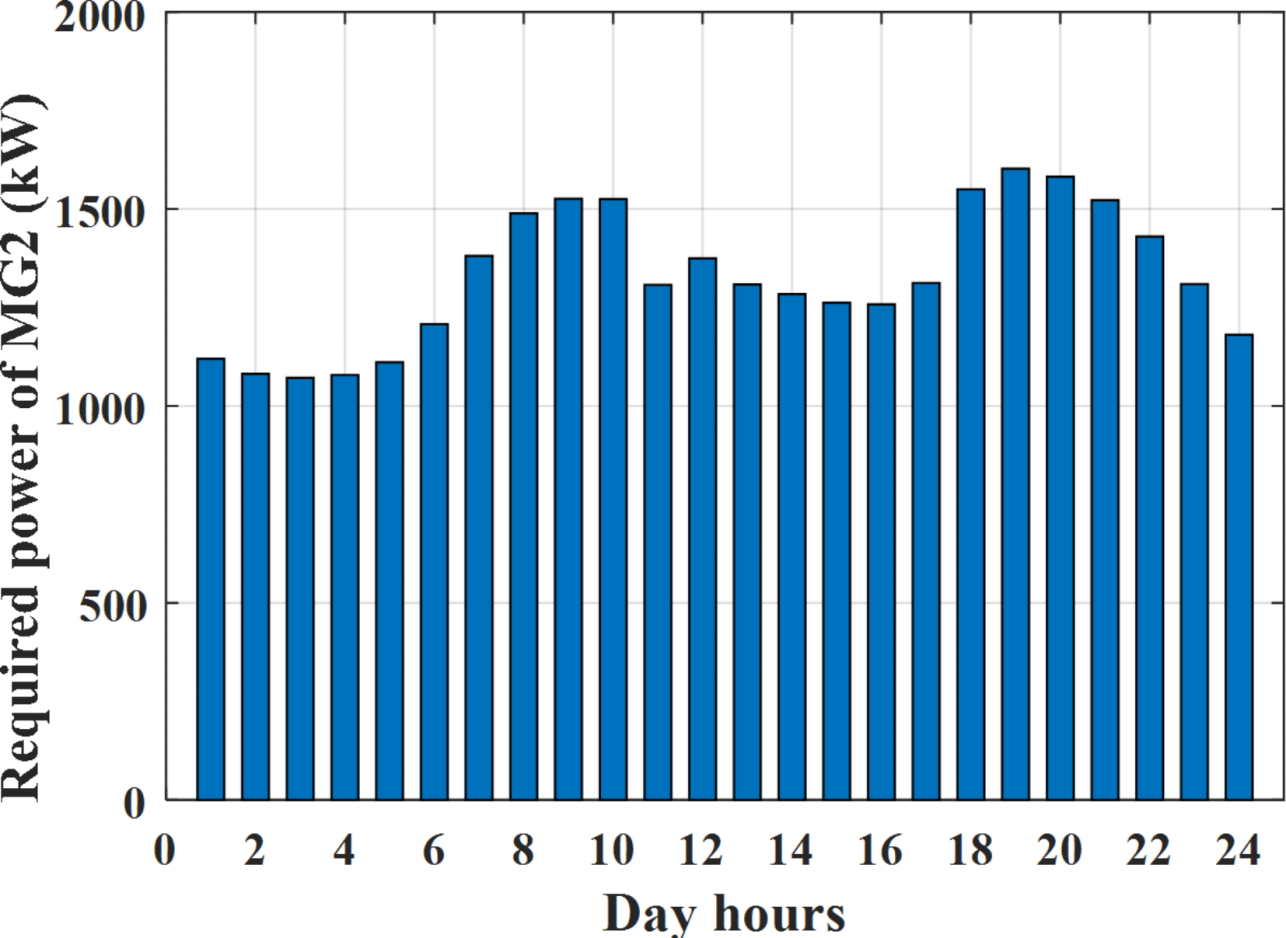
Required power for loads of MG2 before load management.

**Fig. 6:**
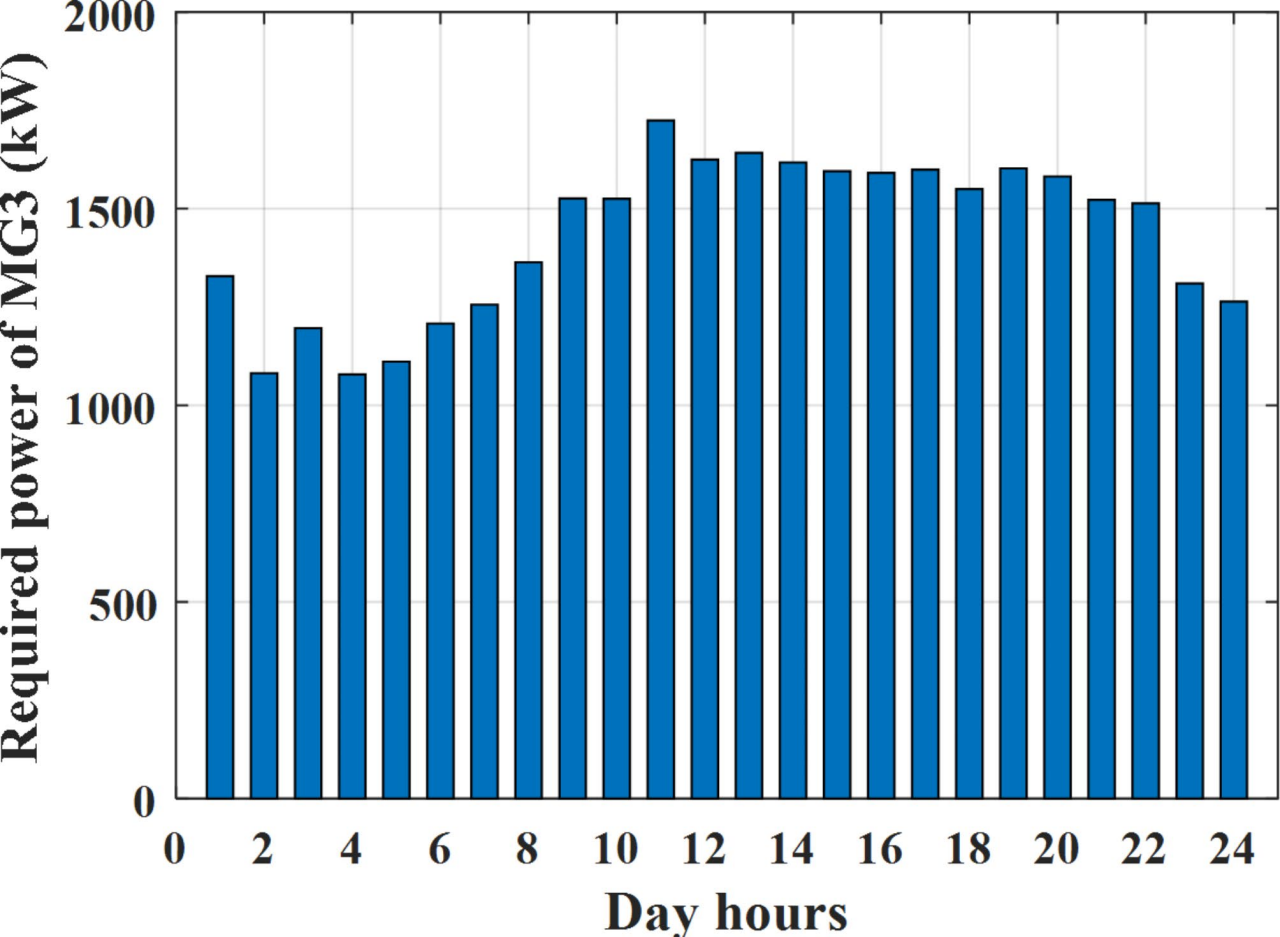
Required power for loads of MG3 before load management.

**Fig. 7:**
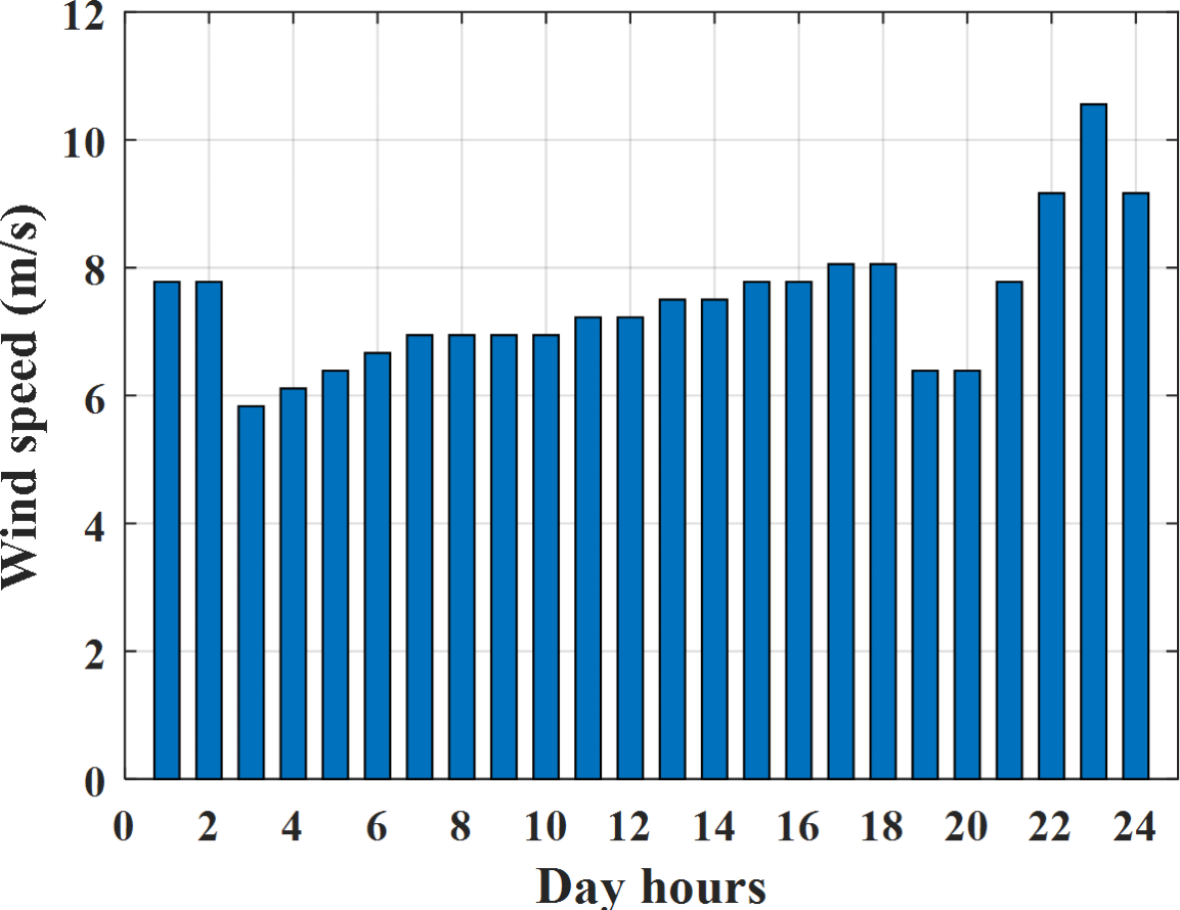
Wind speed all over the day.

**Fig. 8:**
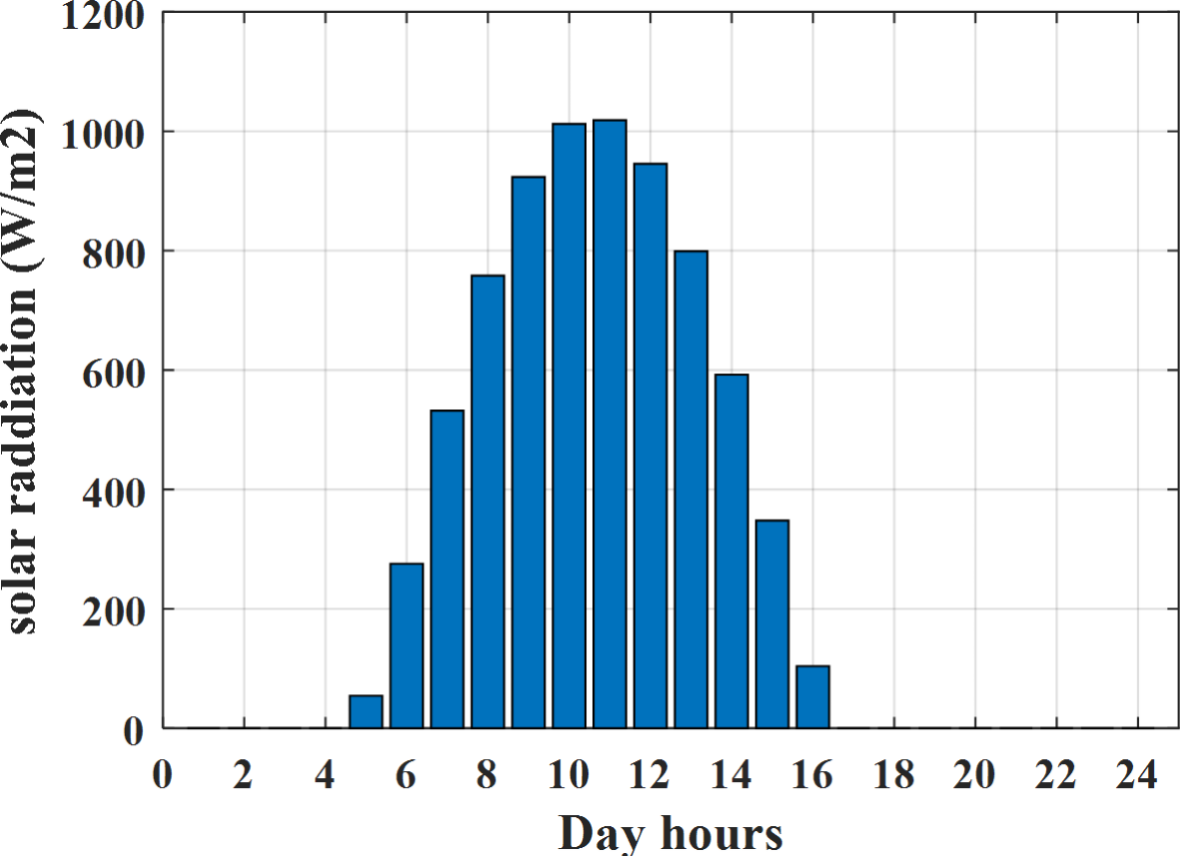
Solar irradiance all over the day.

**Fig. 9:**
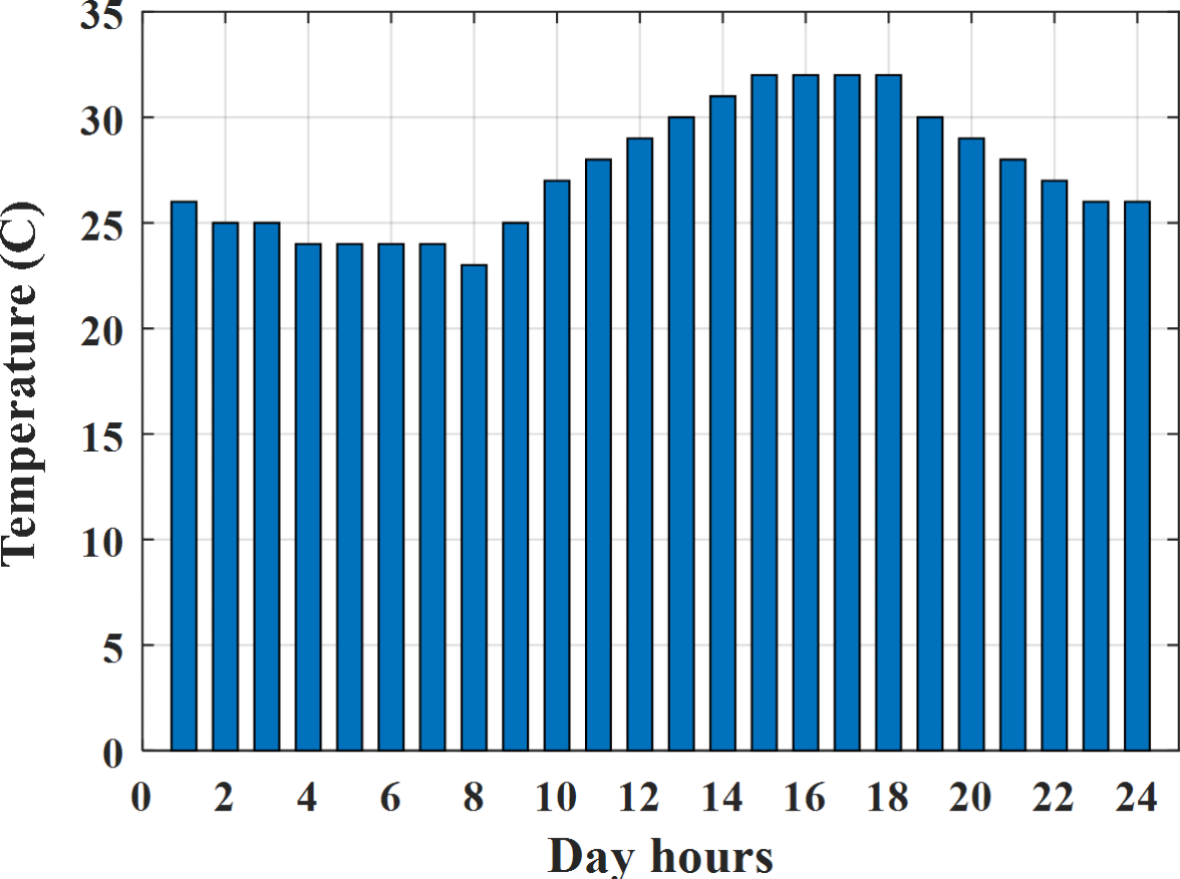
Temperature all over the day.

**Fig. 10:**
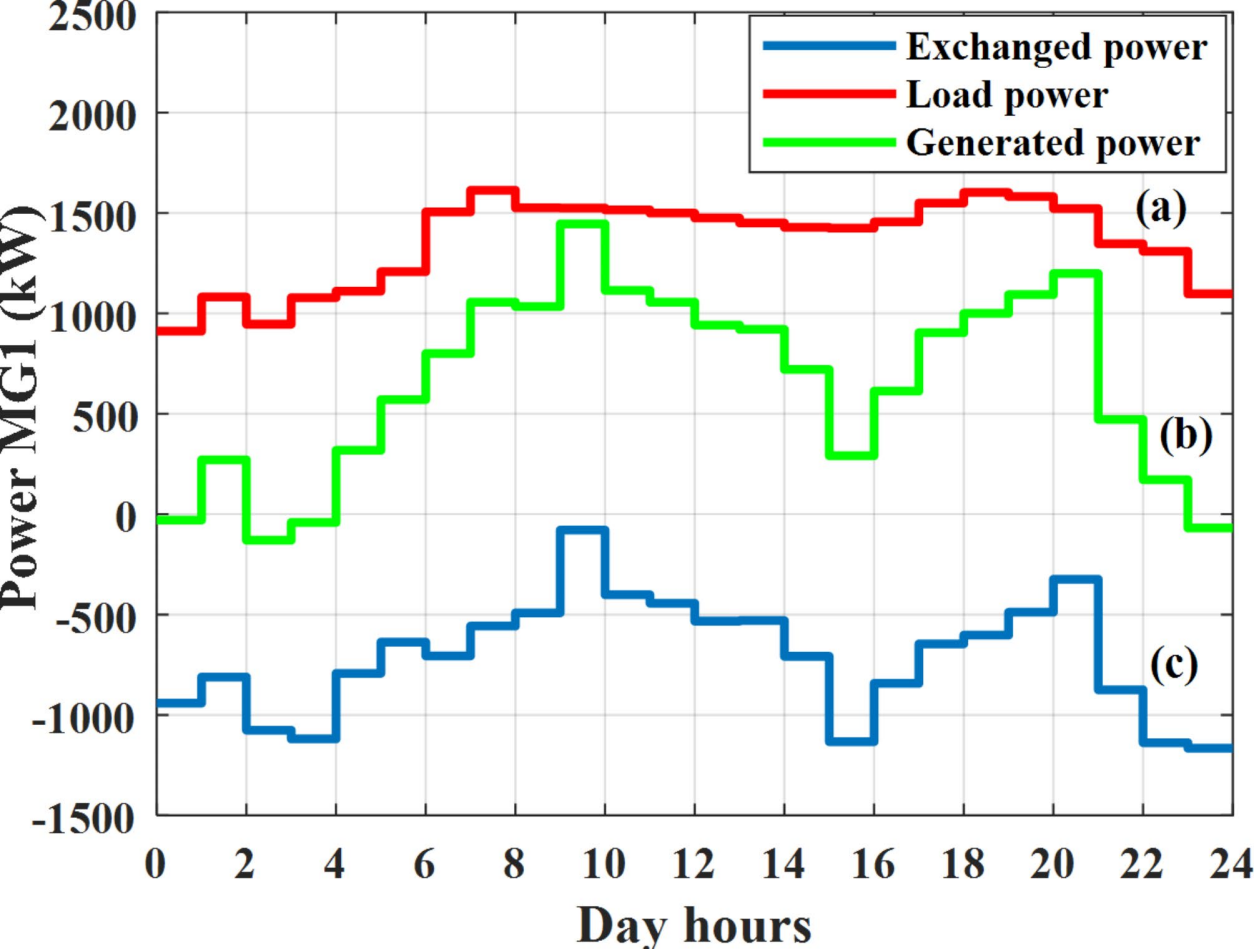
Power of MG1 all over the day during grid connected mode before load management.

**Fig. 11:**
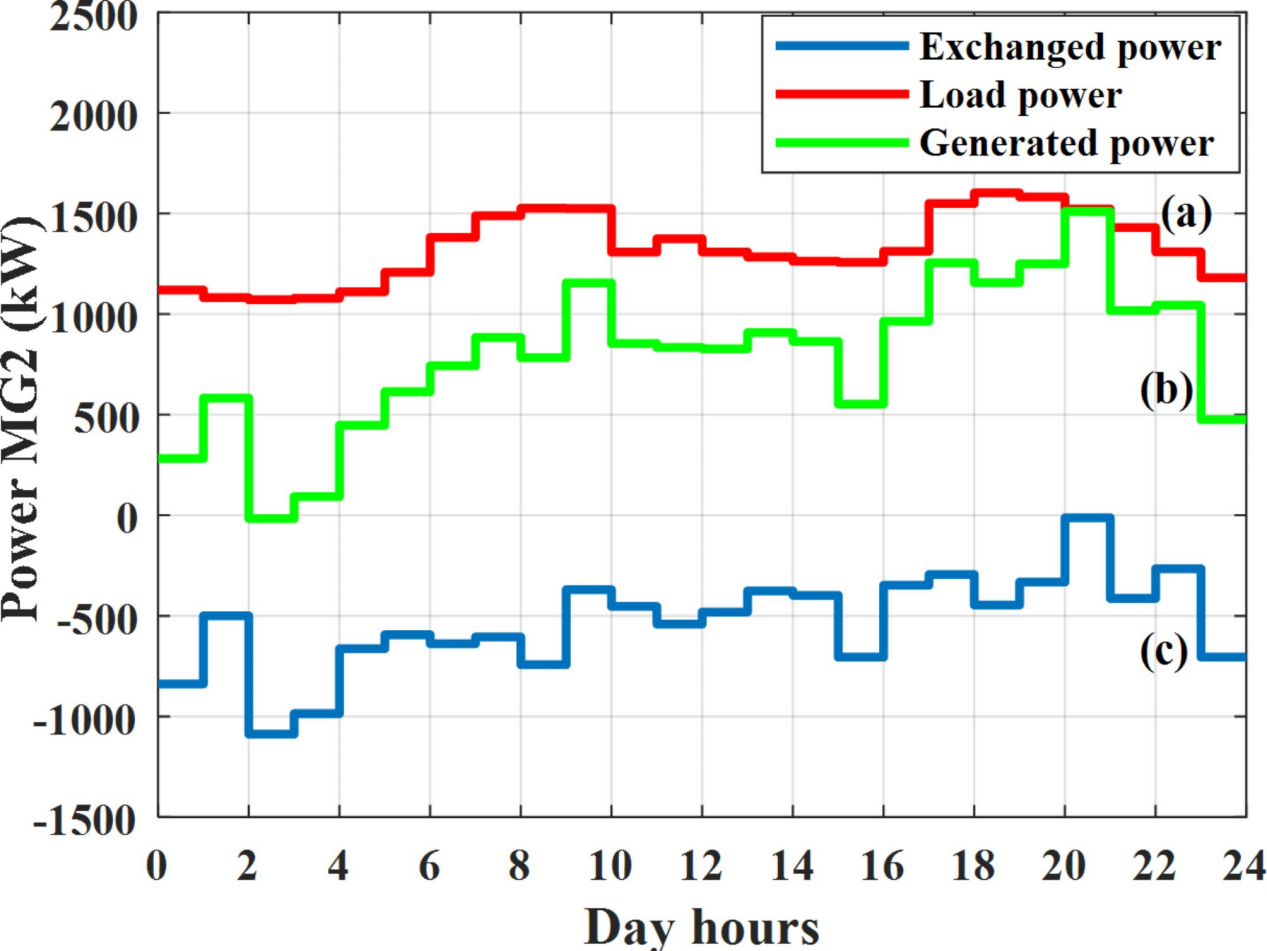
Power of MG2 all over the day during grid connected mode before load management.

**Fig. 12:**
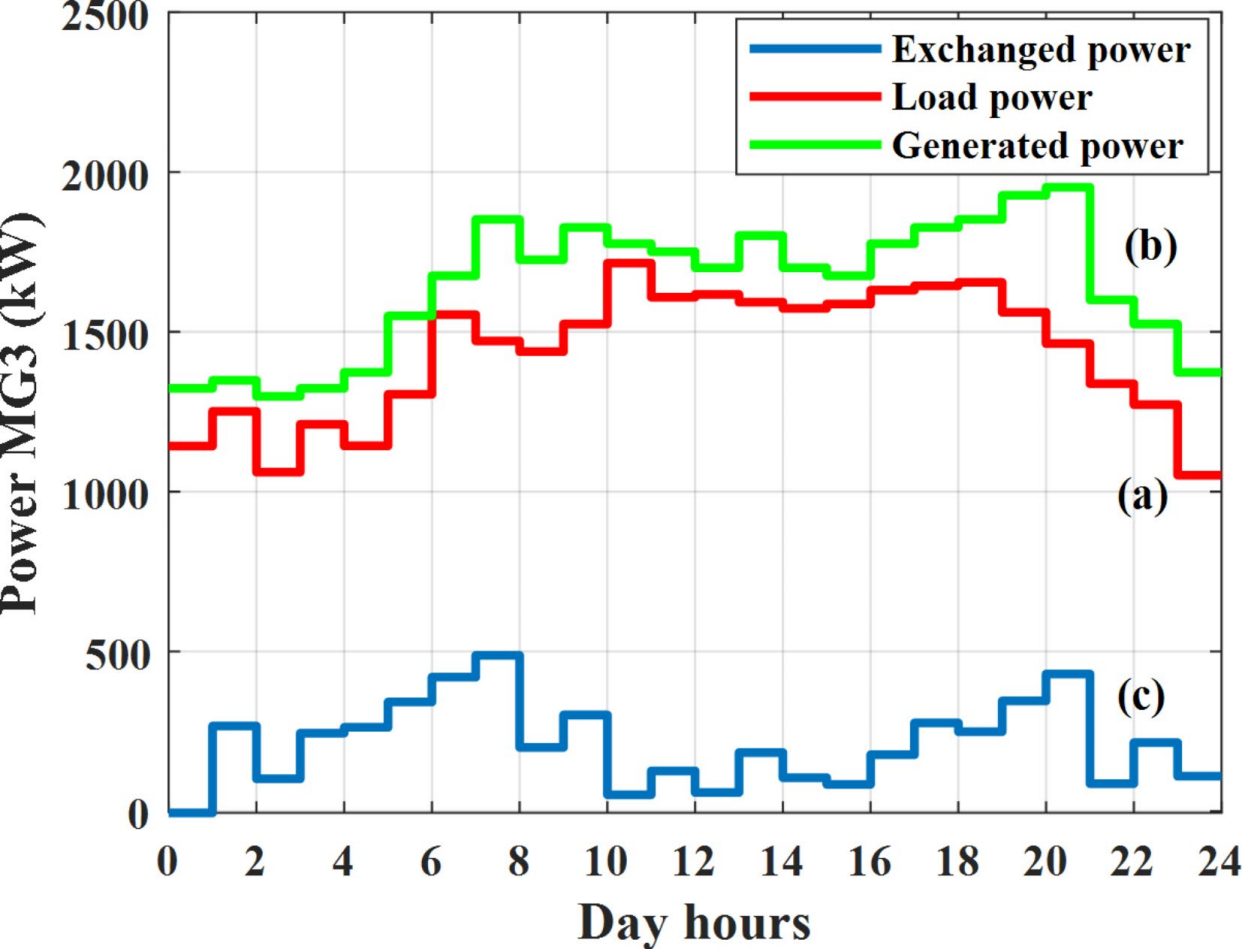
Power of MG3 all over the day during grid connected mode before load management.

Depending on the previous data obtained, the electricity tariff price of the utility during the day hours is illustrated as indicated in Fig. 3. While the total required power for loads during the day hours for MG1, MG2, and MG3 is indicated in Figs. 4, 5, and 6 consequently. Though the wind velocity and solar irradiance during the day hours are indicated in Figs. 7 and 8  consequently. Figure 9 illustrates the day hours ambient temperatures. The total power flow through the MG1 is indicated in Fig. 10 that illustrates the required power for loads that represented by Fig. 10a while the generated power from sources at MG1 is indicated by Fig. 10b. The difference between generated power and load power is the power exchanged from MG1 to other MGs and grid or power exchanged from MGs and grid to MG1 as indicated in Fig. 10c. The total power flow through the MG2 is indicated in Fig. 11 that illustrates the required power for loads that represented by Fig. 11a while the generated power from sources at MG2 is indicated by Fig. 11b. The difference between generated power and load power is the power exchanged from MG2 to other MGs and grid or power exchanged from MGs and grid to MG2 as indicated in Fig. 11c. The total power flow through the MG3 is indicated in Fig. 12 that illustrates the required power for loads that represented by Fig. 12a while the generated power from sources at MG3 is indicated by Fig. 12b. The difference between generated power and load power is the power exchanged from MG3 to other MGs and grid or power exchanged from MGs and grid to MG3 as indicated in Fig. 12c.

**Fig. 13:**
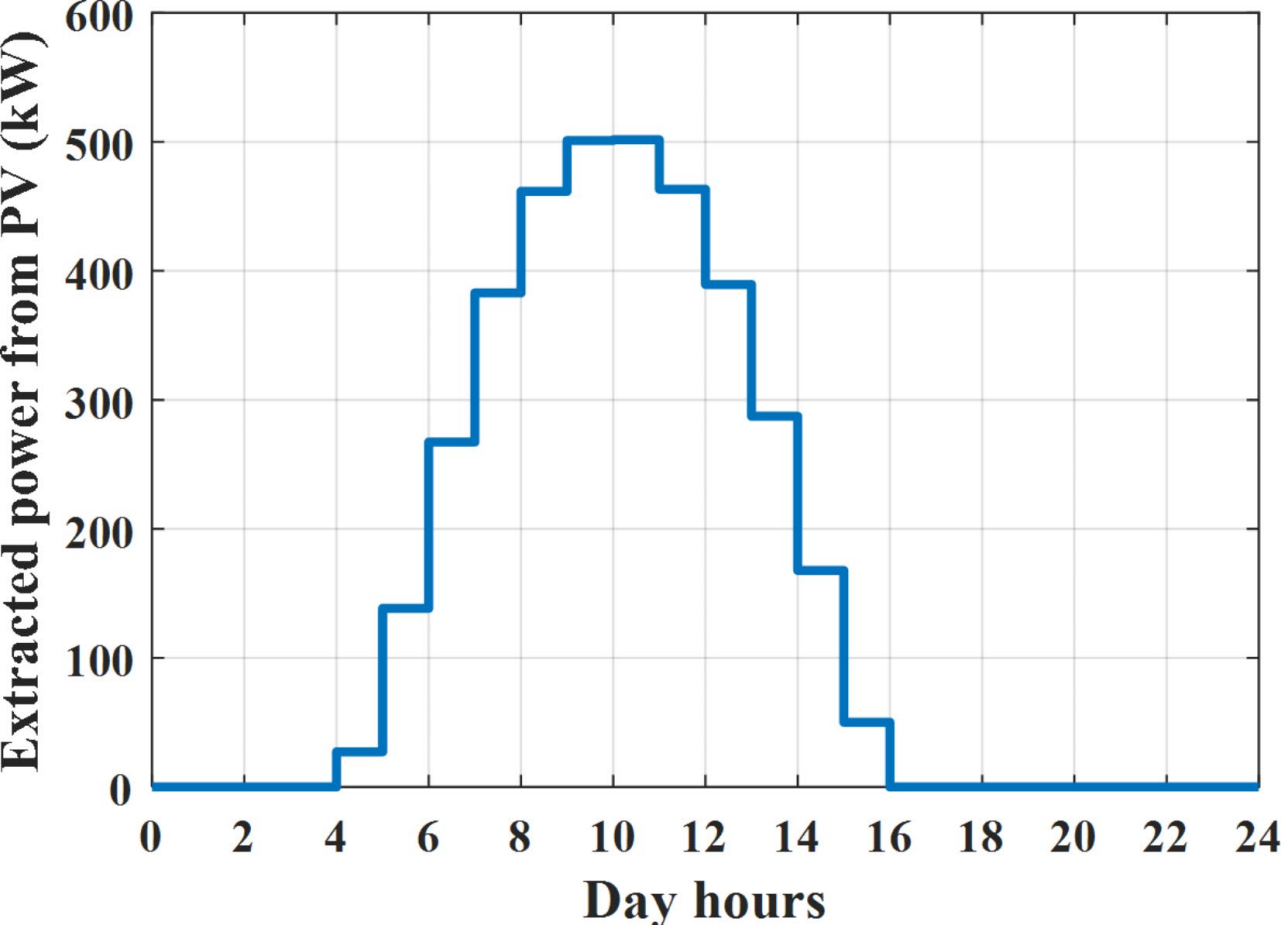
Extracted power from PV all over the day.

**Fig. 14:**
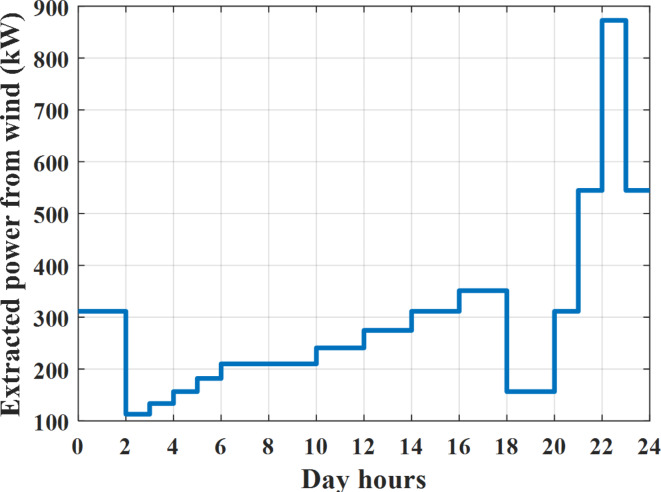
Extracted power from Wind all over the day.

**Fig. 15:**
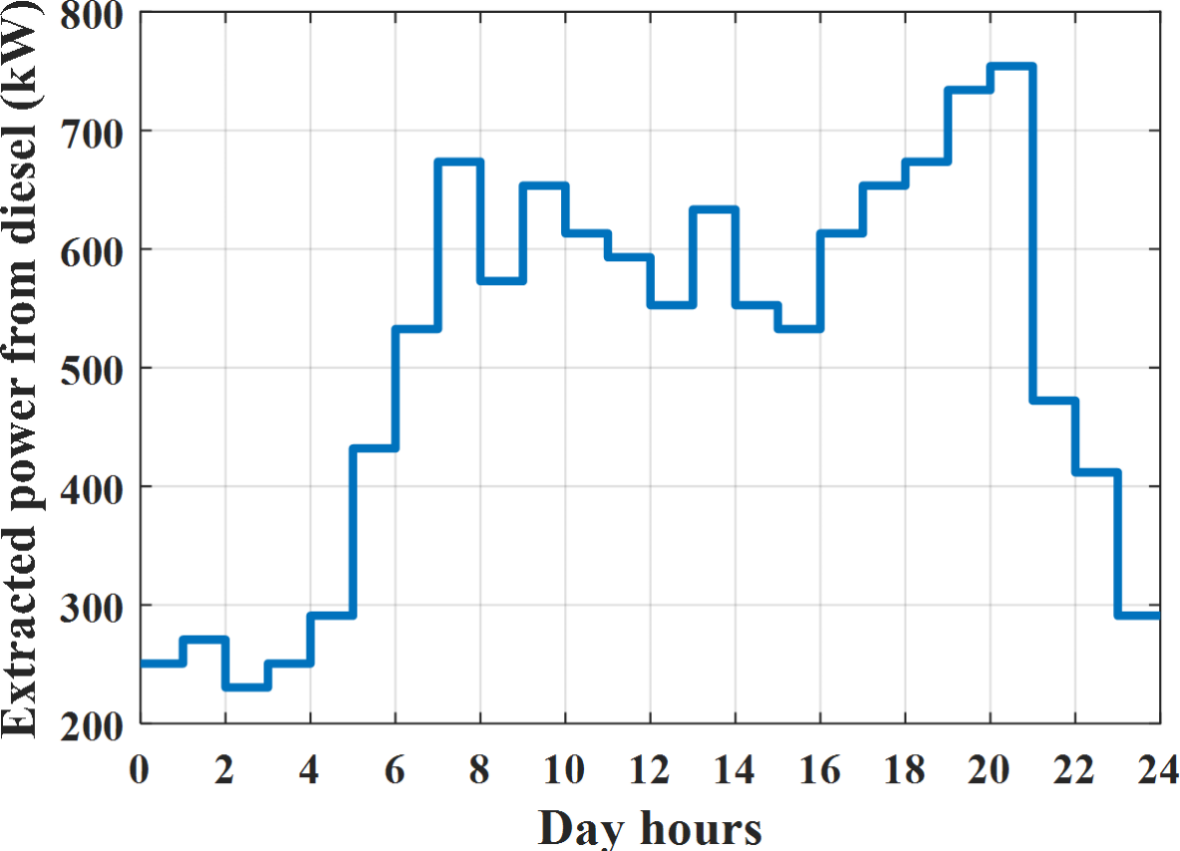
Extracted power from diesel all over the day during grid connected mode before load management.

**Fig. 16:**
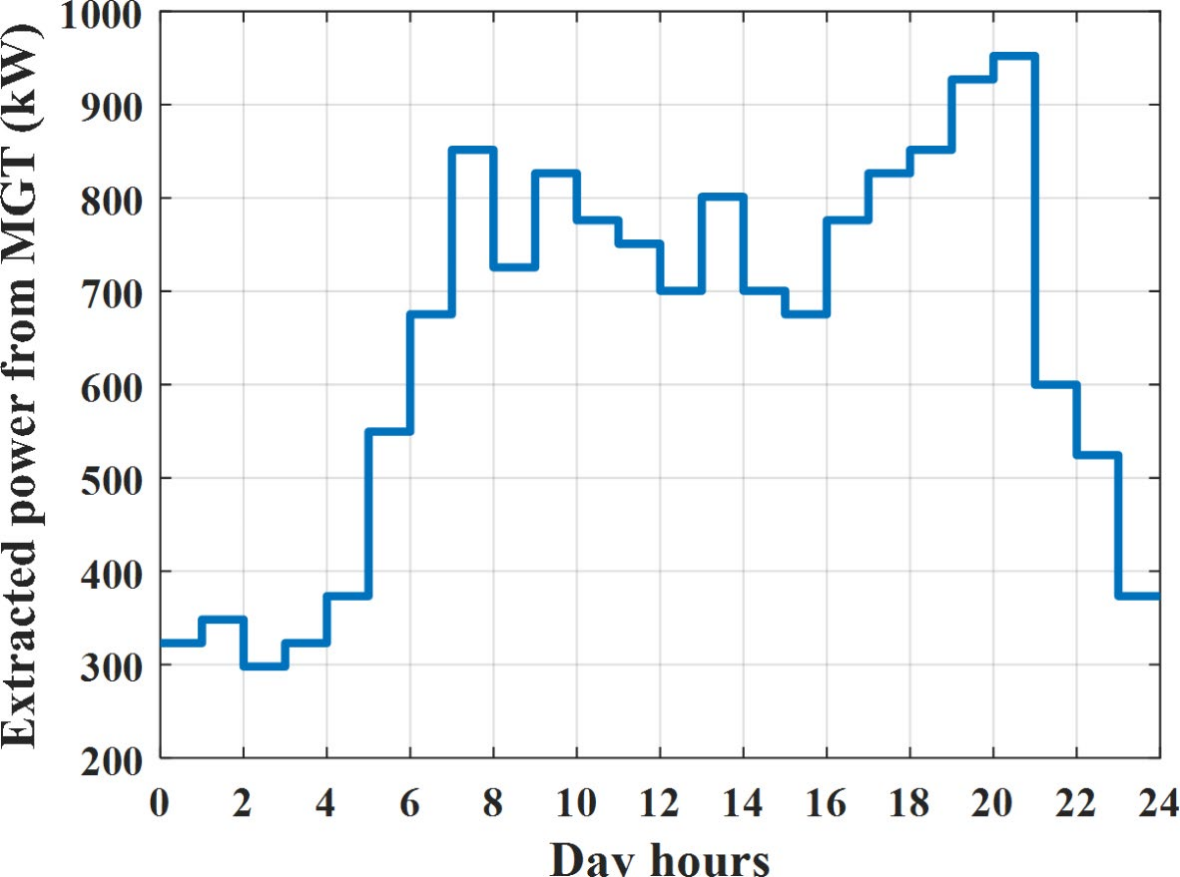
Extracted power from MGT all over the day during grid connected mode before load management.

**Fig. 17:**
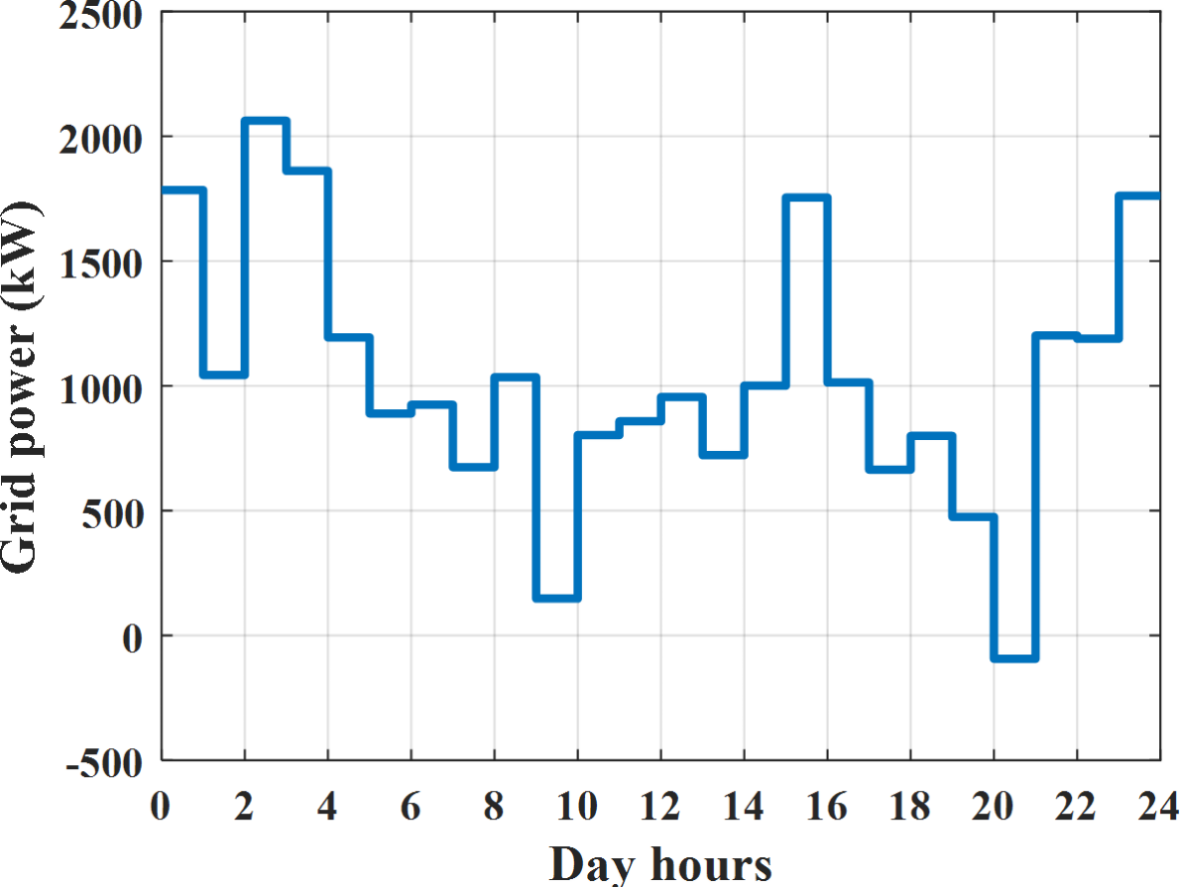
Utility power all over the day during grid connected mode before load management.

**Fig. 18:**
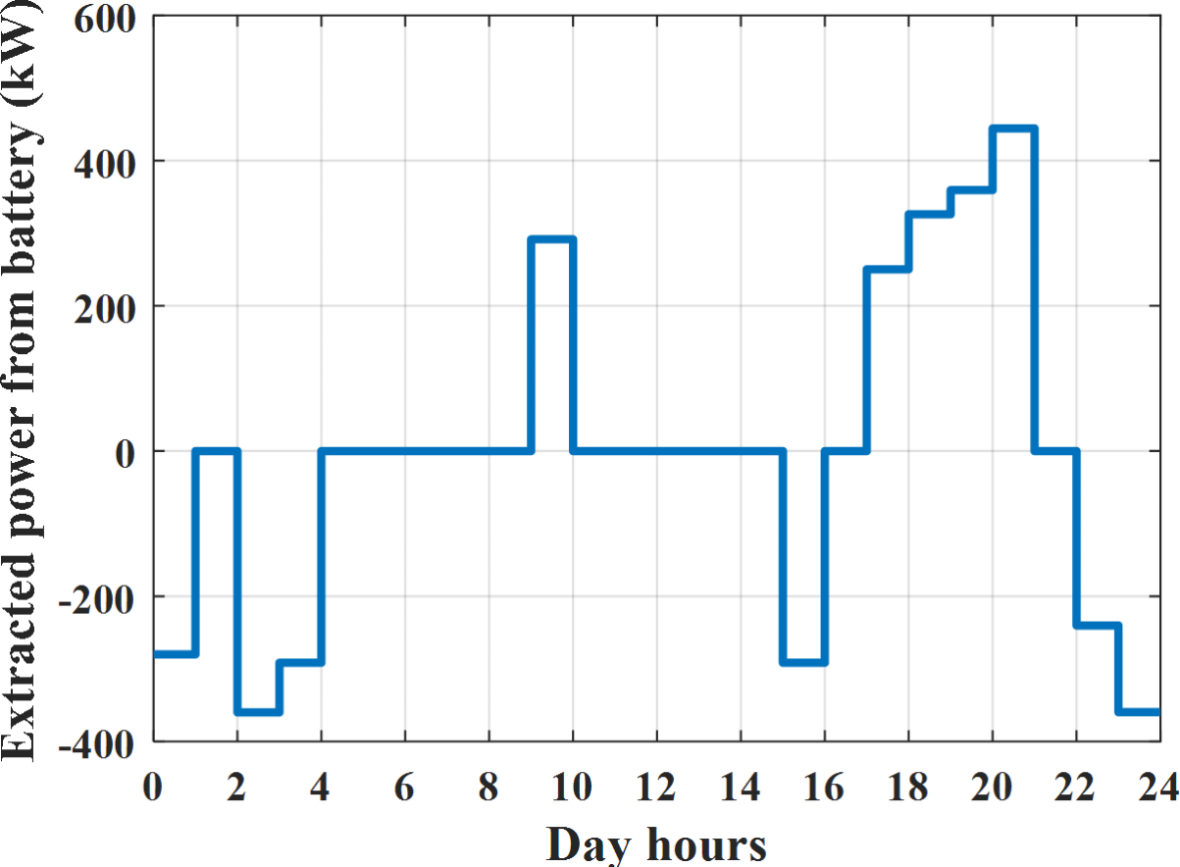
Extracted power from battery all over the day during grid connected mode before load management.

**Fig. 19:**
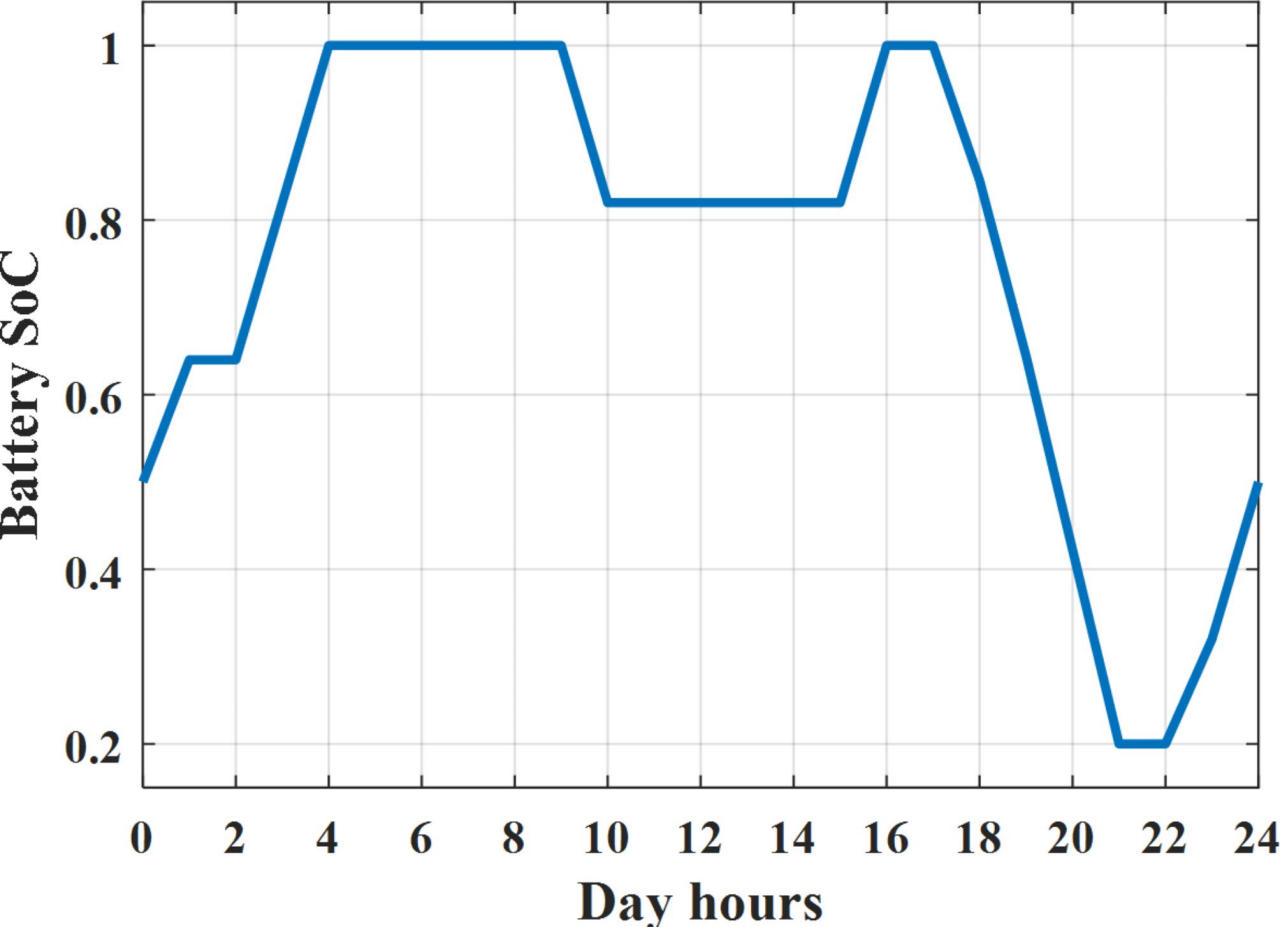
Percentage SoC of battery all over the day during grid connected mode before load management.

Depending on solar irradiance and ambient temperature appeared in Figs. 8  and 9, the extracted power from PV is obtained and illustrated by Fig. 13. From Fig. 13 it is cleared that the minimum extracted power from PV is about 27.5 kW per hour (4–5) am before this time there is no power extracted from PV and the maximum extracted power from PV is 500 kW per hour (10–11) am. After 4 pm there is no extracted power from PV, this means that the extracted power from PV is maintained for about 10 h from total day hours. Depending on wind velocity appeared in Fig. 7, the extracted power from wind turbine is illustrated by Fig. 14. From Fig. 14 it is cleared that the minimum extracted power from wind turbine is about 113 kW per hour (2–3) am and the maximum power extracted from wind turbine is about 873 kW per hour (10–11) pm. The extracted power from diesel generator during the day hours is indicated by Fig. 15. From Fig. 15 it is cleared that the minimum extracted power from diesel is about 231 kW per hour (2–3) am and maximum power extracted from diesel generator is about 755 kW per hour (10–11) pm. The extracted power from MGT generator during the day hours is indicated by Fig. 16. From Fig. 16 it is cleared that the minimum extracted power from MGT is about 298 kW per hour of (2–3) am and maximum power extracted from MGT is about 952 kW per hour (10–11) pm. The power utilized from utility during the day hours is indicated by Fig. 17. From Fig. 17 it is cleared that during all day hours there is a power taken from the grid except (10–11) pm there is a power of 94 kW is transferred from all MGs to utility (Fig. 18). The maximum power taken from grid is about 2063 kW per hour (2–3) am while minimum power taken from grid is about 148 kW per hour (9–10) am. The battery SoC is indicated in Fig. 19 while the total power extracted from battery is indicated in Fig. 18. From Fig. 18 it is cleared that the maximum power extracted from battery is about 445 kW per hour (10–11) pm.

To check the power balance between total generation and total required during the day hours the following examples for random hours will be dedicated. For example, per hour 8 am the total required load is about 4466 kW as indicated by Figs. 4, 5 and 6, so the total power from different sources should satisfy the power required for load. In this case (8 am) it is found that the power extracted from PV is about 383 kW as indicated in Fig. 13, the power extracted from wind is about 210 kW as indicated in Fig. 14, the power extracted from one diesel generator is about 674 as indicated in Fig. 15 as mentioned above in the proposed model there are two diesel generators so the total power from two diesel generators are 1348 kW, the power extracted from MGT is about 851 kW as indicated in Fig. 16, the power extracted from fuel cell is about 1000 kW, the rest of required power is taken from grid of about 674 kW as indicated in Fig. 17, and the power extracted from battery is about 0 kW as indicated in Fig. 17 (idle mode). The battery initial SoC is about 50% at 1 am (at the beginning of the day) and returns to its initial value at 12 pm (at the end of the day) as indicated in Fig. 19.

Another example to check power balance between generation and total demand during the day hours, per hour 4 pm the total required load is about 4274 kW as indicated by Figs. 4, 5 and 6, so the total power from different sources should satisfy the power required for load. In this case (4 pm) it is found that the power extracted from PV is about 50 kW as indicated in Fig. 13, the power extracted from wind is about 311 kW as indicated in Fig. 14, the power extracted from one diesel generator is about 533 as indicated in Fig. 15 as mentioned above in the proposed model there are two diesel generators so the total power from two diesel generators are 1066 kW, the power extracted from MGT is about 675 kW as indicated in Fig. 16, the power extracted from fuel cell is about 1000 kW, the total power taken from grid in this case is about 1754 as indicated in Fig. 17, there is a power of about 584 kW more than demand so this power will absorbed by two batteries each one with about 292 kW (charging mode) as indicated in Fig. 18. The battery initial SoC is about 50% at 1 am (at the beginning of the day) and return to its initial value at 12 pm (at the end of the day) as indicated in Fig. 19.

### Autonomous mode results before load management

**Fig. 20:**
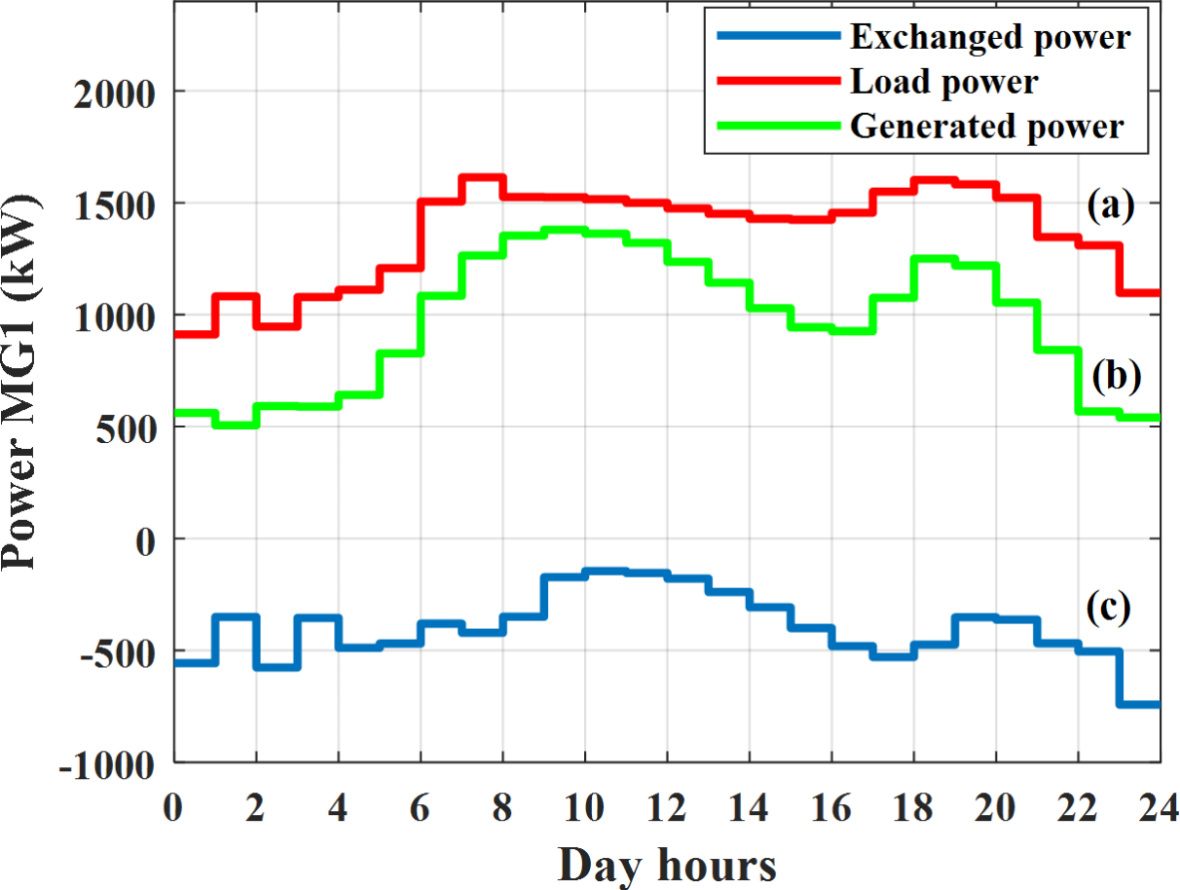
Power of MG1 all over the day during autonomous mode before load management.

**Fig. 21:**
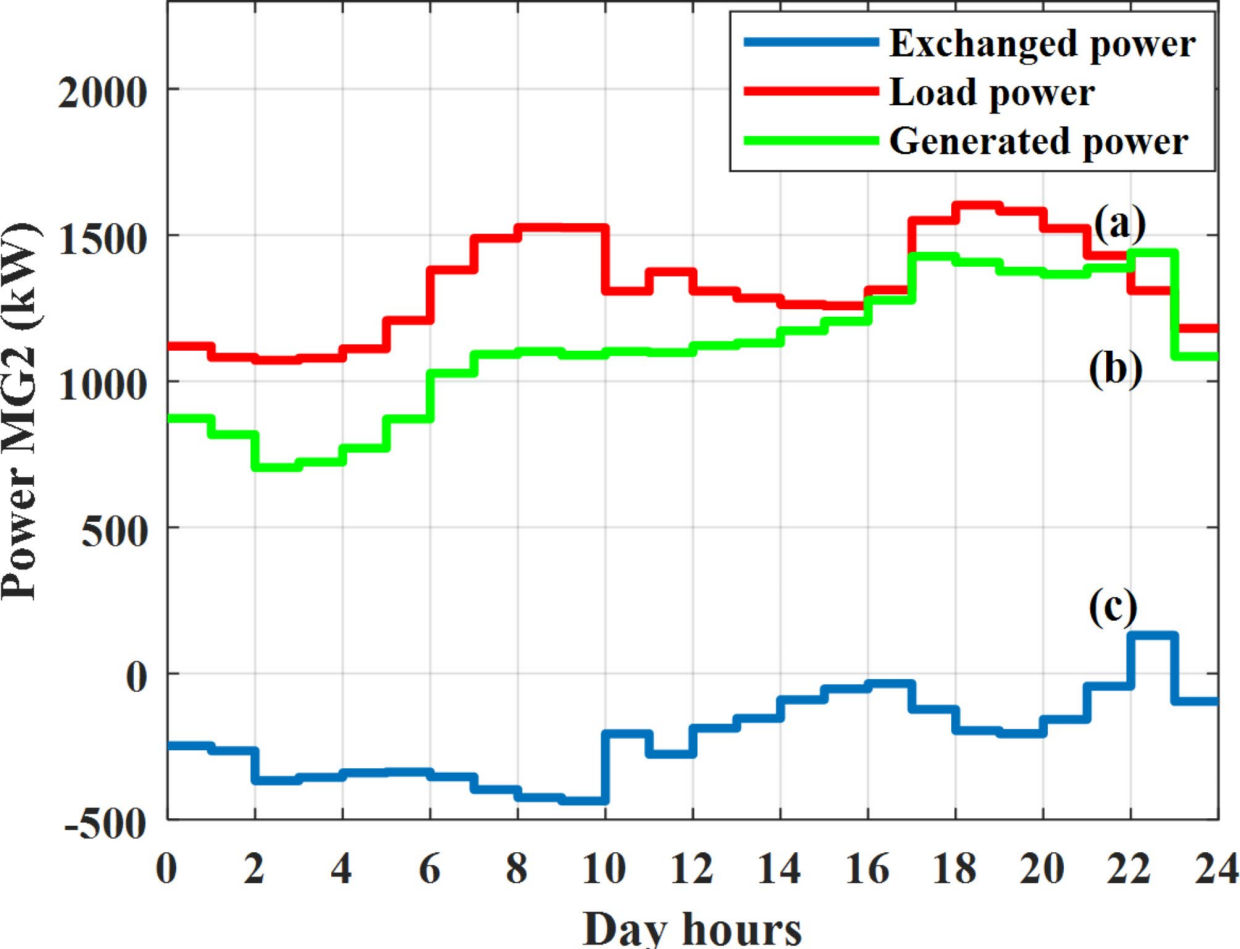
Power of MG2 all over the day during autonomous mode before load management.

**Fig. 22:**
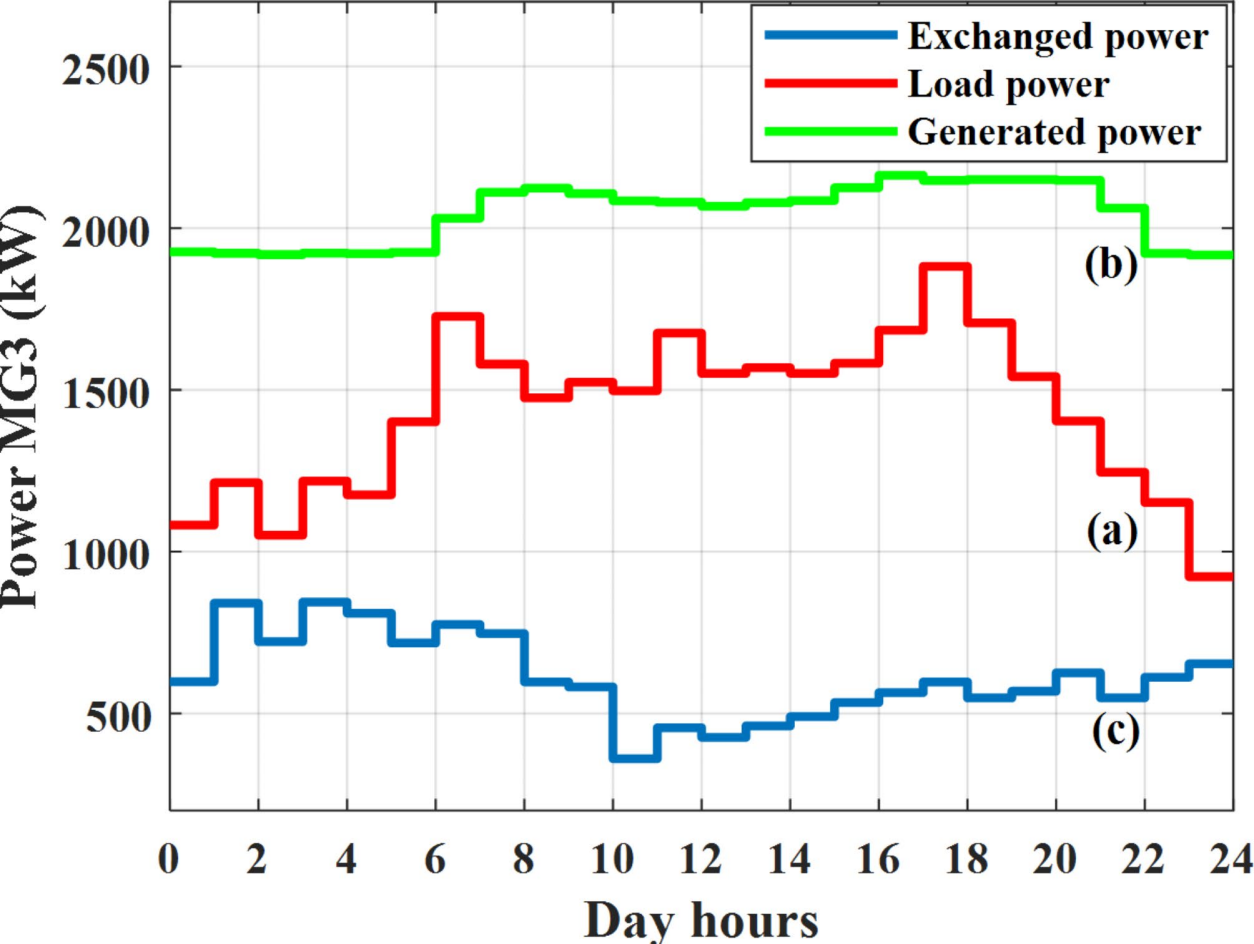
Power of MG3 all over the day during autonomous mode before load management.

In this case the MGs are disconnected from the main utility grid and operate in autonomous mode. The total power flow through the MG1 is indicated in Fig. 20 that illustrates the required power for loads that represented by Fig. 20a while the generated power from sources at MG1 is indicated by Fig. 20b. The difference between generated power and load power is the power exchanged from MG1 to other MGs or power exchanged from MGs to MG1 as indicated in Fig. 20c. The total power flow through the MG2 is indicated in Fig. 21 that illustrates the required power for loads that represented by Fig. 21a while the generated power from sources at MG2 is indicated by Fig. 21b. The difference between generated power and load power is the power exchanged from MG2 to other MGs or power exchanged from MGs to MG2 as indicated in Fig. 21c. The total power flow through the MG3 is indicated in Fig. 22 that illustrates the required power for loads that represented by Fig. 22a while the generated power from sources at MG3 is indicated by Fig. 22b. The difference between generated power and load power is the power exchanged from MG3 to other MGs or power exchanged from MGs to MG3 as indicated in Fig. 22c.

**Fig. 23:**
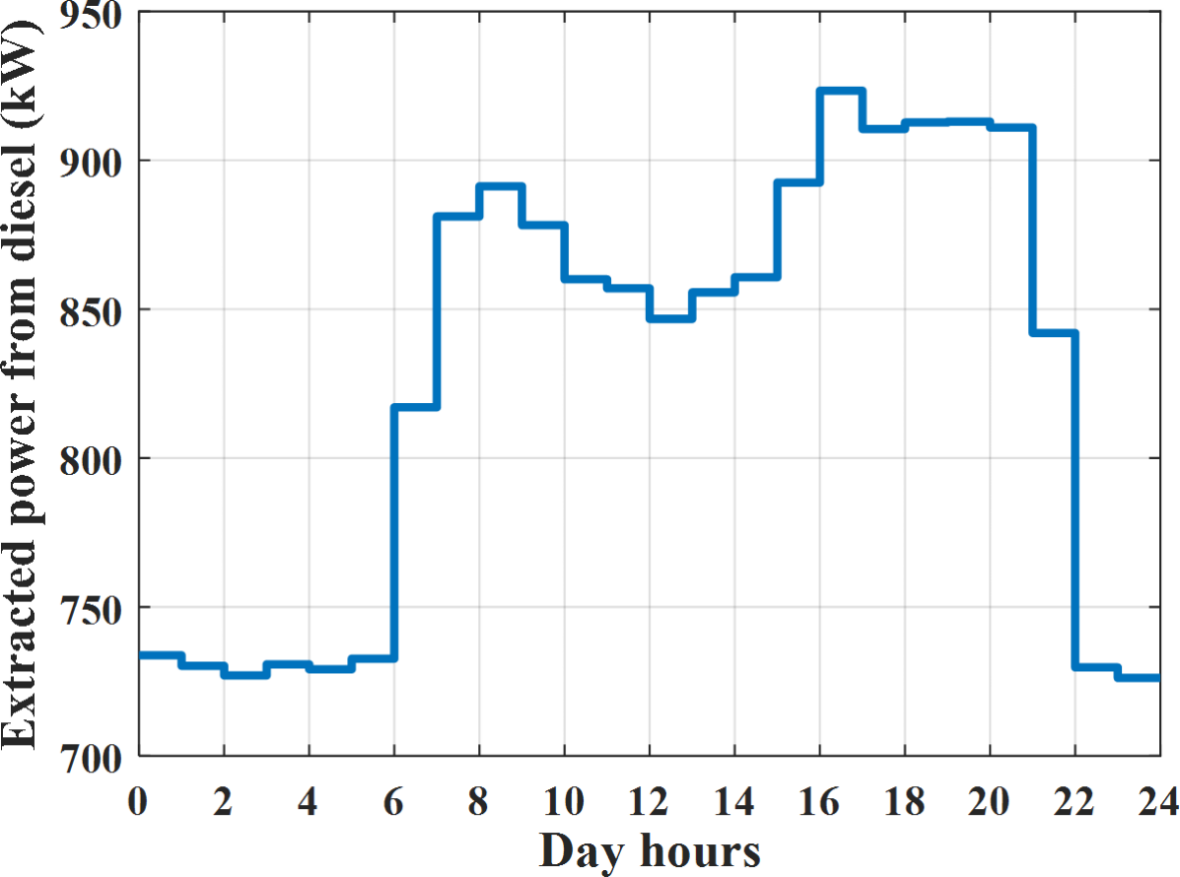
Extracted power from diesel all over the day during autonomous mode before load management.

**Fig. 24:**
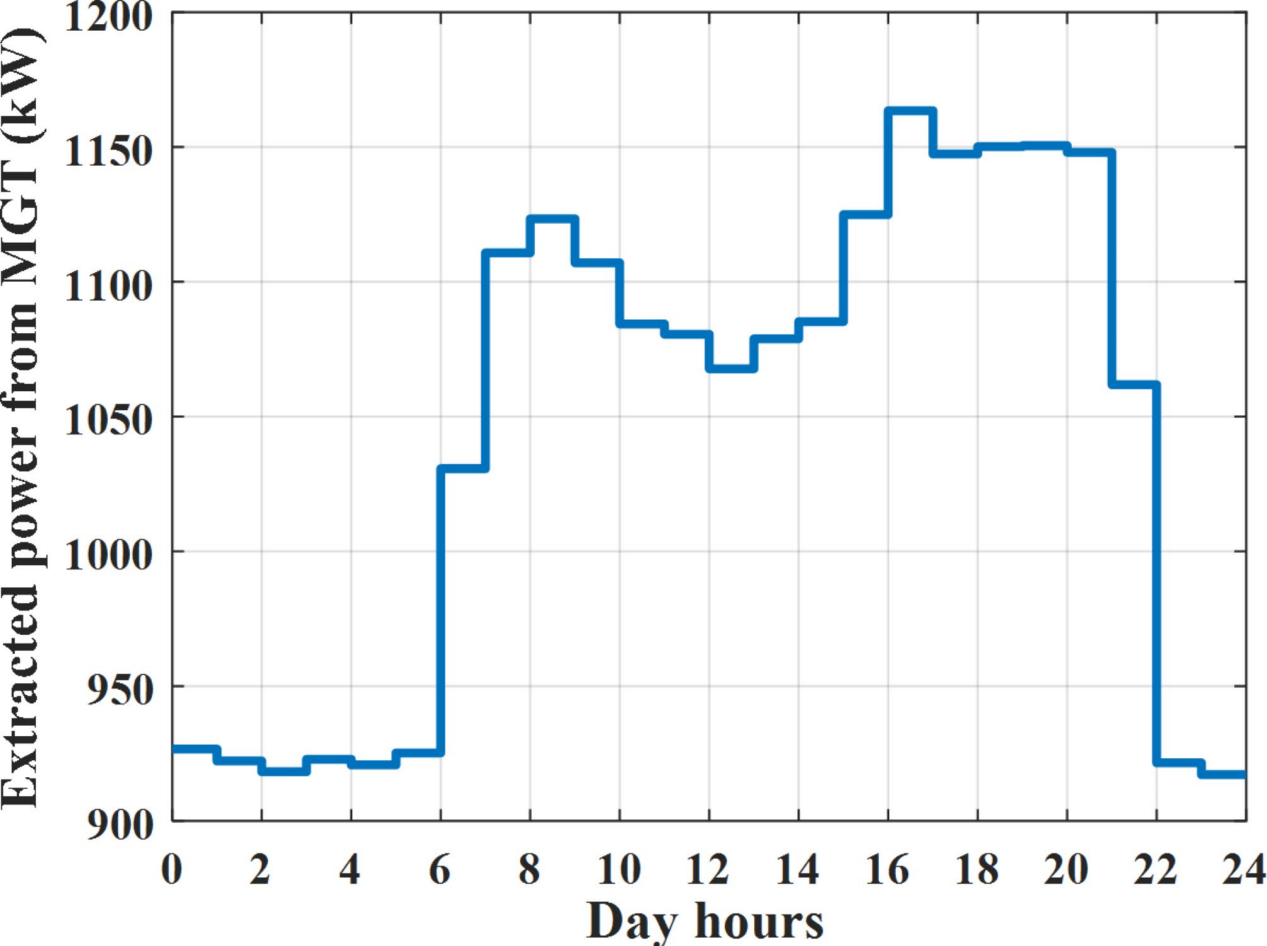
Extracted power from MGT all over the day during autonomous mode before load management.

**Fig. 25:**
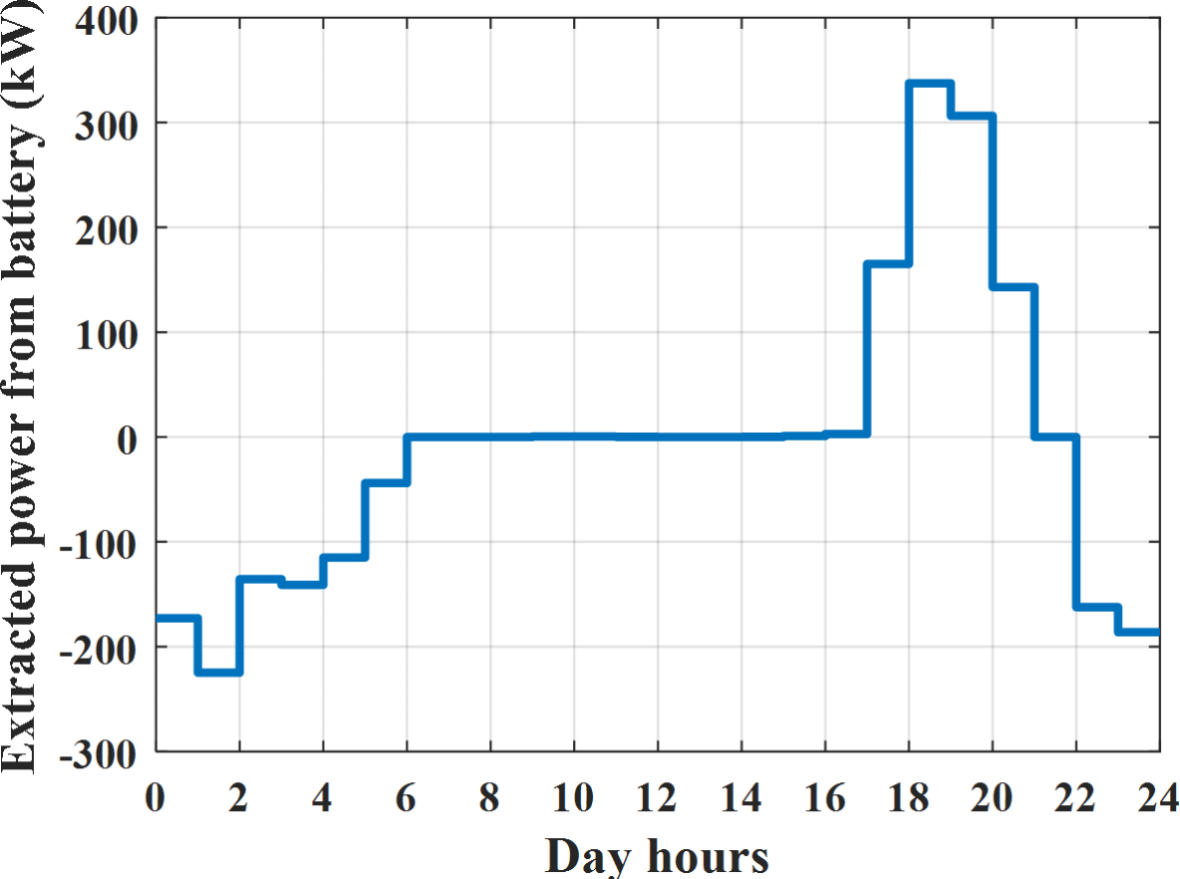
Extracted power from battery all over the day during autonomous mode before load management.

**Fig. 26:**
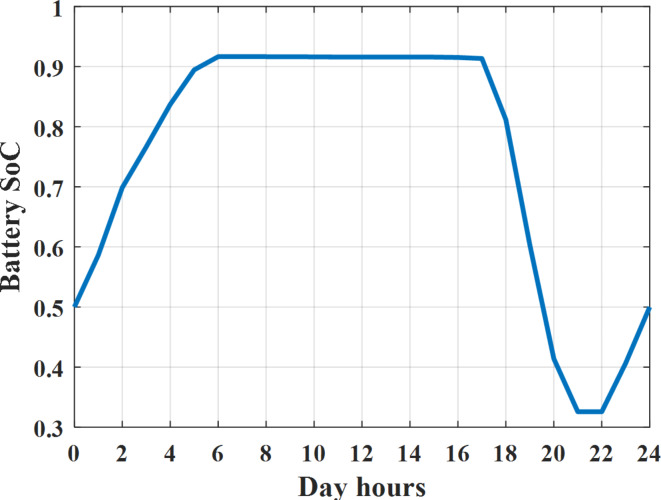
SoC of battery all over the day during autonomous mode before load management.

The power extracted from diesel generator is indicated in Fig. 23 that dedicate the maximum power obtained of about 923 kW per hour (4–5) pm and minimum power obtained of about 726 kW per hour (11–12) pm. The power extracted from MGT is indicated in Fig. 24 that dedicate the maximum power obtained of about 1163 kW per hour (4–5) pm and minimum power obtained of about 917 kW per hour (11–12) pm (Fig. 25). The battery SoC is indicated in Fig. 26 while the total power extracted from battery is indicated in Fig. 25. From Fig. 25 it is cleared that the maximum power extracted from battery is about 338 kW per hour (6–7) pm.

To check the power balance between total generation and total required during the day hours the following examples for random hours will be dedicated. For example, per hour 4 am the total required load is about 3236 kW as indicated by Figs. 4, 5 and 6, so the total power from different sources should satisfy the power required for load. In this case (4 am) it is found that the power extracted from PV is about 27 kW as indicated in Fig. 13, the power extracted from wind is about 133 kW as indicated in Fig. 14, the power extracted from one diesel generator is about 730 as indicated in Fig. 23 as mentioned above in the proposed model there are two diesel generators so the total power from two diesel generators are 1460 kW, the power extracted from MGT is about 922 kW as indicated in Fig. 24, and the power extracted from fuel cell is about 1000 kW. The sum of the generated power is about 3542 kW while the total demand is about 3236 so there is an additional power of about 306 kW which will be taken by two batteries each one of about 150 kW as indicated in Fig. 25 (charging mode). The battery initial SoC is about 50% at 1 am (at the beginning of the day) and returns to its initial value at 12 pm (at the end of the day) as indicated in Fig. 26.

Another example to check the power balance between total generation and total required during the day hours, per hour at 12pm the total required load is about 3542 kW as indicated by Figs. 4, 5 and 6, so the total power from different sources should satisfy the power required for load. In this case (12 pm) it is found that the power extracted from PV is about 0 kW as indicated in Fig. 13 there is no power extracted from PV, the power extracted from wind is about 544 kW as indicated in Fig. 14, the power extracted from one diesel generator is about 726 as indicated in Fig. 23 as mentioned above in the proposed model there are two diesel generators so the total power from two diesel generators are 1452 kW, the power extracted from MGT is about 917 kW as indicated in Fig. 24, and the power extracted from fuel cell is about 1000 kW. The sum of the generated power is about 3914 kW while the total demand is about 3542 kW so there is an additional power of about 372 kW which will be taken by two batteries each one of about 186 kW as indicated in Fig. 25 (charging mode). The battery initial SoC is about 50% at 1 am (at the beginning of the day) and returns to its initial value at 12 pm (at the end of the day) as indicated in Fig. 26.

### Grid connected mode results after load management

**Fig. 27:**
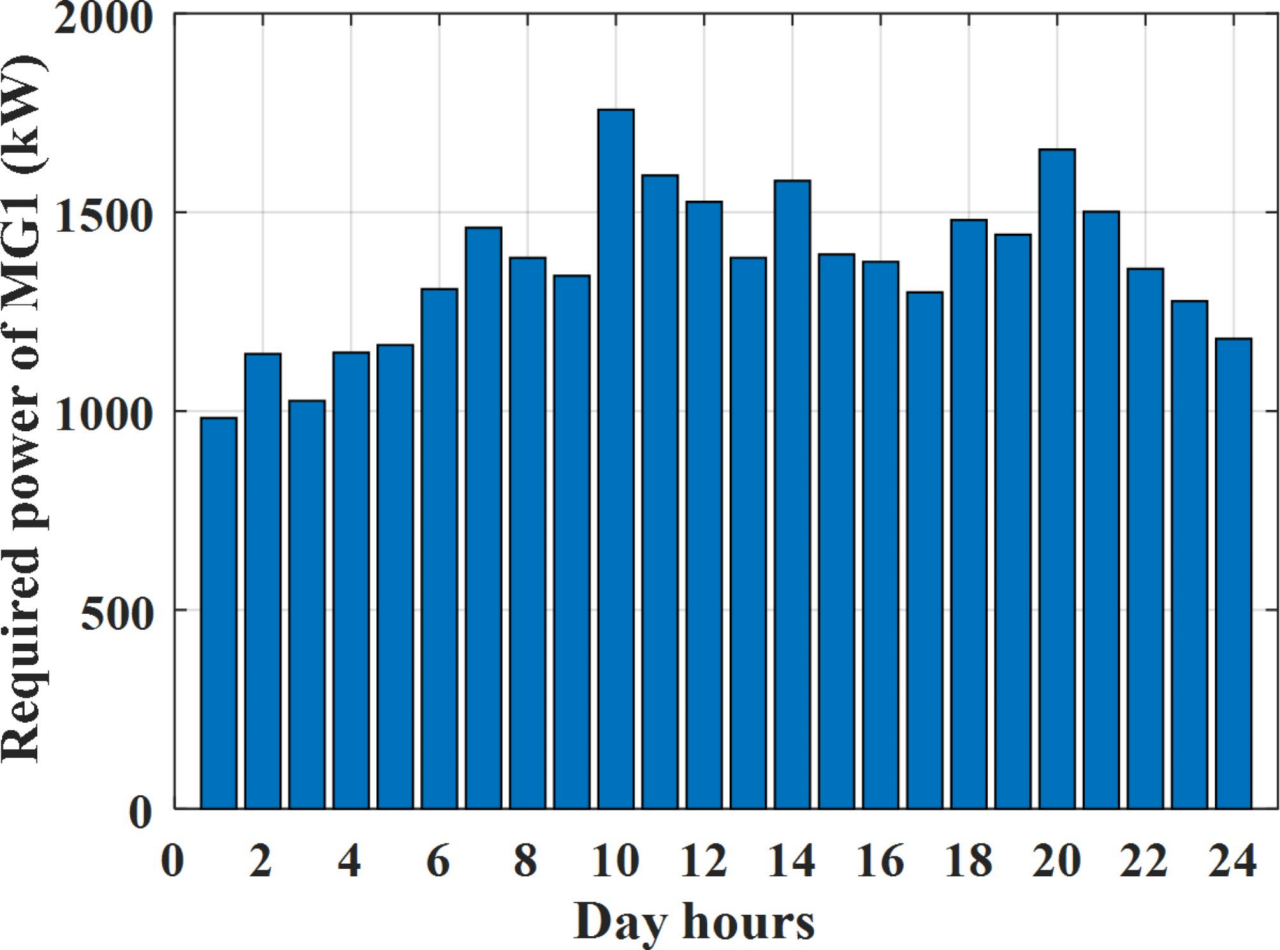
Required power for loads of MG1during grid connected mode after load management.

**Fig. 28:**
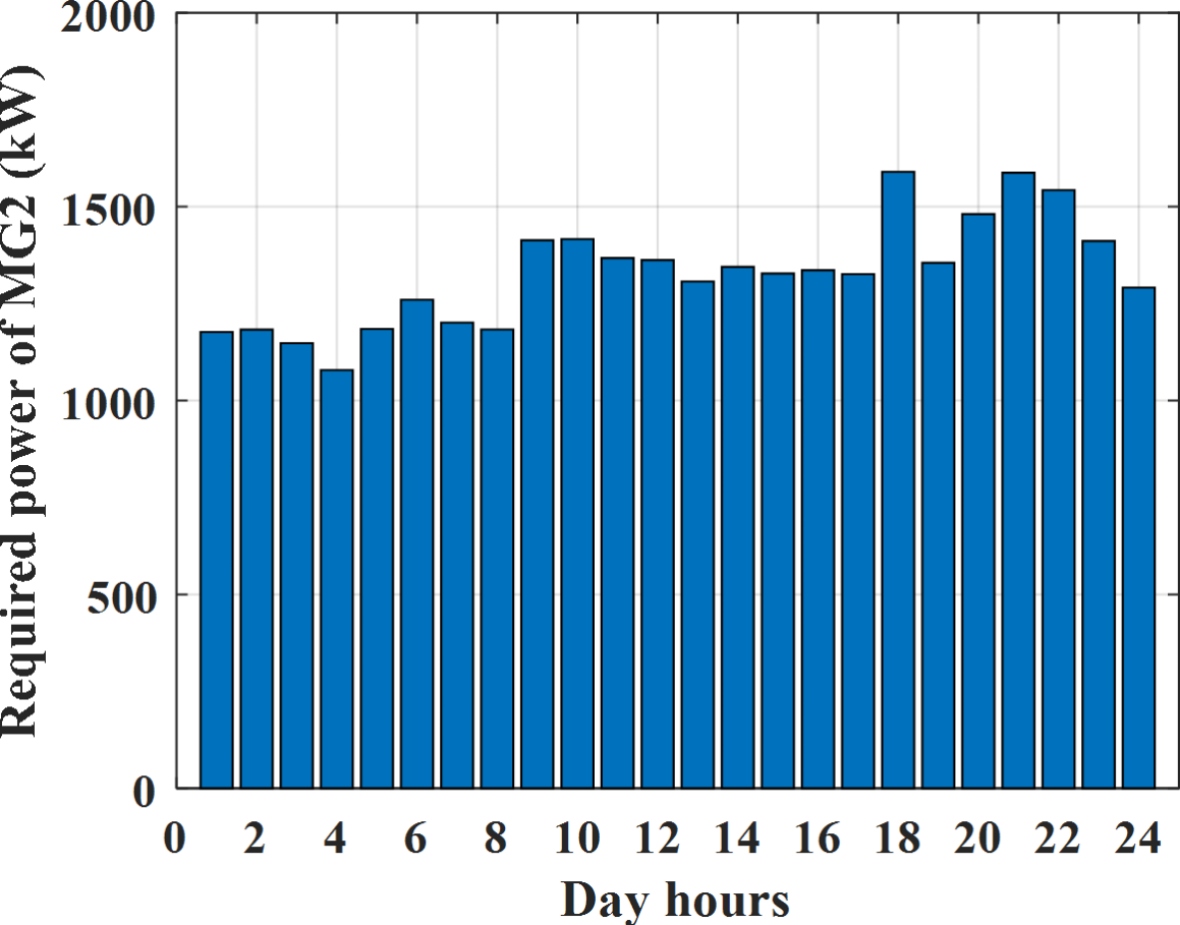
Required power for loads of MG2 during grid connected mode after load management.

**Fig. 29:**
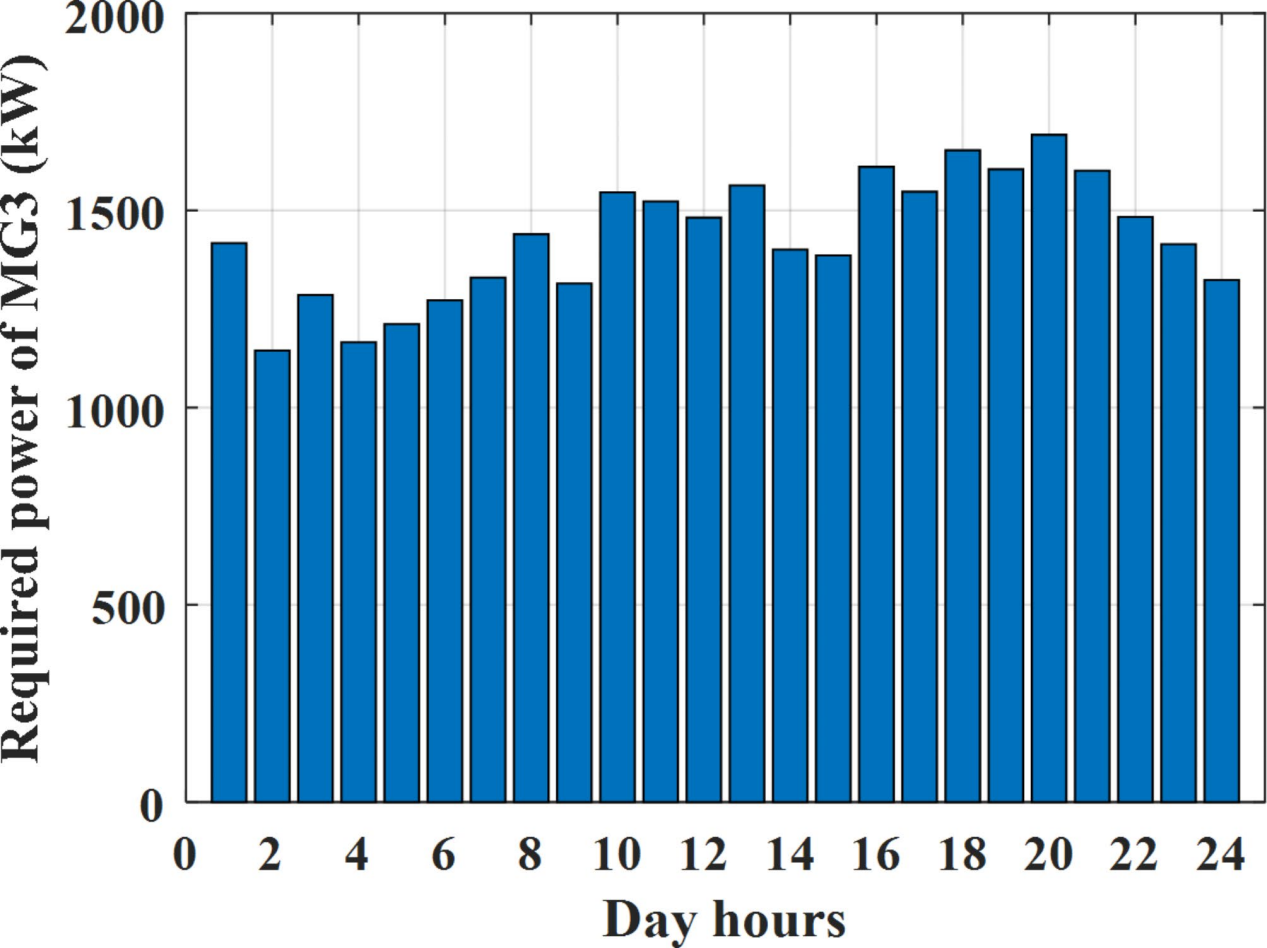
Required power for loads of MG3 during grid connected mode after load management.

**Fig. 30:**
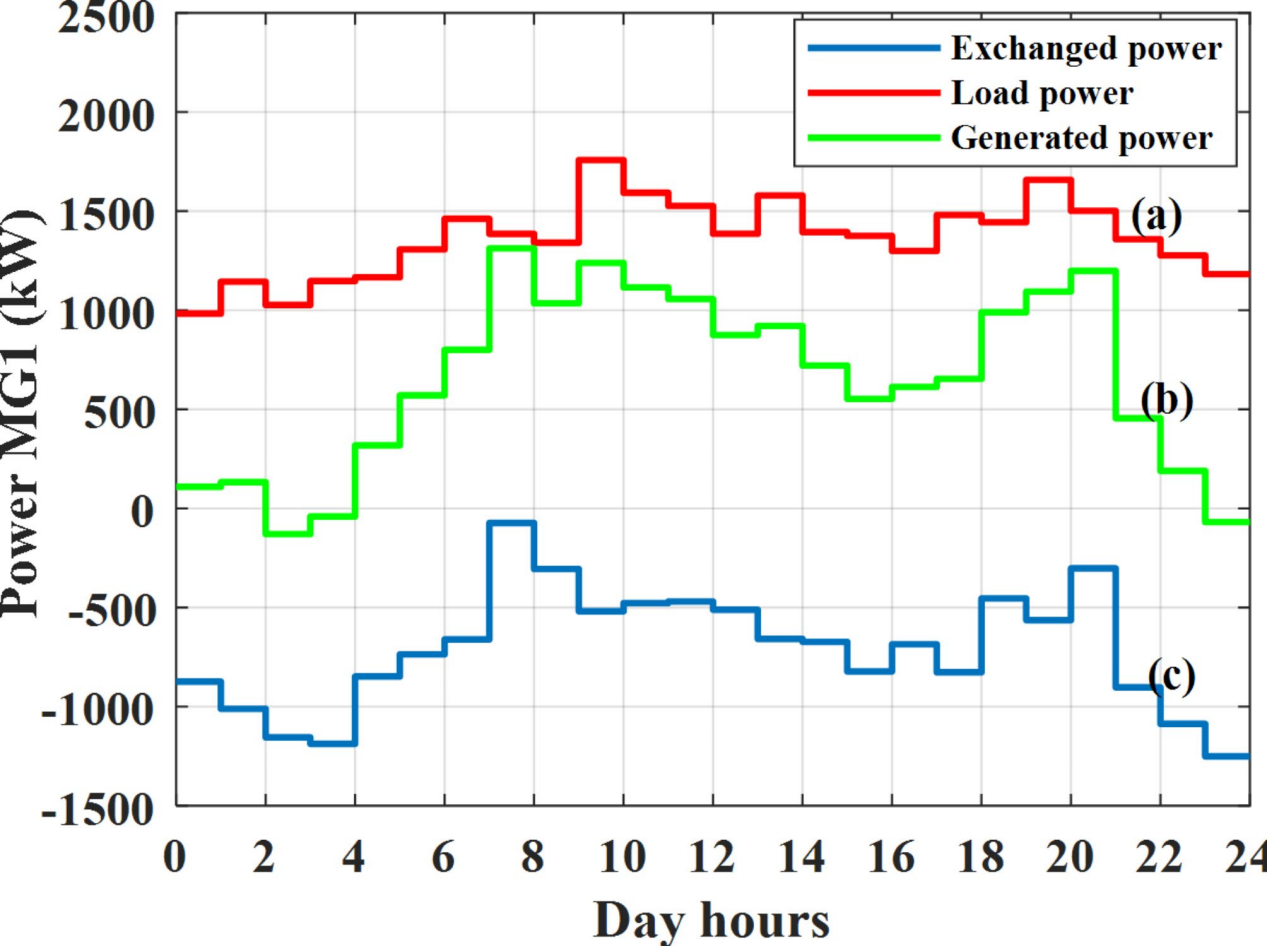
Power of MG1 all over the day during grid connected mode after load management.

**Fig. 31:**
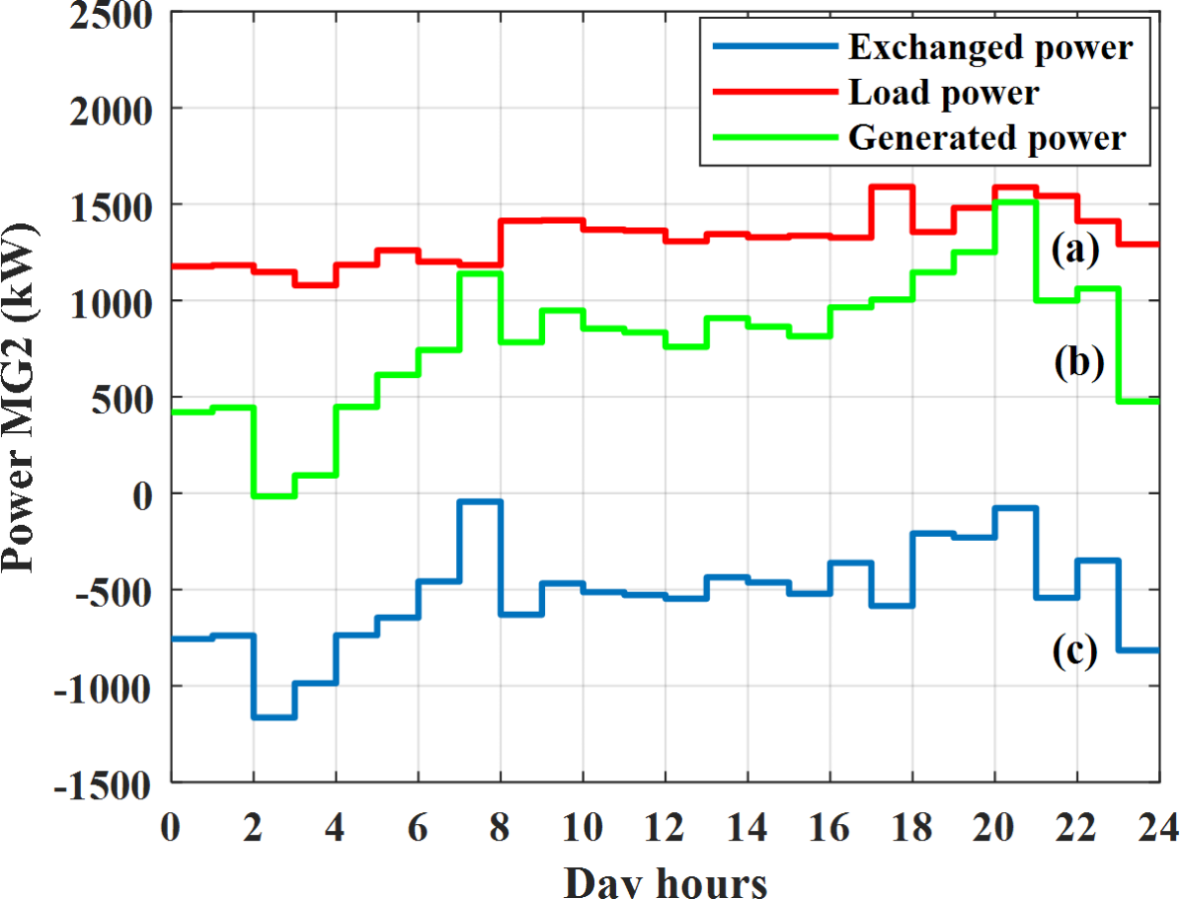
Power of MG2 all over the day during grid connected mode after load management.

**Fig. 32:**
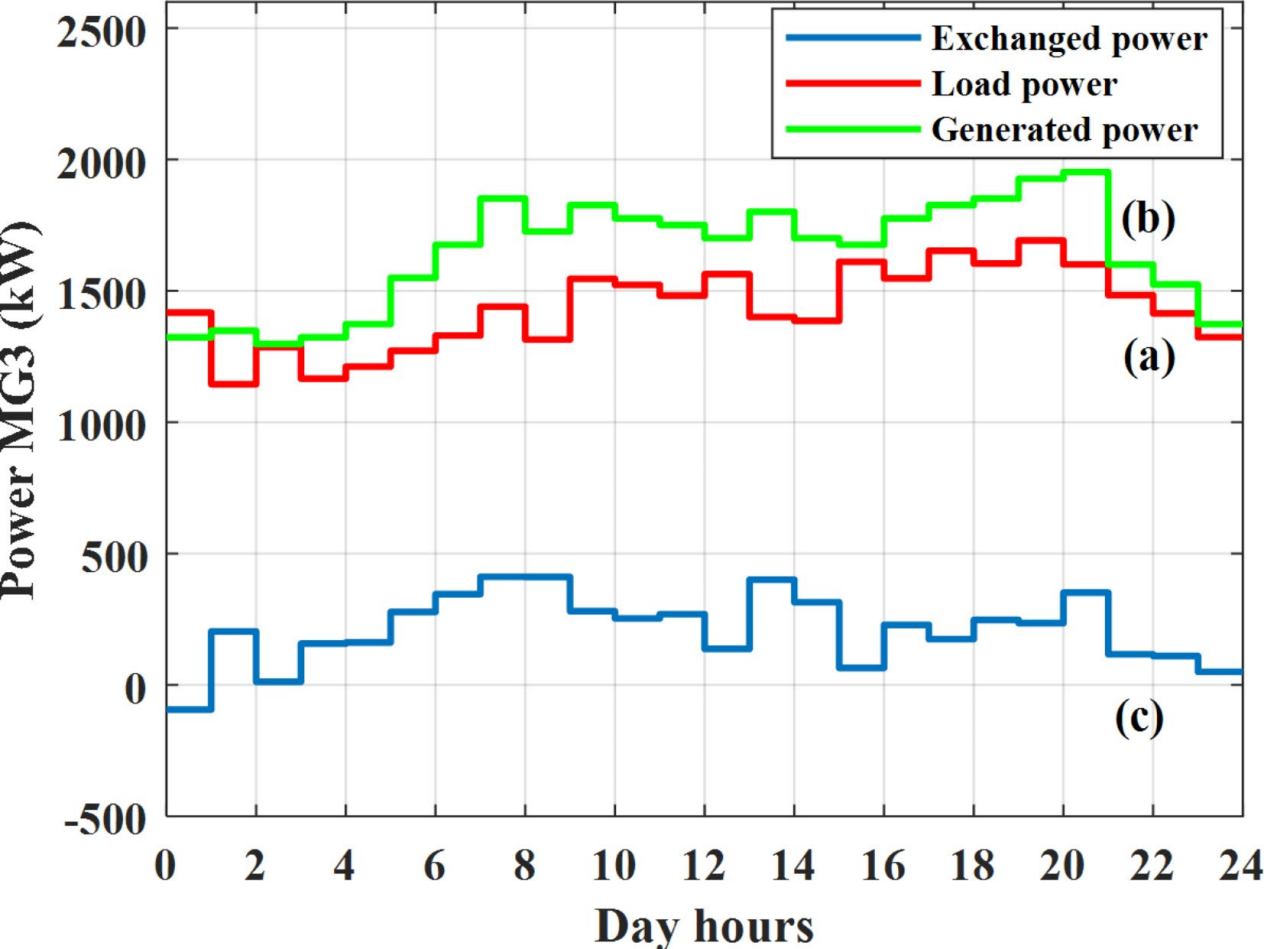
Power of MG3 all over the day during grid connected mode after load management.

The total required power for loads during the next day for MG1, MG2, and MG3 after load management is indicated in Figs. 27 and 28, and 29 consequently. The total power flow through the MG1 is indicated in Fig. 30 that illustrates the required power for loads that represented by Fig. 30a while the generated power from sources at MG1 is indicated by Fig. 30b. The difference between generated power and load power is the power exchanged from MG1 to other MGs and grid or power exchanged from MGs and grid to MG1 as indicated in Fig. 30c. The total power flow through the MG2 is indicated in Fig. 31 that illustrates the required power for loads that represented by Fig. 31a while the generated power from sources at MG2 is indicated by Fig. 31b. The difference between generated power and load power is the power exchanged from MG2 to other MGs and grid or power exchanged from MGs and grid to MG2 as indicated in Fig. 31c. The total power flow through the MG3 is indicated in Fig. 32 that illustrates the required power for loads that represented by Fig. 32a while the generated power from sources at MG3 is indicated by Fig. 32b. The difference between generated power and load power is the power exchanged from MG3 to other MGs and grid or power exchanged from MGs and grid to MG3 as indicated in Fig. 32c.

**Fig. 33:**
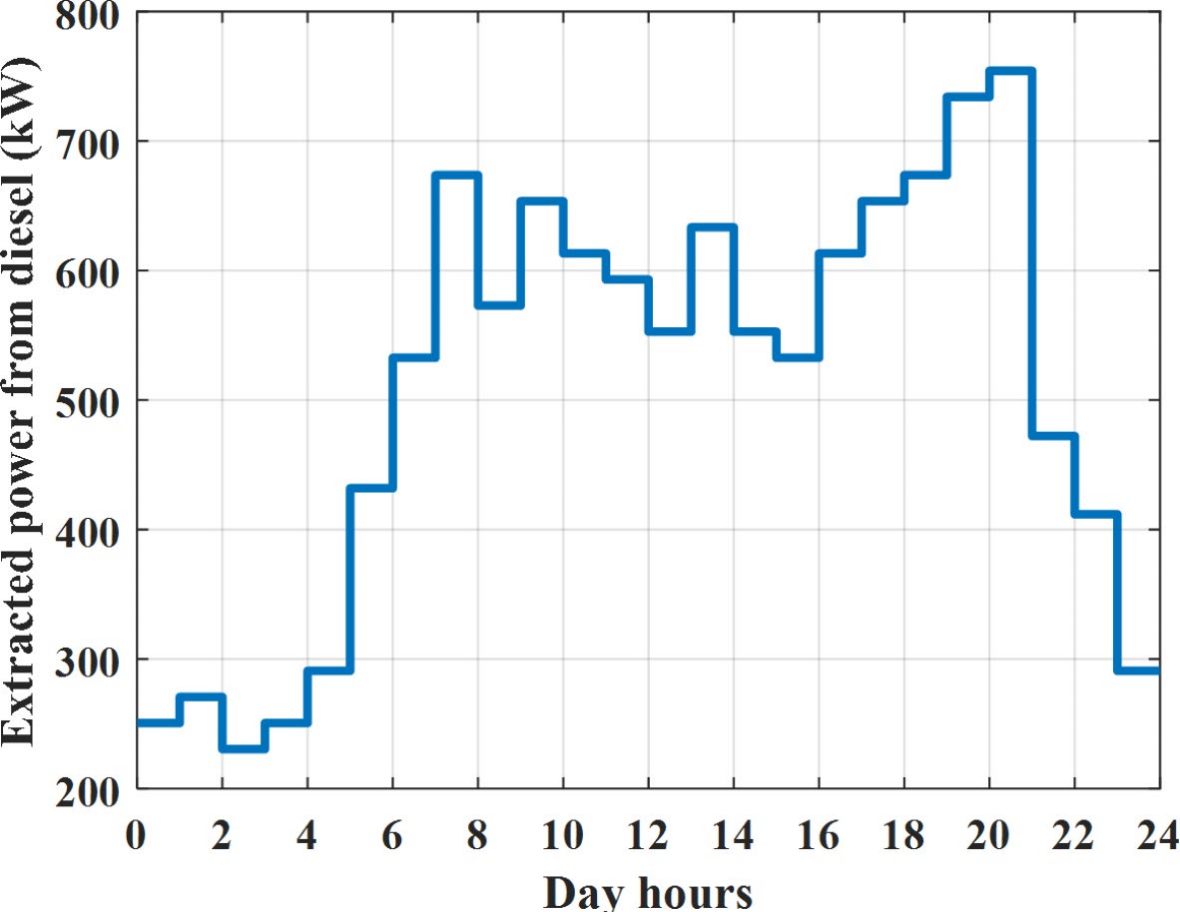
Extracted power from diesel all over the day during grid connected mode after load management.

**Fig. 34:**
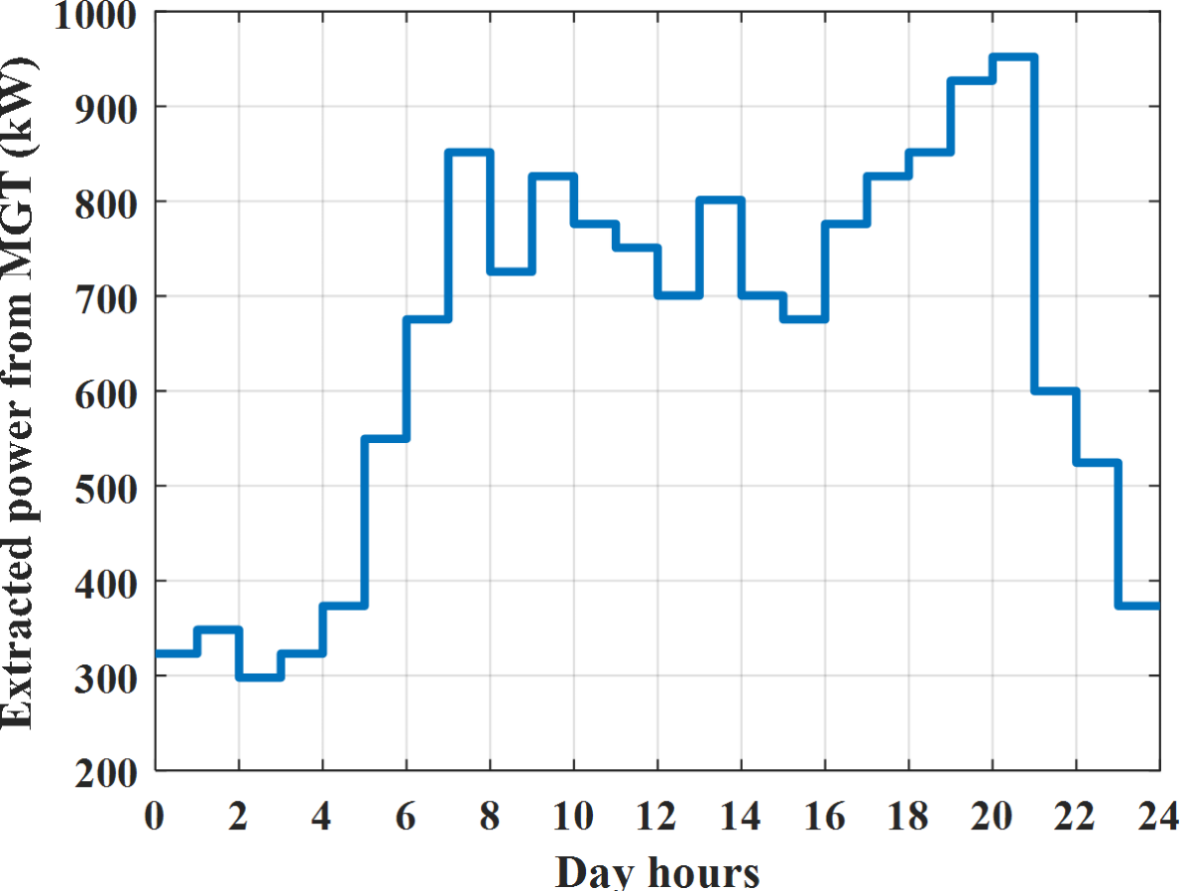
Extracted power from MGT all over the day during grid connected mode after load management.

**Fig. 35:**
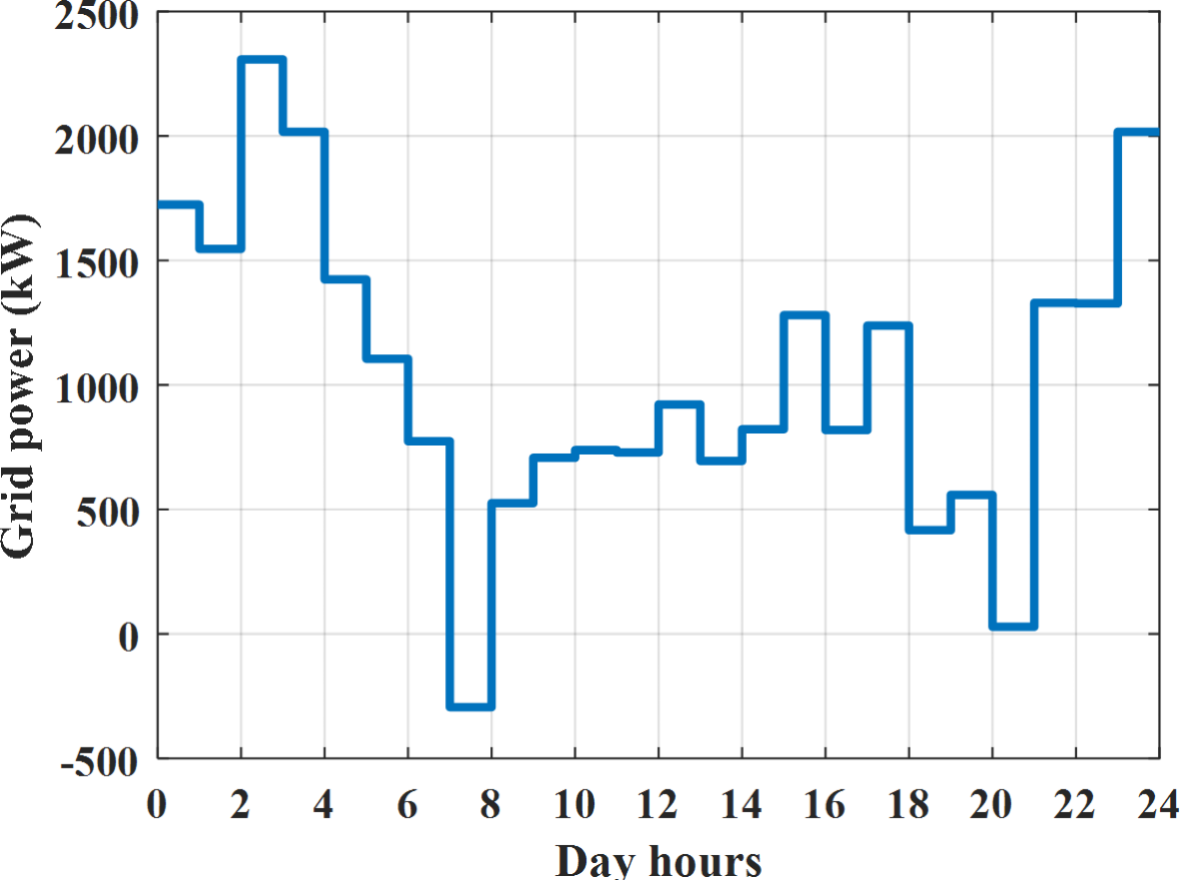
Utility power all over the day during grid connected mode after load management.

**Fig. 36:**
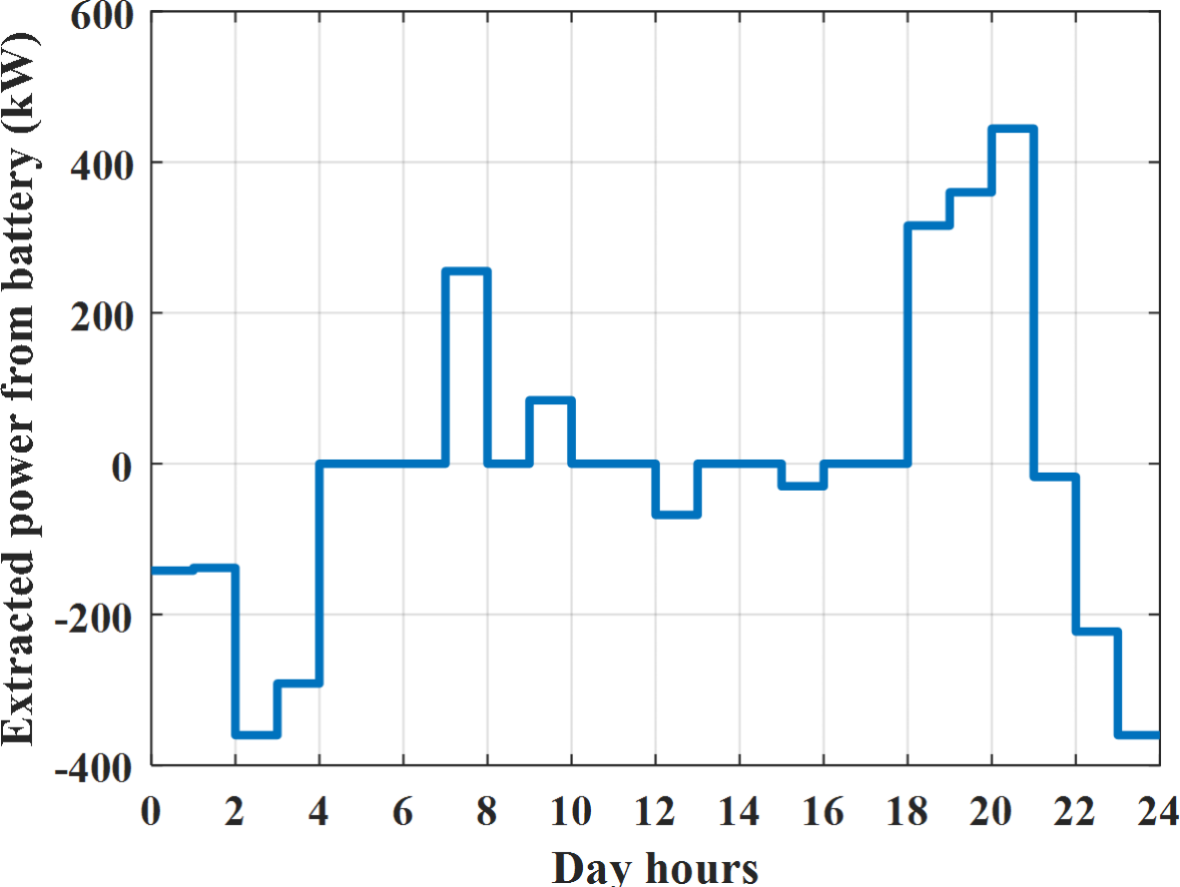
Extracted power from battery all over the day during grid connected mode after load management.

**Fig. 37:**
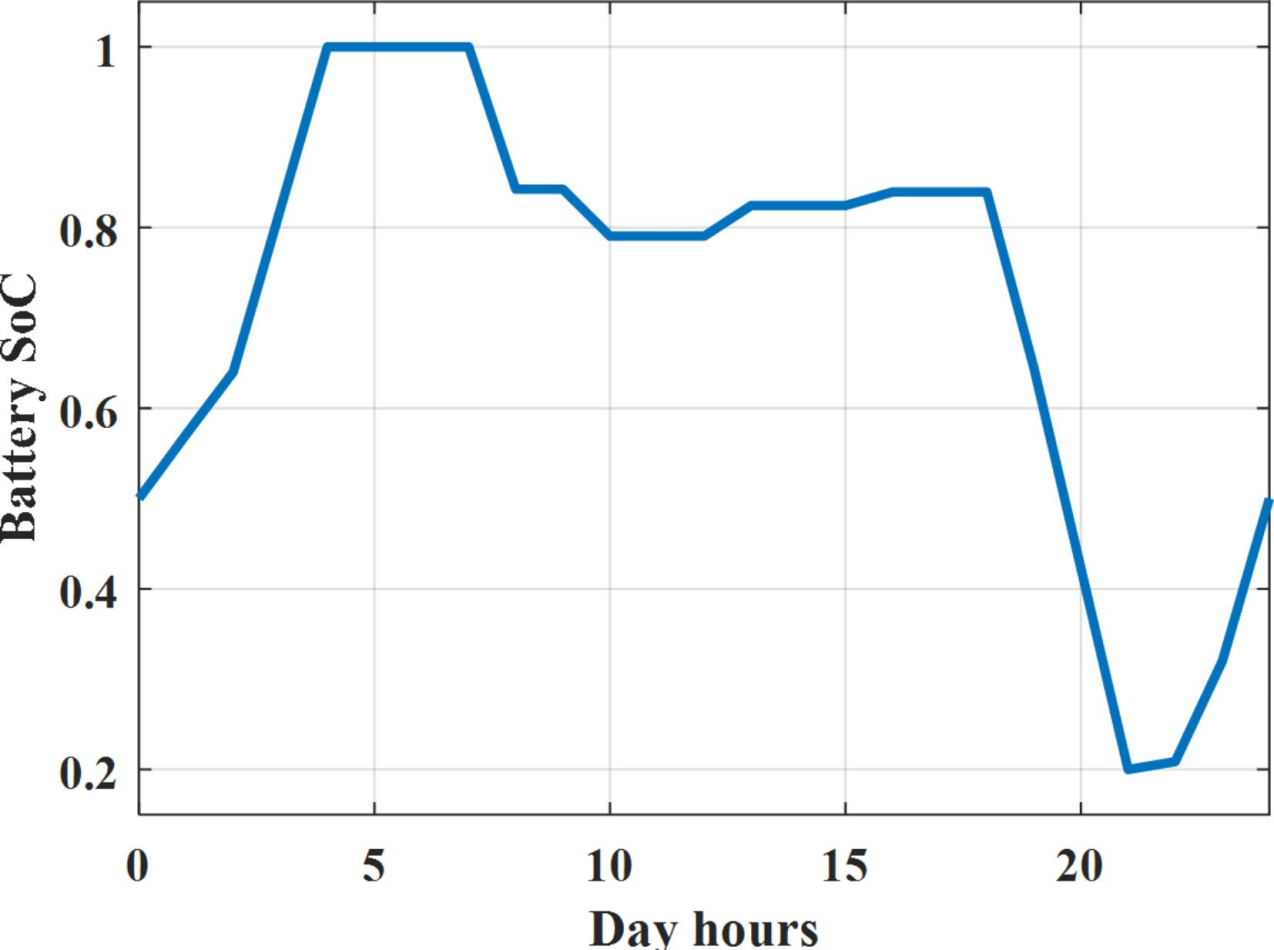
Percentage SoC of battery all over the day during grid connected mode after load management.

The extracted power from diesel generator during the day hours is indicated by Fig. 33. From Fig. 33 it is cleared that the minimum extracted power from diesel is about 230 kW per hour (2–3) am and maximum power extracted from diesel generator is about 754 kW per hour (8–9) pm. The extracted power from MGT generator during the day hours is indicated by Fig. 34. From Fig. 34 it is cleared that the minimum extracted power from MGT is about 300 kW per hour of (2–3) am and maximum power extracted from MGT is about 952 kW per hour (8–9) pm. The power utilized from utility during the day hours is indicated by Fig. 35. From Fig. 35 it is cleared that during all day hours there is a power taken from the grid except (7–8) am there is a power of 294 kW is transferred from all MGs to utility. The maximum power taken from grid is about 2307 kW per hour (2–3) am while minimum power taken from grid is about 28 kW per hour (8–9) pm (Fig. 36). The battery SoC is indicated in Fig. 37 while the total power extracted from battery is indicated in Fig. 36. From Fig. 36 it is cleared that the maximum power extracted from battery is about 444 kW per hour (8–9) pm.

**Fig. 38:**
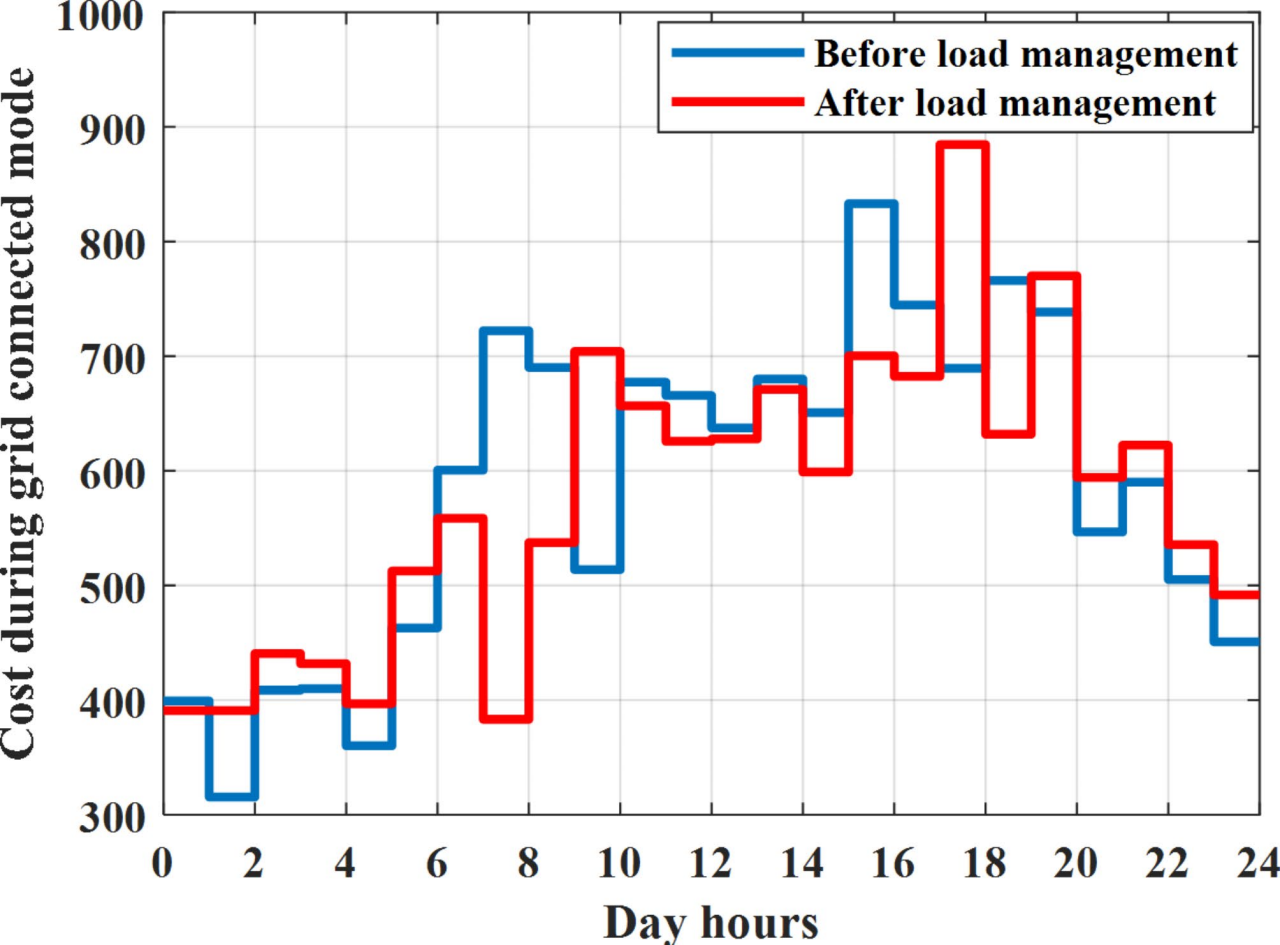
The operating cost of MG during grid connected mode.

To check power balance between generation and total demand during the day hours, per hour 4 pm the total required load is about 4322 kW as indicated by Figs. 27, 28, and 29, so the total power from different sources should satisfy the power required for load. In this case (4 pm) it is found that the power extracted from PV is about 50 kW as indicated in Fig. 13, the power extracted from wind is about 311 kW as indicated in Fig. 14, the power extracted from one diesel generator is about 533 as indicated in Fig. 33 as mentioned above in the proposed model there are two diesel generators so the total power from two diesel generators are 1066 kW, the power extracted from MGT is about 675 kW as indicated in Fig. 34, the power extracted from fuel cell is about 1000 kW, the total power taken from grid in this case is about 1280 as indicated in Fig. 35, there is a power of about 60 kW more than demand so this power will absorbed by two batteries each one with about 30 kW (charging mode) as indicated in Fig. 36. The battery initial SoC is about 50% at 1 am (at the beginning of the day) and return to its initial value at 12 pm (at the end of the day) as indicated in Fig. 37. The hourly operating cost of MG during grid connected mode is shown in Fig. 38. In grid-connected mode, implementation of the load management approach decreases the day-ahead operating cost of the MG from $14,084 to $13,858, resulting in a cost saving of about 1.6%.

### Autonomous mode results after load management

**Fig. 39:**
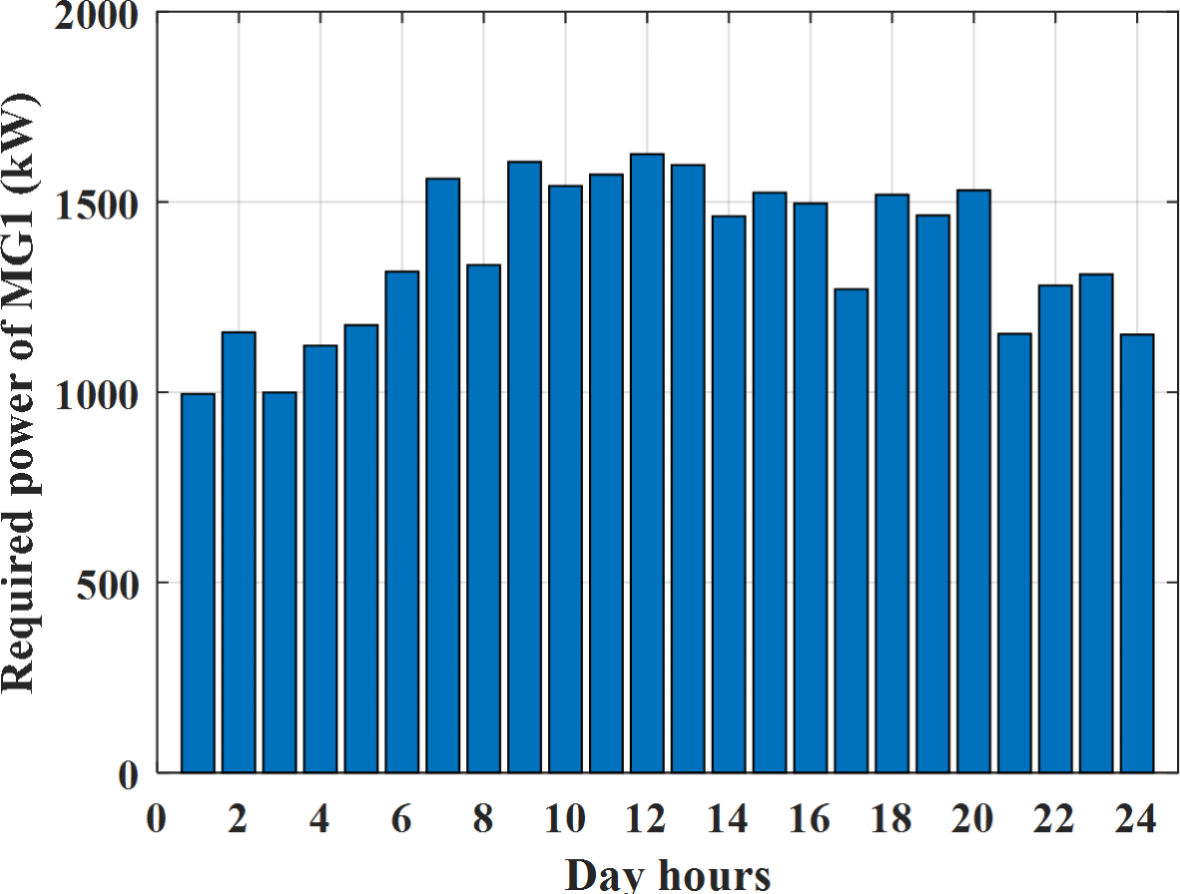
Required power for loads of MG1during autonomous mode after load management.

**Fig. 40:**
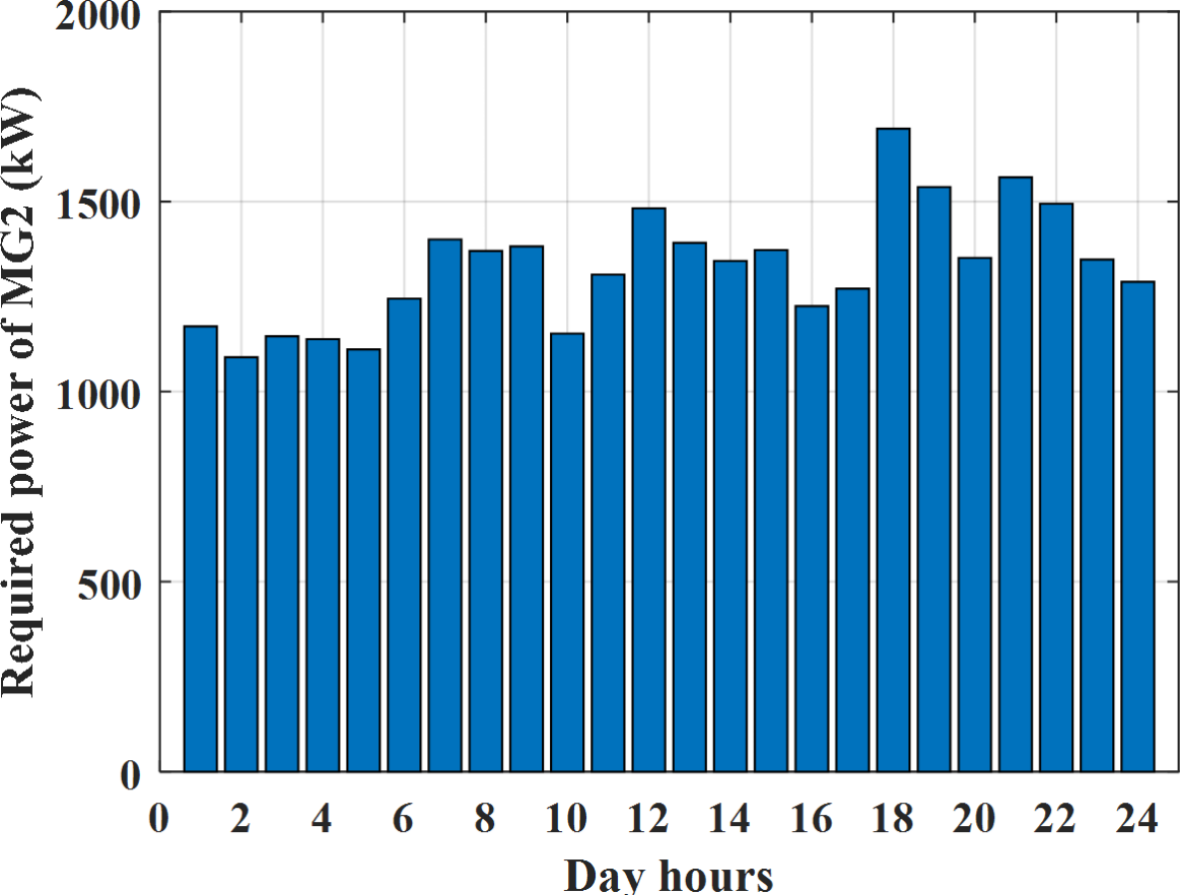
Required power for loads of MG2 during autonomous mode after load management.

**Fig. 41:**
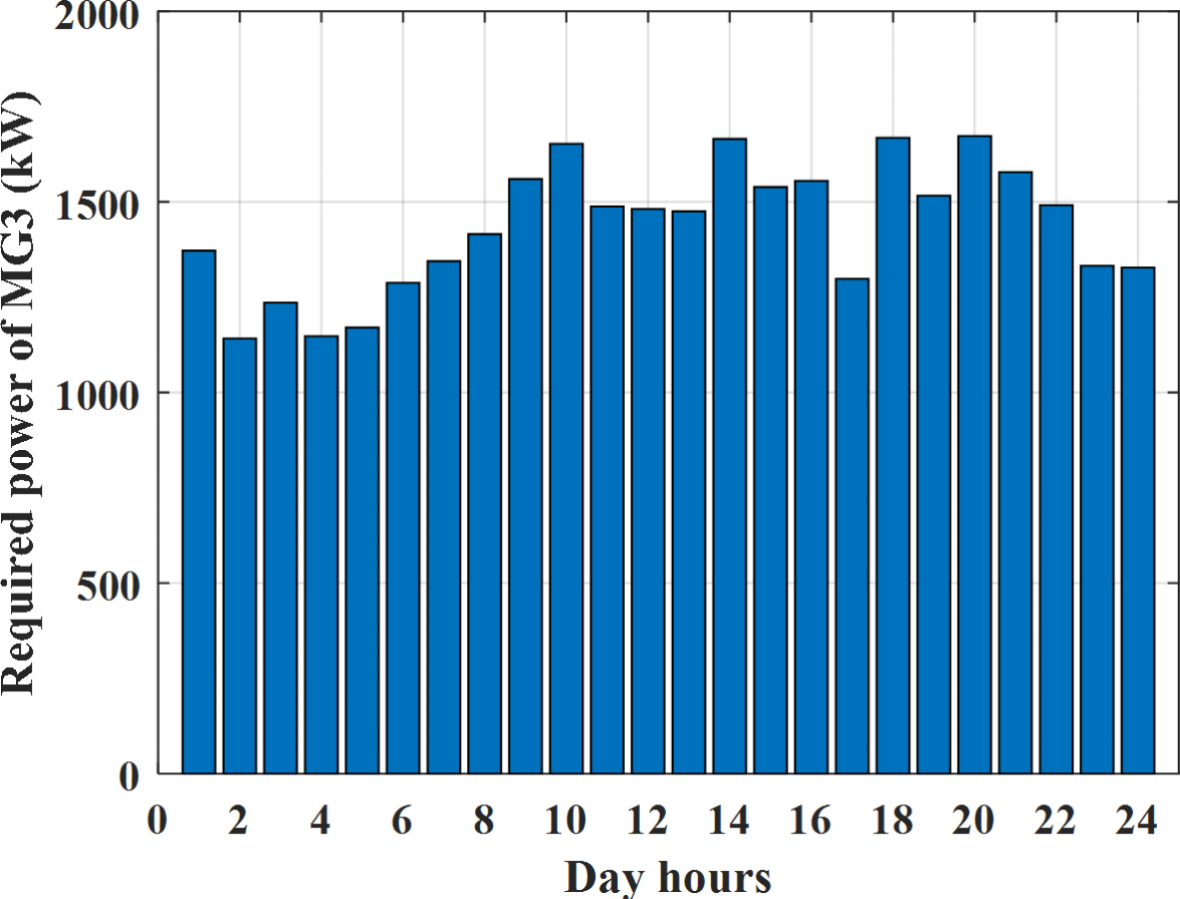
Required power for loads of MG3 during autonomous mode after load management.

**Fig. 42:**
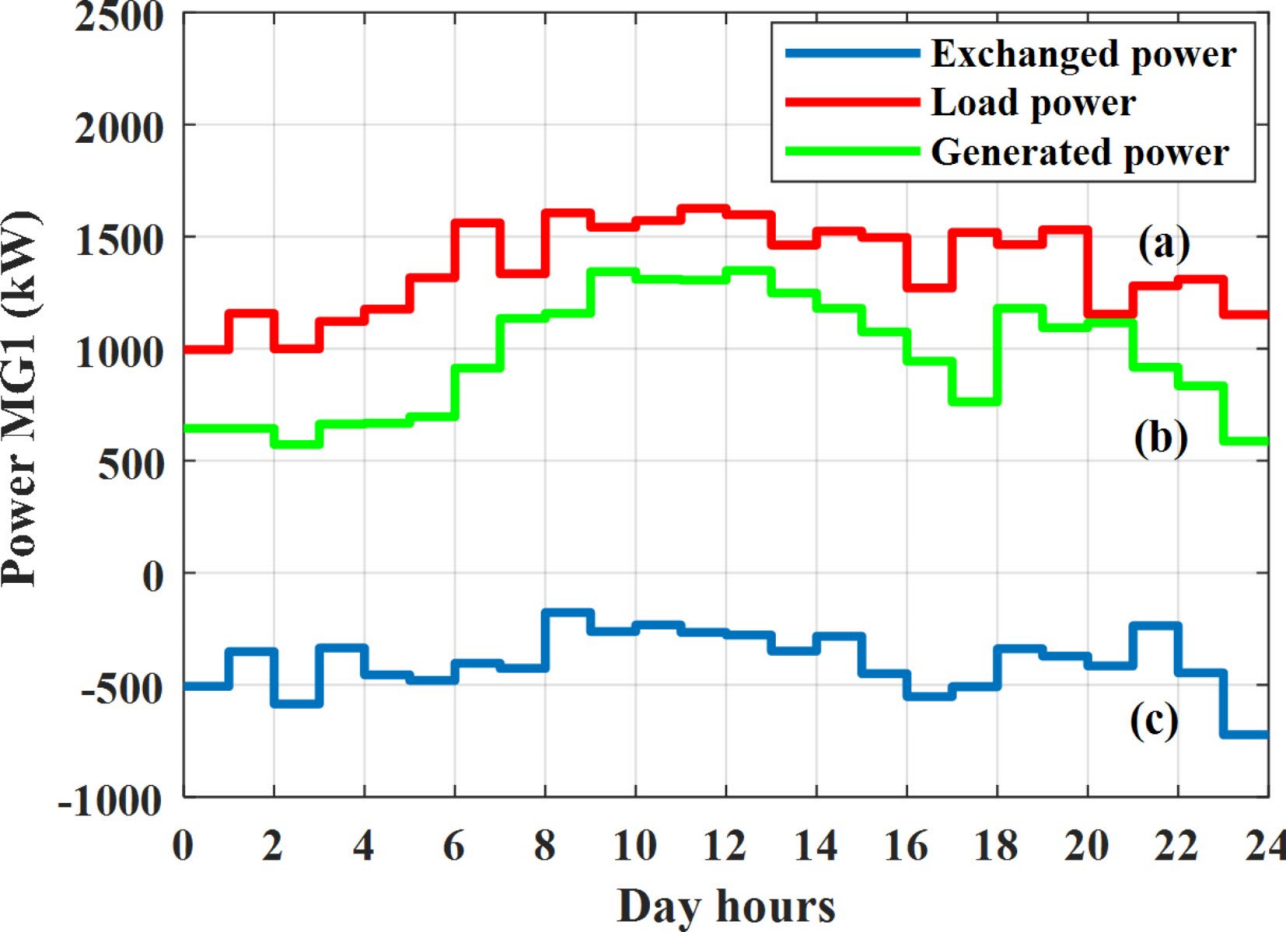
Power of MG1 all over the day during autonomous mode after load management.

**Fig. 43:**
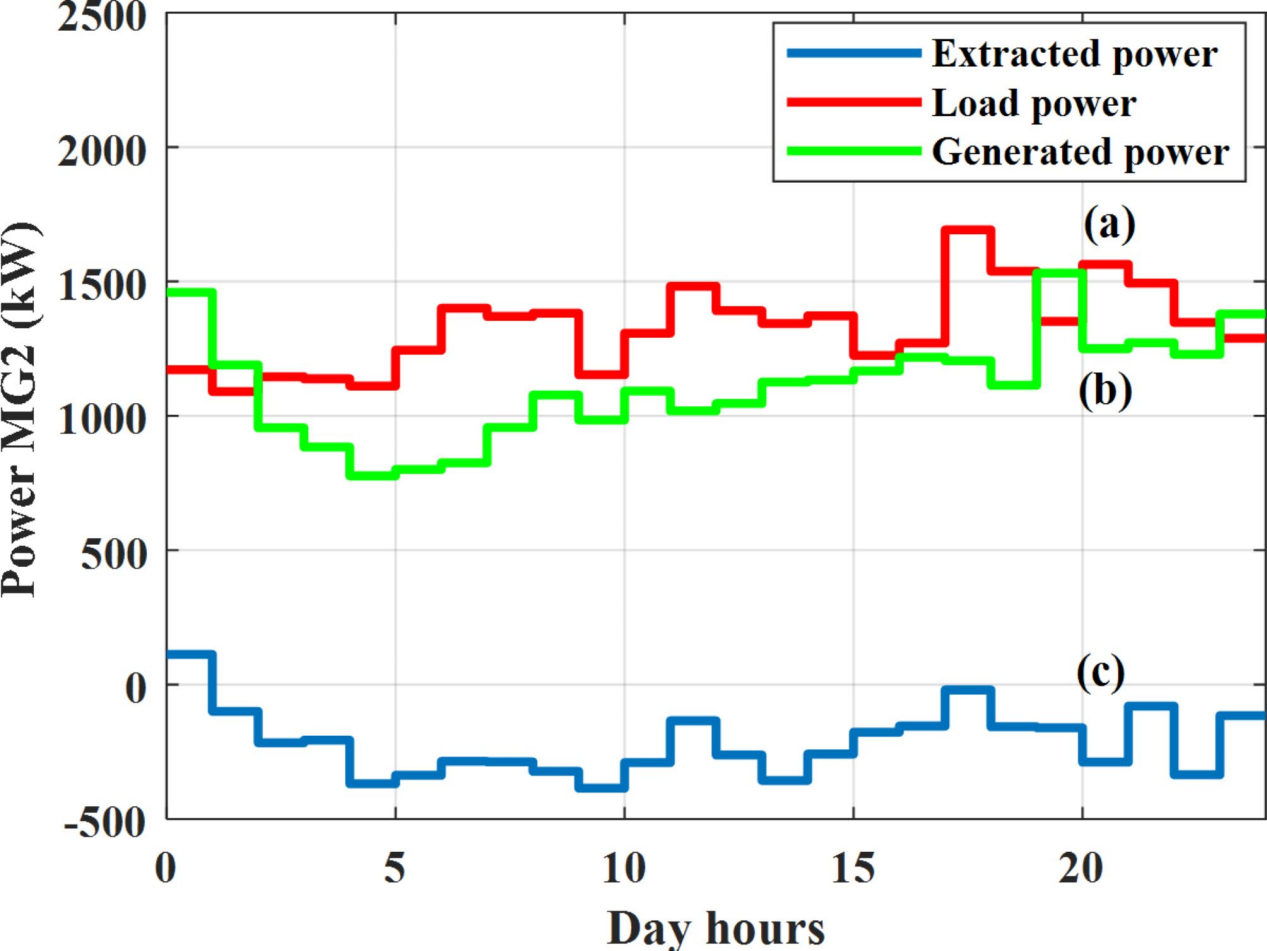
Power of MG2 all over the day during autonomous mode after load management.

**Fig. 44:**
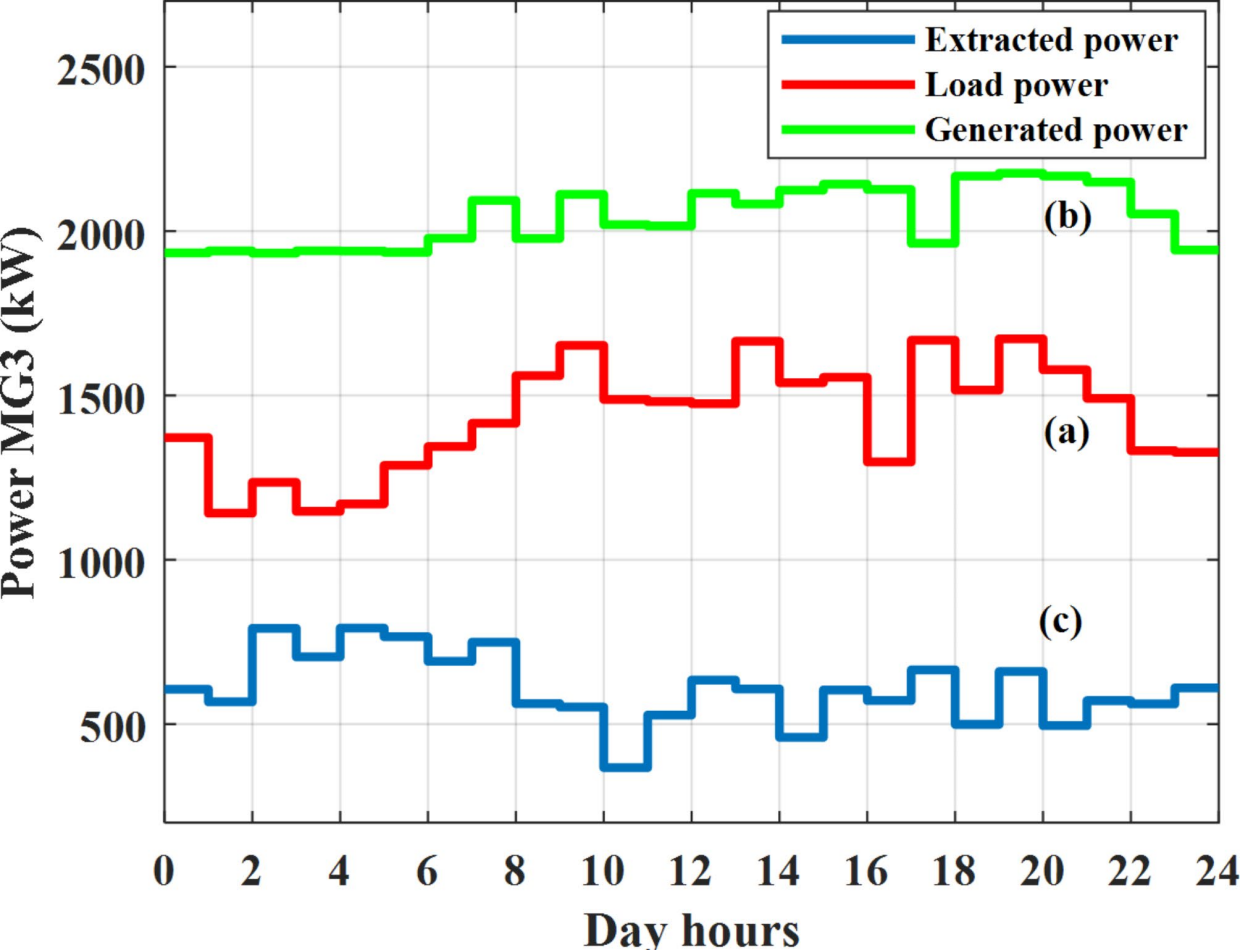
Power of MG3 all over the day during autonomous mode after load management.

The total required power for loads during the next day for MG1, MG2, and MG3 after load management is indicated in Figs. 39, 40, and 41 consequently. The total power flow through the MG1 is indicated in Fig. 42 that illustrates the required power for loads that represented by Fig. 42a while the generated power from sources at MG1 is indicated by Fig. 42b. The difference between generated power and load power is the power exchanged from MG1 to other MGs or power exchanged from MGs to MG1 as indicated in Fig. 42c. The total power flow through the MG2 is indicated in Fig. 43 that illustrates the required power for loads that represented by Fig. 43a while the generated power from sources at MG2 is indicated by Fig. 43b. The difference between generated power and load power is the power exchanged from MG2 to other MGs or power exchanged from MGs to MG2 as indicated in Fig. 43c. The total power flow through the MG3 is indicated in Fig. 44 that illustrates the required power for loads that represented by Fig. 44a while the generated power from sources at MG3 is indicated by Fig. 44b. The difference between generated power and load power is the power exchanged from MG3 to other MGs or power exchanged from MGs to MG3 as indicated in Fig. 44c.

**Fig. 45:**
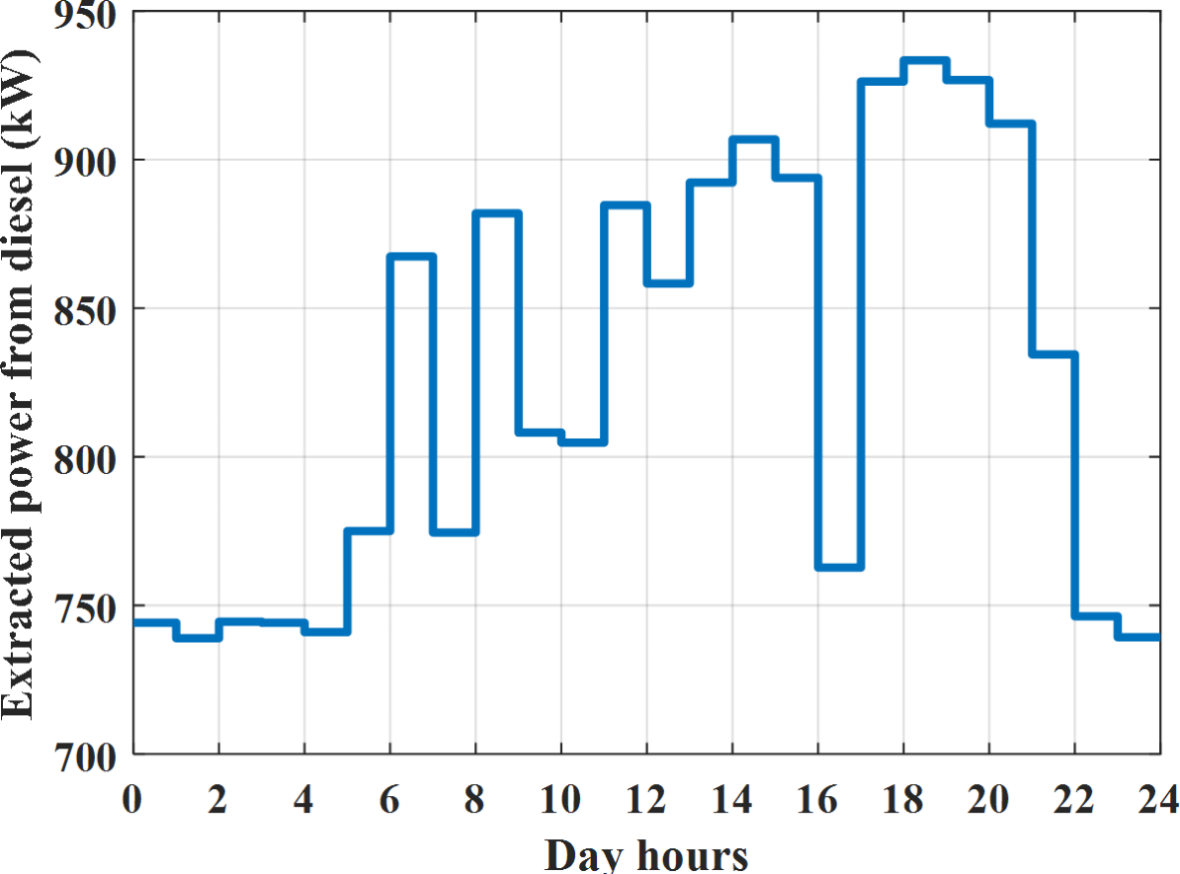
Extracted power from diesel all over the day during autonomous mode after load management.

**Fig. 46:**
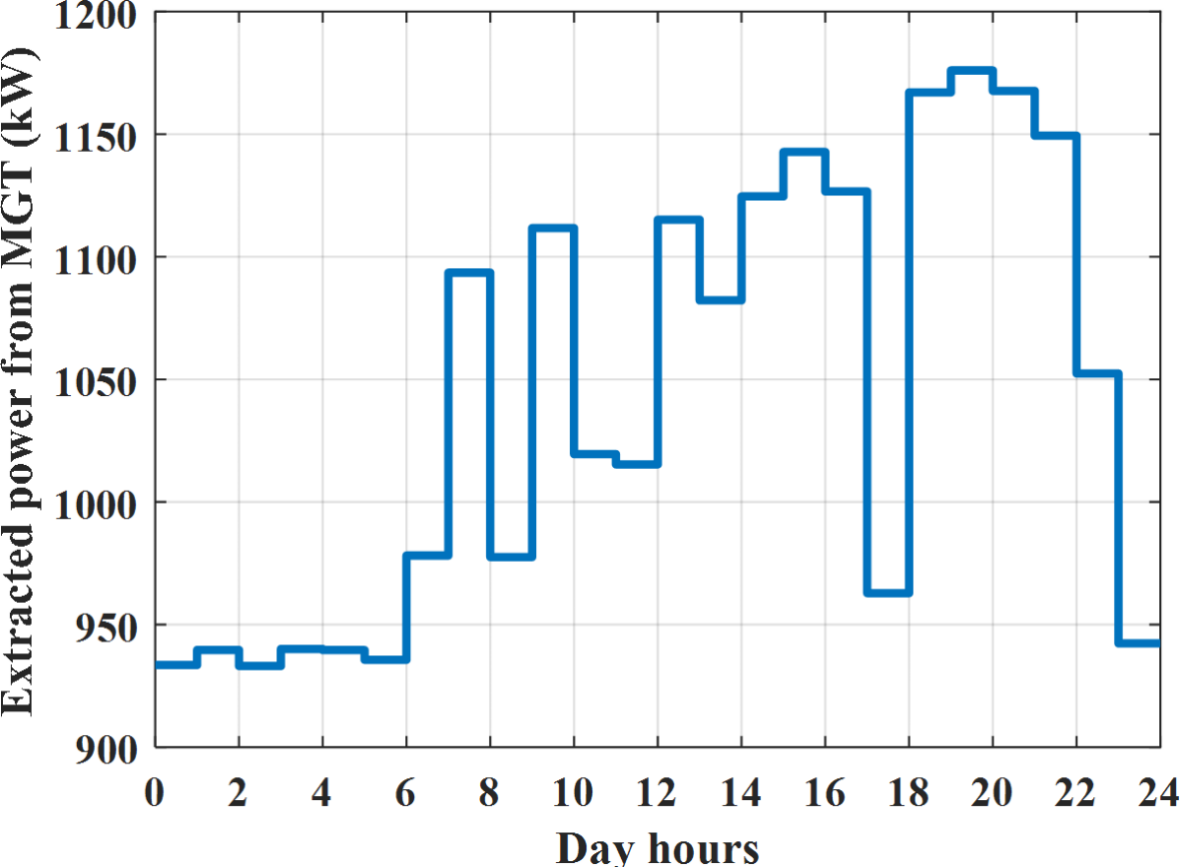
Extracted power from MGT all over the day during autonomous mode after load management.

**Fig. 47:**
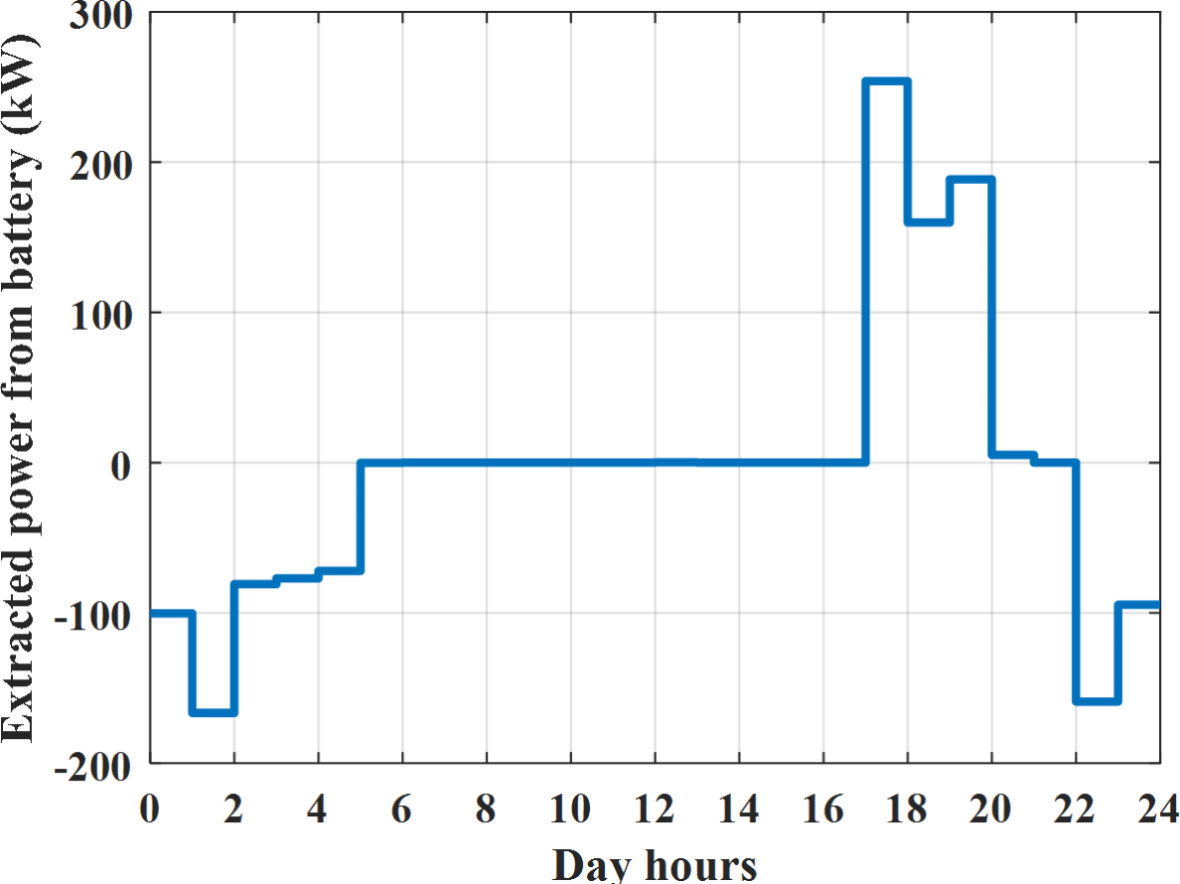
Extracted power from battery all over the day during autonomous mode after load management.

**Fig. 48:**
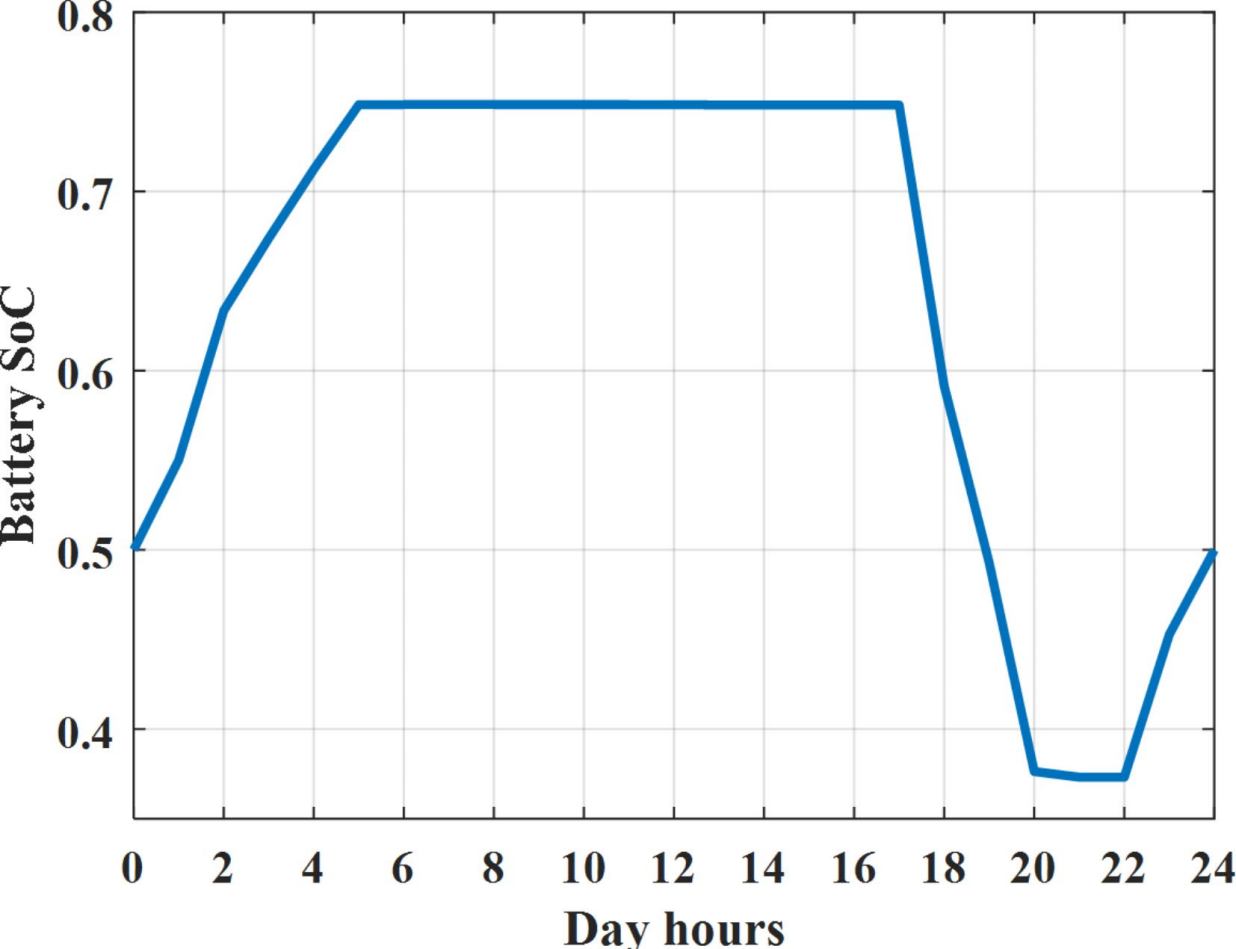
SoC of battery all over the day during autonomous mode after load management.

The power extracted from diesel generator is indicated in Fig. 45 that dedicate the maximum power obtained of about 933 kW per hour (6–7) pm and minimum power obtained of about 739 kW per hour (1–2) am. The power extracted from MGT is indicated in Fig. 46 that dedicate the maximum power obtained of about 1176 kW per hour (6–7) pm and minimum power obtained of about 933 kW per hour (2–3) am. The battery SoC is indicated in Fig. 47 while the total power extracted from battery is indicated in Fig. 47. From Fig. 48 it is cleared that the maximum power extracted from battery is about 254 kW per hour (5–6) pm.

**Fig. 49:**
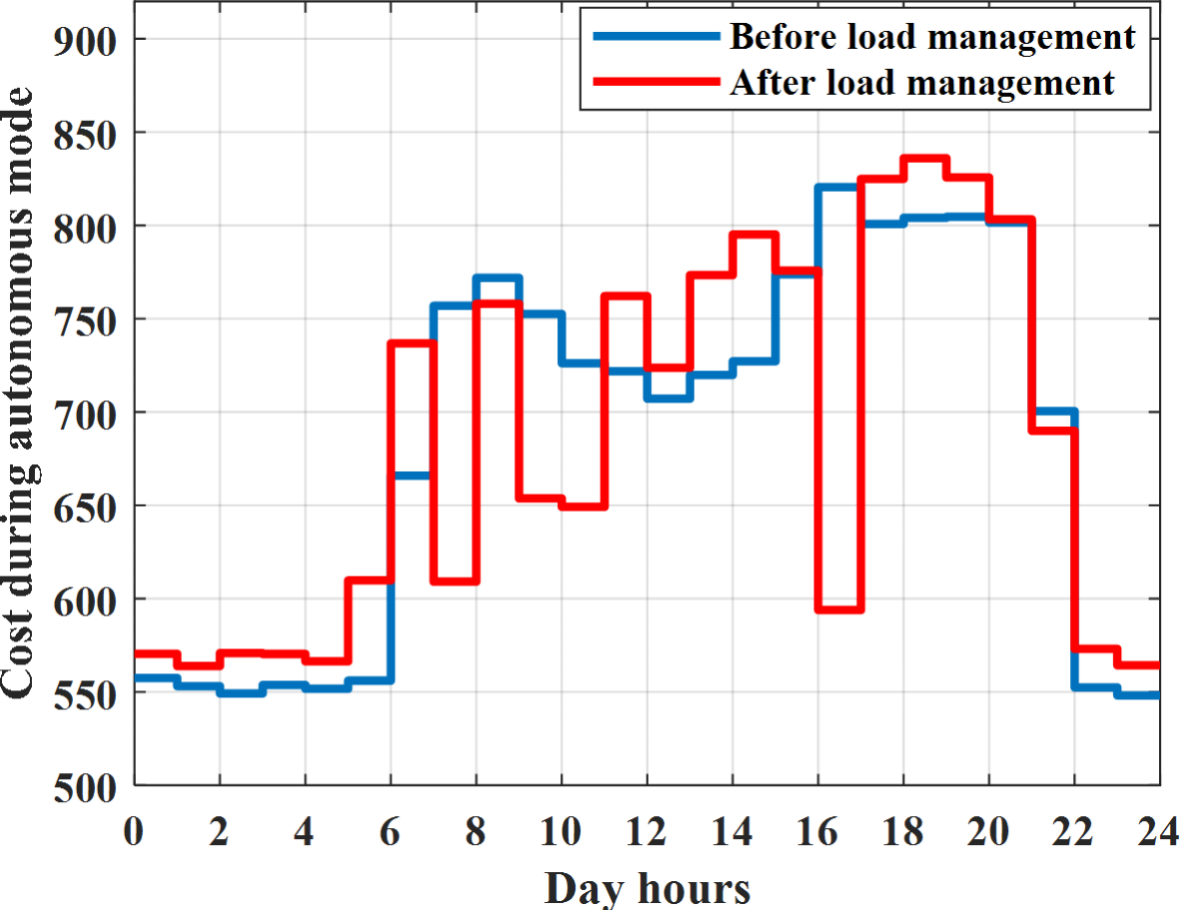
The operating cost of MG during islanded mode.

To check the power balance between total generation and total required during the day hours, per hour 12 pm the total required load is about 3767 kW as indicated by Figs. 39, 40, and 41, so the total power from different sources should satisfy the power required for load. In this case (12 pm) it is found that the power extracted from PV is about 0 kW as indicated in Fig. 13 there is no power extracted from PV, the power extracted from wind is about 544 kW as indicated in Fig. 14, the power extracted from one diesel generator is about 739 as indicated in Fig. 44 as mentioned above in the proposed model there are two diesel generators so the total power from two diesel generators are 1478 kW, the power extracted from MGT is about 933 kW as indicated in Fig. 46, and the power extracted from fuel cell is about 1000 kW. The sum of the generated power is about 3955 kW while the total demand is about 3767 kW so there is an additional power of about 188 kW which will be taken by two batteries each one of about 94 kW as indicated in Fig. 47 (charging mode). The battery initial SoC is about 50% at 1 am (at the beginning of the day) and return to its initial value at 12 pm (at the end of the day) as indicated in Fig. 48. The hourly operating cost of MG during islanded operation mode is shown in Fig. 49. In islanded operation mode, implementation of the load management approach decreases the day-ahead operating cost of the MG from $16,477 to $16,400, resulting in a cost saving of about 0.47%.

## Conclusions

This research proposes an effective energy management and demand side management strategy in a system made up of three interconnected microgrids (MGs). The multi-microgrid system can operate in two modes: grid-connected (with and without load management) and autonomous (with and without load management).

The major goal is to reduce daily operational costs while staying within technical restrictions and achieving a balance between power generation and load demand. To accomplish this, an innovative one-to-one based optimizer (OOBO) technique is used to calculate the optimal battery set points for both modes of operation. Furthermore, the Lagrange multiplier approach is used to determine the optimal set points for diesel generators and micro gas turbines (MGT) in both modes of operation.

A load management strategy is applied to shift the controllable loads from high-cost hours to low-cost hours. After applying the load management strategy and shifting the controllable loads from one interval to another, the day ahead operational cost is reduced by 1.6% for grid connected mode while it is reduced by 0.47% for islanded operation mode. The proposed energy management system intends to ensure effective energy consumption, save operational costs, and meet the power demand of interconnected microgrids by optimizing the set-points of various energy sources.

The proposed strategy is applied in a day ahead manner which may suffer from uncertainties in load and renewable generation during real time. Therefore, this work may be extended in the future to include applying an on-line EMS that can adapt with the real conditions of the MMGs system. Also, the operation of EMS under abnormal conditions, such as overloading, and the different kinds of failures at various locations in the MMGs, may be considered.

## Data Availability

All data generated or analyzed during this study are included in this published article.

## Abbreviations

DERs: Distributed energy resources
MGs: Microgrids
OOBO: One-to-one based optimizer
PV: Photovoltaic
WT: Wind turbine
FC: Fuel cell
MGT: Micro gas turbine
GHG: Greenhouse gases
AC: Alternative current
DC: Direct current
MMGs: Multi microgrids
FPI- MRAC: Fuzzy proportional integral based model reference adaptive controller
FFOPID: Fuzzy fractional order proportional integral derivative controller
FLC: Fuzzy logic control
PSO: Particle swarm optimization
MAS: Multi agent system
ANN: Artificial neural network
MPC: Model predictive control
GA: Genetic algorithm
MRF: Manta ray foraging
FPA: Flower pollination algorithm
AO: Aquila optimizer
EMS: Energy management system
MPPT: Maximum power point tracking
STC: Standard temperature condition
KE: Kinetic energy
SoC: State of charge
MILP: Mixed integer linear programming
EDN: Electricity distribution network
NESC: Neighborhood energy storage community

## References

[1] Sumarmad, K. A. A., Sulaiman, N., Wahab, N. I. A. & Hizam, H. Energy management and voltage control in microgrids using artificial neural networks, PID, and fuzzy logic controllers. *Energies* **15**(1), 1–22 (2022).

[2] Shahgholian, G. A brief review on microgrids: Operation, applications, modeling, and control. *Electrical Energy Systems* **33**(6), 1–28 (2021).

[3] Al-Ismail, F. S. DC microgrid planning, operation, and control: A comprehensive review. *IEEE Access* **9**, 36154–36172 (2021).

[4] Shahgholian, G. A brief review on microgrids: Operation, applications, modeling, and control. *Int. Trans. Electr. Energy Syst.* **31**(6), e12885 (2021).

[5] Shaker, H. K., Keshta, H. E., Mosa, M. A. & Ali, A. A. Improving the voltage response of grid connected three inter-connected microgrids using artificial intelligence based controllers. In *2023 IEEE International Conference on Advanced Systems and Emergent Technologies (IC_ASET), Hammamet, Tunisia* 1–6 (2023).

[6] Shaker, H. K., Keshta, H. E., Mosa, M. A. & Ali, A. A. Adaptive nonlinear controllers-based approach to improve the frequency control of multi islanded interconnected microgrids. *Energy Rep.* **9**, 5230–5245 (2023).

[7] Zhong, C., Zhou, Y., Chen, J. & Liu, Z. DC-side synchronous active power control of two-stage photovoltaic generation for frequency support in Islanded microgrids. *Energy Rep.* **8**, 8361–8371 (2022).

[8] Singh, K. & Arya, Y. Tidal turbine support in microgrid frequency regulation through novel cascade Fuzzy-FOPID droop in de-loaded region. *ISA Trans.* **133**, 218–232 (2023). 35879113 10.1016/j.isatra.2022.07.010

[9] Taghieh, A., Mohammadzadeh, A., Zhang, C., Kausar, N. & Castillo, O. A type-3 fuzzy control for current sharing and voltage balancing in microgrids. *Appl. Soft Comput.* **129**, 109636 (2022).

[10] Alghamdi, B. & Cañizares, C. Frequency and voltage coordinated control of a grid of AC/DC microgrids. *Appl. Energy* **310**, 118427 (2022).

[11] Khan, M. K. et al. Noman Mujeeb Khan, Green energy extraction for sustainable development: A novel MPPT technique for hybrid PV-TEG system. *Sustain. Energy Technol. Assessm.* **53**, 102388 (2022).

[12] Li, C., Jia, X., Zhou, Y. & Li, X. A microgrids energy management model based on multi-agent system using adaptive weight and chaotic search particle swarm optimization considering demand response. *J. Clean. Prod.* **262**, 0959–6526 (2020).

[13] Aguila-Leon, J., Vargas-Salgado, C., Chiñas-Palacios, C. & Díaz-Bello, D. Energy management model for a standalone hybrid microgrid through a particle swarm optimization and artificial neural networks approach. *Energy Convers. Manage.* **267**, 115920 (2022).

[14] Shan, Y., Hu, J. & Liu, H. A holistic power management strategy of microgrids based on model predictive control and particle swarm optimization. *IEEE Trans. Ind. Inf.* **18**(8), 5115–5126 (2022).

[15] Torkan, R., Ilinca, A. & Ghorbanzadeh, M. A genetic algorithm optimization approach for smart energy management of microgrids. *Renewable Energy* **197**, 852–863 (2022).

[16] Stefano Leonori, M., Paschero, F. M. F., Mascioli, A. & Rizzi Optimization strategies for microgrid energy management systems by genetic algorithms. *Appl. Soft Comput.* **86**, 1568–4946 (2020).

[17] Hai, T., Alazzawi, A. K., Zain, J. M. & Muranaka, K. Efficient short-term energy management of a renewable energy integrated microgrid using modified manta ray foraging optimization. *Sustainable Energy Technol. Assess.* **54**, 102802 (2022).

[18] Moghaddam, M. J. H. et al. Optimal sizing and energy management of stand-alone hybrid photovoltaic/wind system based on hydrogen storage considering LOEE and LOLE reliability indices using flower pollination algorithm. *Renewable Energy* **135**, 1412–1434 (2019).

[19] Kujur, S., Dubey, H. M. & Salkuti, S. R. Demand response management of a residential microgrid using chaotic aquila optimization. *Sustainability* **15**, 1484 (2023).

[20] Cerna, F. V., Dantas, J. T., Naderi, E. & Contreras, J. Optimal strategy to reduce energy waste in an electricity distribution network through direct/indirect bulk load control. *Energy* **294**, 130835 (2024).

[21] Cerna, F. V. et al. Optimal operating scheme of neighborhood energy storage communities to improve power grid performance in smart cities. *Applied Energy* **331**, 120411 (2023).

[22] Dehghani, M., Trojovská, E., Trojovský, P. & Malik, O. P. OOBO: A New Metaheuristic Algorithm for solving optimization problems. *Biomimetics*. **8**, 468 (2023). 37887599 10.3390/biomimetics8060468 PMC10604662

[23] Huang, X., Xu, R., Yu, W. & Wu, S. Evaluation and Analysis of Heuristic Intelligent Optimization Algorithms for PSO, WDO, GWO and OOBO. *Mathematics*. **11**, 4531 (2023).

[24] Huang, W. T., Yao, K. C. & Wu, C. C. Using the direct search method for optimal dispatch of distributed generation in a medium-voltage Microgrid. *Energies*. **7**, 8355–8373 (2014).

[25] Zhang, J., Huang, L., Shu, J., Wang, H. & Ding, J. Energy management of pv-diesel-battery hybrid power system for Island stand-alone micro-grid. *Energy Procedia* **105**, 2201–2206 (2017).

[26] Shi, W., Li, N., Chu, C. C. & Gadh, R. Real-time energy management in microgrids. *IEEE Trans. Smart Grid*. **8**(1), 228–238 ( 2017).

[27] Shi, W., Li, N., Chu, C.-C. & Gadh, R. Real-time energy management in microgrids. *IEEE Trans. Smart Grid* **8**(1), 228–238 (2017).

[28] Ahmed, A. et al. Bi-level energy management system for optimal real time operation of grid tied multi-nanogrids. *Electr. Power Syst. Res.* **214**, 0378–7796 (2023).

[29] Asano, H., Takahashi, M. & Ymaguchi, N. Market potential and development of automated demand response system. In *IEEE Power and Energy Society General Meeting* 1–4 (IEEE, 2011).

[30] Naderi, E., Mirzaei, L., Pourakbari-Kasmaei, M., Cerna, F. V. & Lehtonen, M. Optimization of active power dispatch considering unified power flow controller: Application of evolutionary algorithms in a fuzzy framework. *Evol. Intel.* **17**(3), 1357–1387 (2024).

